# Ambiphilic behavior of hydrogen in trisubstituted silanes induced by substituent controlled polarity inversion

**DOI:** 10.1038/s42004-026-01980-1

**Published:** 2026-03-20

**Authors:** Vítězslav Hrubý, Debashree Manna, Rabindranath Lo, Pavel Hobza

**Affiliations:** 1 Vítězslav Hrubý Regional Centre of Advanced Technologies and Materials, Czech Advanced Technology and Research Institute (CATRIN), Palacký University Olomouc, Olomouc, Czech Republic; 2 Dr. Rabindranath Lo, Dr. Debashree Manna, and Prof. Pavel Hobza Institute of Organic Chemistry and Biochemistry, Czech Academy of Sciences, Prague, Czech Republic; 3 Prof. Pavel Hobza IT4Innovations, VŠB-Technical University of Ostrava, Ostrava, Poruba Czech Republic

**Keywords:** Chemical physics, Computational chemistry

## Abstract

Atomic partial charges are local, model-dependent descriptors that often fail to capture the global electrostatic environment governing chemical reactivity. This study demonstrates that the molecular electrostatic potential (ESP) at the Si–H hydrogen in trisubstituted silanes reliably predicts electrophilic versus nucleophilic behavior, whereas local charges alone can mislead. Electron-donating substituents generate hydridic hydrogens with negative ESP near hydrogen, favoring nucleophilicity. Electron-withdrawing substituents typically leave hydrogen with a negative local charge but generate a positive ESP region along the Si–H axis, promoting electrophilic character. Increasing solvent polarity amplifies these contrasts, driving minima more negative for electron-donating substituents and maxima more positive for electron-withdrawing substituents. The resulting positive region near hydrogen in electron-withdrawing silanes is directionally σ-hole-like, yet unlike classical σ-holes from lone-pair depletion, it reflects a collective molecular electrostatic effect. These findings highlight the importance of global electrostatics for understanding structure–reactivity relationships and predicting substituent-controlled behavior in silicon hydrides.

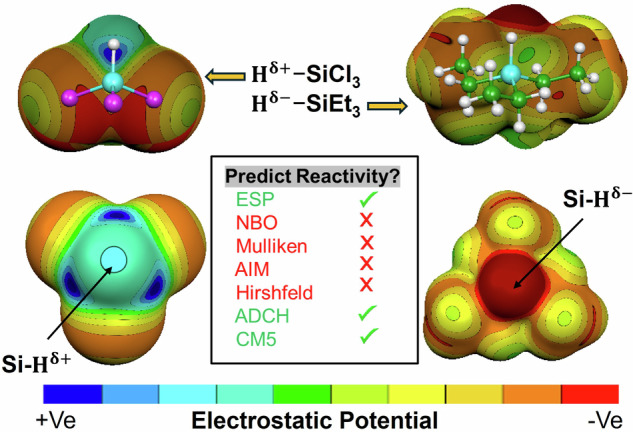

## Abstract (web summary)

Atomic partial charges are local, model-dependent descriptors that often fail to capture the global electrostatic environment governing noncovalent interactions and reactivity. Here, through calculations and NMR experiments, the authors evaluate atomic charges and molecular electrostatic potential (ESP) extrema in trisubstituted silanes in the gas phase and in benzene and o-dichlorobenzene solvents, showing that the ESP at Si–H is a more decisive predictor of molecular reactivity, whereas local charges alone are misleading.

## Introduction

The distribution of electron density around a molecule plays a crucial role in determining its chemical reactivity and intermolecular interactions^1,2^. Traditionally, this distribution is analyzed by calculating atomic charges using various theoretical models^3,4^. However, atomic charges are not unique quantum-mechanical observables; instead, they result from arbitrary partitions of the total electron density among atoms. Consequently, different population analysis schemes (e.g., Mulliken, NPA, QTAIM, ESP-derived charges) can produce substantially different absolute values and, in some cases, even qualitatively different trends across the same molecular series. Moreover, atomic charges are inherently local and spatially averaged descriptors, assigning a single scalar value to an atom that integrates over the entire region associated with that atom. In doing so, they necessarily obscure directional features and variations in electron density within and around the atomic domain. The molecular electrostatic potential, by contrast, is defined at each point in space and therefore provides a higher-resolution, spatially continuous description of the combined electrostatic contributions from nuclei and electrons. While both atomic charges and ESP depend on the same underlying nuclear and electronic distributions, ESP directly reflects how this distribution manifests in the surrounding space, capturing anisotropy, polarization, and site-specific variations that are critical for intermolecular recognition and chemical reactivity. In this sense, ESP does not average over an “atomic volume,” but instead maps the electrostatic landscape experienced by an approaching reactant or probe. Because many intermolecular interactions are governed by electrostatic complementarity at specific spatial locations rather than by atom-centered averages, ESP can provide a more physically transparent framework for analyzing polarization effects and interaction preferences than atomic charge models. The conceptual basis for using ESP as a descriptor of intermolecular interactions and reactivity has been discussed extensively in the literature^5–12^.

A comprehensive understanding of a molecule’s reactivity and its propensity for noncovalent interactions requires considering both atomic partial charges and the overall topology of the ESP. Generally, the regions of maximum and minimum ESP (*V*_s,max_ and *V*_s,min_) coincide with the most positive and negative atomic charges, respectively. A clearer distinction may be articulated by emphasizing that the electrostatic potential reflects the electron distribution with higher spatial resolution, whereas atomic charges inherently represent an average over the finite spatial extent of an “atom.”^5–10^ Notable exceptions exist, however, where atomic charges fail to capture the true ESP distribution. In such cases, it is the ESP, not the atomic charges, that determines whether a molecule behaves as an electrophile or a nucleophile.

One of the clearest examples of this mismatch is the anisotropic electron density around a halogen covalently bonded to carbon (R–X), which creates a positive ESP region (the σ-hole) opposite the R–X bond^13^. This concept was extensively developed by Peter Politzer, Jane S. Murray, and Timothy Clark, who advocated the umbrella term “σ-hole bonding” for interactions involving such regions, encompassing halogen, chalcogen, pnictogen, tetrel, and triel bonds^14–16^.

The σ-hole concept was introduced to rationalize the counterintuitive electrophilicity of halogens covalently bound to carbon. Despite their higher electronegativity and the resulting net negative atomic charge, these halogens often act as electrophiles. A positive σ-hole resolves the paradox, a localized maximum in the ESP along the extension of the R–X bond, which engages attractively with electron-rich sites. This behavior highlights the limitations of atom-centered charges and underscores the importance of ESP in accurately describing reactivity and noncovalent interactions^10^.

The situation for chemical bonds involving the lightest element, hydrogen, is notably different. In bonds such as F-H, O-H, N-H, and also C-H, the heavier atom (Y) is generally more electronegative than hydrogen. Consequently, the Y-H bond is polarized towards the heavier atom (Y), rendering the hydrogen atom partially positive, or protic. This protic nature of hydrogen remains generally consistent regardless of the substituents attached to the heavy atom or the broader molecular environment.

By contrast, for the heavier Group 14 elements, i.e., silicon, germanium, and tin, the trend reverses. Since these atoms are more electropositive than hydrogen (Pauling electronegativities: Si 1.74, Ge 2.02, Sn 1.72 vs H 2.20), Y–H bonds are polarized toward hydrogen, giving H a negative partial charge, making them hydridic in nature. Such hydridic hydrogen forms non-covalent complexes with various types of electron acceptors, including positive σ-, π-, and p-holes confirmed by theoretical and experimental studies^17,18^. Furthermore, silanes act as a versatile hydride source for catalytic reactions^19^. Crucially, however, a negative atomic charge on hydrogen does not necessarily imply a negative ESP in its vicinity. Substituents on the tetrel center might markedly reshape the local ESP at H, and even invert its sign.

In unsubstituted silane (SiH₄), hydrogen is hydridic (q = −0.141), yet the corresponding *V*_s,max_ in the vicinity of H is slightly positive ( + 1.73). This trend was also previously observed by Steve Scheiner (natural charge, q = −0.156 and *V*_s,max_ =+1.50)^20^. In substituted silanes, the hydrogen typically retains a negative partial charge, but the ESP near H can change dramatically with the nature of the substituents. Consequently, the region around H may be either ESP-positive or ESP-negative, enabling the silane hydrogen to behave as an electrophile or a nucleophile under different conditions.

For completeness, we also examined in this study other hydrogen-containing bonds that could exhibit related behavior. In Al–H bonds, the strong electropositivity of aluminum renders hydrogen hydridic, and the ESP near H can switch sign with substitution, closely paralleling the Si–H case. By contrast, C–H and P–H bonds remain consistently protic with positive ESP near hydrogen, regardless of the substitutions considered. Thus, within the neutral molecules, silicon represents a rare nonmetal capable of displaying ambiphilic behavior at the Y–H bond.

This study investigates the electronic structure and ambiphilic behavior of Si-H in trisubstituted silanes in the gas phase and in two solvents, benzene (BEN) and o-dichlorobenzene (o-DCB), with computational results complemented and validated by experimental NMR spectroscopy.

## Results and discussion

**Fig. 1:**
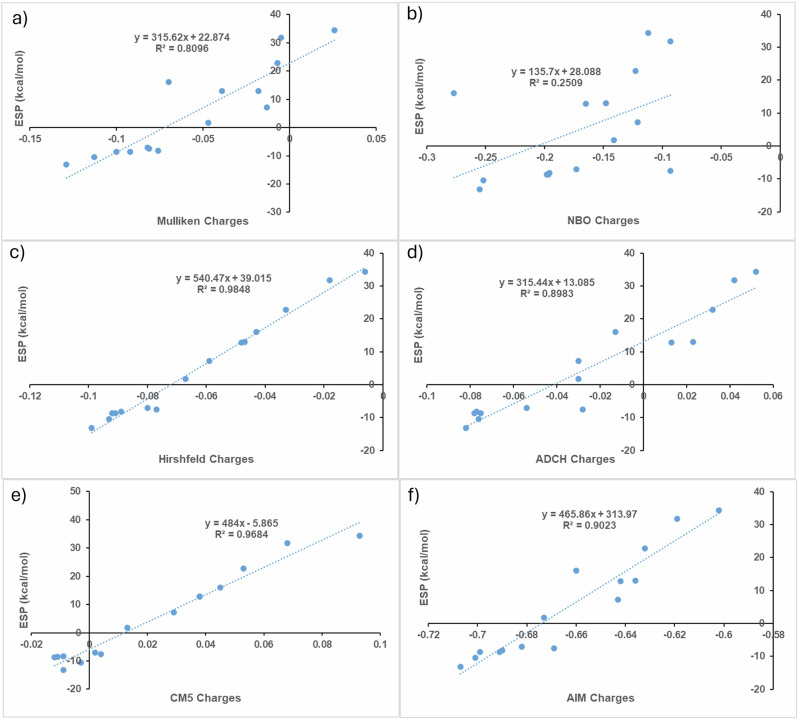
Correlation plots of various charges versus ESP (kcal/mol) calculated at the PBE0-D3/def2-TZVPP level of theory in the gas phase of silanes. **a** Mulliken charges versus ESP, **b** NBO charges versus ESP, **c** Hirshfeld charges versus ESP, **d** ADCH charges versus ESP, **e** CM5 charges versus ESP and **f** AIM charges versus ESP.

The structures and charge distributions of trisubstituted silanes were investigated by DFT at the PBE0-D3/def2-TZVPP level^21–23^. Additional DFT calculations were carried out using the M06-2X-D3/def2-TZVPP and ωB97M-V/def2-TZVPP functionals^24,25^, which employ different treatments of exchange–correlation effects, nonlocal interactions, and electronic polarization. To further assess the reliability of density-derived properties, higher-level benchmark calculations were performed at the DLPNO-CCSD(T)/def2-TZVP level^26–28^. Calculations were performed in the gas phase and in BEN and o-DCB using a COSMO continuum model (dielectric constants 2.27 and 9.99 for BEN and o-DCB, respectively)^29^. The substituents in the X₃SiH systems span a wide range of electronic effects, from electron-withdrawing groups (EWGs: F, Cl, Br, CF₃, CN, NO₂, C₆F₅) to electron-donating groups (EDGs: Me, Et, *i*Pr, Ph, NH₂, NMe₂), where X denotes the various substituents. For each silane, we determined atomic charges and the extrema of the electrostatic potential on the molecular surface (*V*_s,max,_ *V*_s,min_; Tables S1–S4). Optimized structures are shown in Fig. S1. We first discuss hydrogen atomic charges computed using NBO (Natural Bond Orbital), Mulliken, Hirshfeld, ADCH (atomic dipole moment corrected Hirshfeld), and CM5 (Charge Model 5) schemes (Tables S1–S4). For completeness, hydrogen charges were also evaluated using the CHELPG (CHarges from ELectrostatic Potentials using a Grid-based) and QTAIM (Quantum Theory of Atoms in Molecules) approaches. Notably, recent work^4^ has shown that electron-density-based quantum mechanical charge schemes, including ADCH and CM5, exhibit high Pearson correlations ( ≥ 0.8) with experimental partial charges determined via electron diffraction. This strong agreement was observed across a variety of crystalline organic compounds, including the antibiotic ciprofloxacin and the amino acids histidine and tyrosine, demonstrating the reliability of these computational methods for reproducing experimentally observed charge distributions. Among the tested schemes, NBO yields the largest charge magnitudes, whereas Mulliken and Hirshfeld produce smaller and largely similar values. In BEN and o-DCB, hydrogen charges become less negative for EWG-substituted silanes and more negative for EDG-substituted silanes. Notably, for EWG-substituted systems, the hydrogen charges calculated from NBO, Mulliken, Hirshfeld at hydridic H do not correlate with the ESP, whereas for EDG-substituted silanes, they correlate well. However, the partial charges derived from the ADCH and CM5 methods exhibit a comparatively stronger correlation with the ESP in EWG-substituted systems^30^. Both methods employ Hirshfeld population analysis, which partitions the molecular electron density to determine partial atomic charges. Linear regression analyses were performed, and the resulting correlation coefficients (R² values) indicate that the CM5, Hirshfeld, and ADCH charge schemes exhibit the strongest correlations with the ESP values for all DFT and DLPNO-CCSD(T) methods employed in this study (Fig. 1 and S2–S12). In contrast, other charge models, including CHELPG, fail to consistently reproduce the observed trends across the full range of substituents (Figs. S7–S12). Notably, even within the CM5 and ADCH frameworks, there are cases in which the sign of the assigned atomic charge does not correspond to the sign of the local ESP. Hirshfeld charges, in particular, systematically assign a negative charge to the hydrogen atom irrespective of the substituent, whereas the ESP at the hydrogen site spans both positive and negative values depending on the electronic environment. To assess surface sensitivity, we show that the correlations between atomic charges and the ESP values at the hydrogen sites preserve the same relative trends upon variation of the isodensity value within a reasonable range (e.g., 0.0005–0.002 a.u.) (Fig. S13).

A central result is the mismatch that can arise between local atomic charge and the global ESP at hydrogen. For EDG-substituted silanes, however, the picture is straightforward: all charge schemes (NBO, Mulliken, Hirshfeld, ADCH, CM5, AIM, CHELPG) assign a negative partial charge to H, and the surface ESP at H is likewise negative. Accordingly, H behaves as a nucleophilic, hydridic site. The ESP around H is anisotropic: it is least negative at the axial “pole” along the Si–H bond and most negative around the equatorial “belt” (Tables S1–S4), where *V*_s,min_ is located (see later).

For EWG-substituted silanes, an intriguing inversion appears. Most of the charge-partitioning schemes still assign H a negative partial charge, seemingly indicating a nucleophilic site, but the surface ESP near H tells a different story: *V*_s,max_ along the Si–H axis is positive, marking H as electrophilic. Strong electron withdrawal by the substituents, together with the resulting positive polarization of the silicon center, depletes electron density in the Si–H bond direction and creates a σ-hole–like positive lobe at H. The local charge on H remains negative because, within the Si–H bond, H still integrates to slightly more electron density than a neutral atom. Nonetheless, the global electrostatic environment is dominated by the surrounding framework, so these hydrogens behave as electrophiles in their interactions.

The data make this clear: in EWG-substituted silanes, *V*_s,max_ at H is positive, signaling an electron-poor region, even though the atomic charge on H is negative. Crucially, in the present system, it is the sign of the ESP at H, not q(H), that tracks reactivity. To strengthen this point within the scope of the present work, we now explicitly show that the protonic hydrogen in Cl_3_SiH, with a positive ESP bonded to the nitrogen center via interaction with the lone pair of NH₃, whereas the hydridic hydrogen in Me_3_SiH with a negative ESP bonded to the iodine centre of ICF₃, consistent with the sign of the ESP at these sites (Fig. S14). Angular interaction scans were also performed for both cases to assess interaction directionality. Electrophilic (protic) behavior of Cl_3_SiH hydrogen has been supported by an early experimental study by Smith and coworkers^31^. In particular, NMR investigations of tertiary amine–trichlorosilane systems in acetonitrile revealed the disappearance of the Si–H resonance upon mixing equimolar amounts of amine and HSiCl₃, consistent with rapid proton exchange and the formation of ammonium–siliconate ion pairs. These studies established that the Si–H hydrogens in HSiCl₃ can exhibit proton-like behavior. Although the work did not invoke electrostatic potential analysis, their conclusions are fully consistent with a description in which the hydrogen atom in EWG-substituted silanes possesses a positive electrostatic region, a feature that cannot be captured by atom-centered partial charges alone. The Me_3_SiH···ICF_3_ complex in the solid phase has recently been studied by our group via experimental IR spectra^32^. Thus, a negative ESP at H (typical for EDG-substituted silanes) corresponds to a nucleophilic, hydridic hydrogen capable of donating electron density. A positive ESP at H (typical for EWG-substituted silanes) marks an electrophilic, electron-deficient hydrogen, akin to a protic site. Relying on local charges alone would incorrectly label all these silanes as bearing nucleophilic (hydridic) hydrogens; the ESP analysis instead reveals a sharp division, EDG-silanes maintain nucleophilic H, whereas EWG-silanes flip to electrophilic H. The conceptual basis for using ESP as a descriptor of intermolecular interactions and reactivity has been discussed by Politzer and coworkers^5–9^.

A natural question is whether the ambiphilic behavior observed for Si–H, in which substitution toggles the hydrogen between nucleophilic and electrophilic character, also occurs for other Y–H bonds, particularly those with small electronegativity differences between Y and H. Tables S5–S10 compile partial atomic charges and ESP values (PBE0-D3/def2-TZVPP) for X₃C–H, X₃Ge–H, X₃Sn–H, X₃Pb–H, X₂Al–H, and X₂P–H with both EWG and EDG substituents. Here, only carbon is more electronegative than hydrogen; all other elements considered are more electropositive. Using Δχ ≡ χ(H) − χ(X), the differences are −0.30 (C), +0.46 (Si), +0.18 (Ge), +0.48 (Sn), +0.64 (Pb), +0.73 (Al), and +0.14 (P).

For X₃C–H, hydrogen is protic for all substituents considered and the ESP at H is positive, consistent with electrophilic behavior. X₂P–H systems likewise show systematically positive ESP at H and are electrophilic. Upon moving to solvent (BEN, o-DCB), both q(H) and *V*_s,max_ shift to more positive values in these two families.

X₂Al–H behaves differently. Here, H is uniformly hydridic, yet ESP at H depends on substitution: it is positive for EWG-substituted alanes and negative for EDG-substituted alanes, mirroring the X₃Si–H case. Accordingly, EDG-substituted alanes feature nucleophilic H, whereas EWG-substituted alanes display electrophilic H. Increasing solvent polarity drives ESP at H more negative for EDG cases and more positive for EWG cases. Solvent effects were not considered in all remaining molecules, and only the gas phase calculations were performed.

These results highlight the superiority of a global, ESP-based description over purely local charge analyses for understanding and predicting reactivity. In particular, here, ESP mapping accurately distinguishes silanes that act as hydride donors from those exhibiting protic, electrophilic behavior, a distinction that partial atomic charges alone fail to capture. While local charge analysis would suggest uniformly nucleophilic behavior for all silanes, a global ESP-based approach correctly predicts electrophilic behavior for EWG-substituted silanes and nucleophilic behavior for EDG-substituted silanes. To validate this, we examined silanes in two aprotic solvents, BEN and o-DCB. Upon moving from the gas phase to solvent, *V*_s,max_ at H shifts more positive for EWG-substituted silanes and more negative for EDG-substituted silanes.

To support these theoretical trends, we recorded ¹H NMR spectra for four commercially available silanes, ((C₆F₅)₃Si–H, (Me₃Si)₃Si–H, Et₃SiH, and Ph₃SiH) in BEN and o-DCB. As shown in Tables S1–S4, (C₆F₅)₃Si–H exhibits a positive ESP at H, whereas the others show a negative ESP at H. The experimental chemical shifts are summarized in Table 1 and S11 and compared with values computed at the PBE0-D3/def2-TZVPP level using the COSMO continuum model. For silanes bearing flexible substituents (Me₃Si–, Et–, Ph–, and C₆H₅–), the experimentally observed chemical shifts are likely to reflect Boltzmann-weighted averages over multiple conformations rather than values associated with a single optimized structure. Accordingly, the computational protocol was adopted to account for conformational effects explicitly. Multiple low-energy conformers were sampled from molecular dynamics (MD) simulations, and the Gauge-Including Atomic Orbital (GIAO) chemical shifts^33^ were calculated for representative snapshots. Boltzmann-weighted averages of the resulting chemical shifts were then obtained for each silane (Table 1 and S12). The study was further extended to explicitly include solvent molecules. Boltzmann-weighted averages of the chemical shifts were determined for the silanes by considering configurations containing 15 explicit solvent molecules (Table S13).

**Fig. 2:**
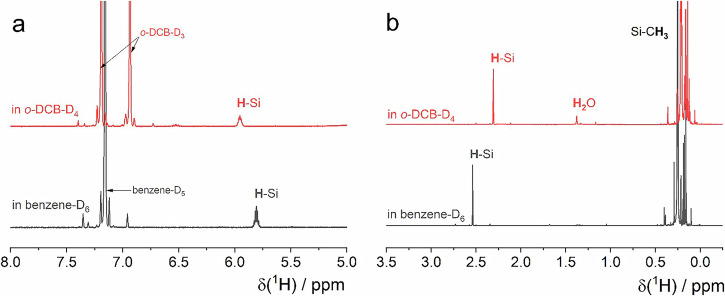
The ^1^H NMR spectra in benzene and o-DCB medium. **a** (C_6_F_5_)_3_Si-H, **b** (Me_3_Si)_3_Si-H.

Protic hydrogens typically exhibit modest, polarity-dependent solvent shifts and tend to move downfield as solvent polarity increases^34,35^. We observe this behavior for the EWG-substituted silane (C₆F₅)₃Si–H: *V*_s,max_ at H becomes more positive upon going from BEN to o-DCB, and a downfield shift of the ¹H resonance (Table 1, Fig. 2). In contrast, EDG-substituted silanes ((Me₃Si)₃Si–H, Et₃SiH, and Ph₃SiH) show the opposite trend (Table 1, Fig. 2), in line with their negative *V*_s,max_ values: increasing solvent polarity drives *V*_s,max_ more negative and shifts the ¹H signal upfield. In NMR, the observed chemical shift (δ) depends on how much the local electrons oppose the external magnetic field (B₀). A more negative ESP at H means the region around H is richer in electron density (from bond polarization and substituent effects). This increased local electron density can partially shield the nucleus from B₀. When electron density circulates under B₀, it generates an induced magnetic field (B_ind_) that opposes B₀ at the nucleus. Thus, higher electron density reduces the effective magnetic field at the nucleus (B_eff_ = B₀ – B_ind_). This means the nucleus resonates at a higher field strength (upfield) and shows a lower δ value (shielding). In contrast, a positive ESP at H means electron density is drawn away, leaving H electron-poor. With fewer shielding electrons, the nucleus feels more of B₀. This results in deshielding or downfield shift (larger δ) of H. Atomic charges are intrinsically local properties, whereas both the ESP and NMR chemical shifts capture not only local electron density but also the broader molecular environment and intermolecular influences. Thus, ESP and NMR chemical shifts show consistent trends and reinforce each other, in line with the expectation. Since both BEN and o-DCB are aromatic, differential ring-current effects are minimized, so the observed chemical-shift changes can be attributed primarily to solvent polarity. Experimental details and individual spectra are provided in the Supporting Information (Figs. S15–S53).

**Table 1:** The proton chemical shifts of silanes, as well as their corresponding differences in two solvents, calculated at the PBE0-D3/def2-TZVPP level of theory using the COSMO continuum solvation model in benzene and o-DCB

Silane	Medium	Calculated ^1^H chemical shifts^a^		Experimental ^1^H chemical shifts		Experimental ^29^Si chemical shifts	
		^1^H δ ppm	Relative Δδ ^1^H ppm	^1^H δ ppm	Relative Δδ ^1^H ppm	^29^Si δ ppm	Relative Δδ ^29^Si ppm
(C_6_F_5_)_3_Si-H	BEN	6.609	0.002	5.81	0.14	−54.7	0.4
	o-DCB	6.611		5.95		−54.3	
(Me_3_Si)_3_Si-H	BEN	2.636	−0.123	2.54	−0.23	−115.56	0.22
	o-DCB	2.513		2.31		−115.34	
Et_3_Si-H	BEN	4.016	−0.089	3.90	−0.21	0.52	-0.07
	o-DCB	3.927		3.69		0.45	
Ph_3_Si-H	BEN	6.199	−0.048	5.71	−0.21	−17.49	0.04
	o-DCB	6.151		5.50		−17.45	

^a^^1^H NMR of TMS taken as a reference with the isotropic shielding value of 31.506 ppm.

The experimental proton chemical shifts of the silanes are also provided.

¹H NMR measurements in BEN and o-DCB corroborate the divergent reactivity of EWG- and EDG-substituted silanes, supporting the ESP-based, non-local description of charge distribution.

However, in other solvents, cyclohexane-D12, CDCl_3_, and CD_2_Cl_2_ (Table S11), the theoretical trends are not strictly followed. In the case of tris(pentafluorophenyl)silane (TPFPS), little to no changes in chemical shifts are observed for non-aromatic solvents. Similar to TPFPS, EDG silanes follow this trend in aromatic solvents as well, but not strictly in non-aromatic solvents. Only tris(trimethylsilyl)silane and triethylsilane  follow all the trends well, while the trend for triphenylsilane  is slightly opposite in non-aromatic solvents. The interactions between silane hydrogen atom and aromatic and non-aromatic solvents are shown (Fig. S54). On the other hand, the ^29^Si chemical shifts (Table 1) were relatively less affected by the change of the solvent compared to the proton shifts. However, here the downfield trend is common for all negative chemical shifts (with respect to TMS), and upfield for positive chemical shifts (in the case of triethylsilane).

To further investigate the physical origin of the Si–H chemical shift trends and their relationship to the calculated electrostatic potentials (ESPs), we conducted variable-temperature (VT) ¹H NMR measurements for four representative silanes in two solvents (CD_2_Cl_2_ and benzene-D_6_). These experiments provide additional insight into the distinct components of the temperature-dependent shielding and offer an experimental handle on the factors beyond local electron density that influence the ^1^H chemical shifts. Apart from bulk magnetic susceptibility changes and vibrational averaging, producing a linear change of chemical shifts (for TMS, −5 ∙ 10^−4^ ppm/K)^36^, a 1/T term has been found to significantly influence the ^1^H δ(T) dependencies. This term represents paramagnetic/pseudocontact or Curie-law population effects^37–39^. Hence, additional mechanisms, including paramagnetic contributions influenced by virtual excitations, also affect the ¹H δ apart from electron density. Further details on the VT experimental results can be found in the ESI.

**Fig. 3:**
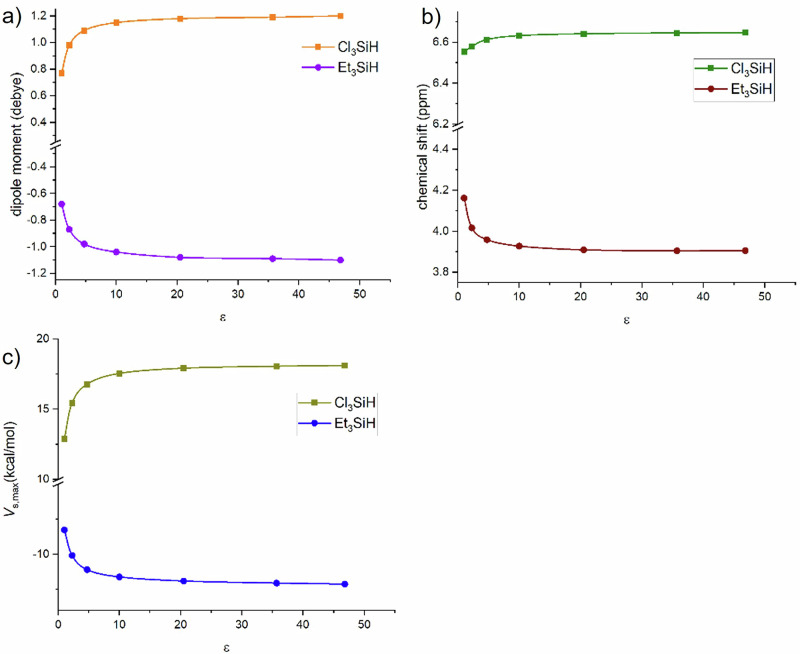
Correlation plots between various calculated properties and the solvent polarity (Ɛ) for Cl_3_SiH and Et_3_SiH. **a** dipole moment, **b** chemical shift and **c** *V*_s,max_ with solvent polarity (Ɛ).

A comprehensive computational analysis was performed for Et₃SiH and trichlorosilane in the gas phase and in four additional solvents: chloroform, acetone, acetonitrile, and DMSO. Because these calculations used the implicit COSMO model, solvent effects are governed solely by the dielectric constant (ε); features such as protic/aprotic character and aromaticity are not represented. For EWG- versus EDG-substituted silanes, the influence of solvent polarity on ¹H chemical shifts, *V*_s,max_ values, and dipole moments follows opposite trends (Fig. 3). In the EDG-substituted silane Et₃SiH, increasing ε decreases both *V*_s,max_, and the dipole moment, accompanied by an upfield shift of the Si–H ¹H resonance. Please note that the decrease in the dipole moment arises from the chosen sign convention, reflecting an opposing orientation and does not indicate a reduction in the dipole moment absolute values. In contrast, in the EWG-substituted trichlorosilane, increasing ε raises *V*_*s,max*_ and the dipole moment and produces a downfield shift of the Si–H ¹H signal. These opposing behaviors reflect the positive ESP induced by EWGs. Because the dipole vectors of EWG- and EDG-substituted silanes are oriented in opposite directions, we plot dipole moments for the EDG cases with a negative sign for consistency. As shown in Fig. 3, solvent effects are most pronounced at low ε and become negligible at higher ε.

**Fig. 4:**
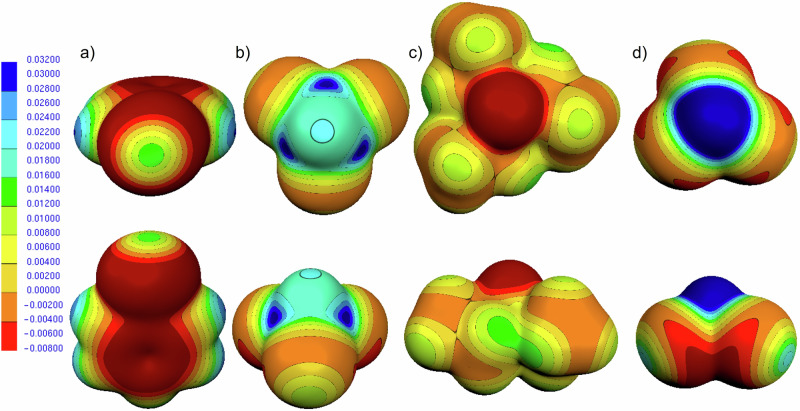
The molecular electrostatic potential maps on the 0.001 au isodensity surface, both top and side view. **a** bromobenzene and **b** Cl_3_SiH **c** Et_3_SiH **d** Cl_3_CH. The scale is in a.u.

We have shown that in EWG-substituted silanes the *V*_s,max_ near H becomes positive. This resembles the positive σ-hole observed for halogens covalently bonded to carbon. Figure 4 illustrates the parallel by comparing the anisotropic ESP in bromobenzene (Fig. 4a) and trichlorosilane (Fig. 4b). In bromobenzene, although Br bears a net negative atomic charge, a pronounced positive region, the σ-hole, appears along the extension of the C–Br bond, while a belt of negative potential lies perpendicular to this axis. Here, within the σ-hole, the ESP is uniformly positive relative to its surroundings. Trichlorosilane shows an analogous anisotropy around the hydridic hydrogen: despite a negative partial charge on H, a positive ESP “cap” is found along the Si–H axis (the pole), and even the equatorial belt remains positive (≈ + 11.1) though less than at the pole (≈ + 12.9). Consequently, the Si–H bond in such silanes acts exclusively as an electrophilic site.

In halogens such as bromine, the σ-hole arises from an anisotropic electron distribution associated with the presence of lone pairs, allowing the atom to act as an electrophile along the bond axis and as a nucleophile in the perpendicular belt. This can be rationalized either as a depletion of electron density along the C–Br extension or, qualitatively, as a change in hybridization (sp³ → sp²) that allocates five valence electrons to two lone pairs and the C–Br σ bond, leaving the third lone pair empty. This region along the bond axis constitutes the σ-hole.

Hydrogen, by contrast, has no core shell or lone pairs to redistribute; its ESP anisotropy in substituted silanes stems from the collective molecular electron distribution rather than depletion of a localized pair. In triethylsilane (Fig. 4c), H carries a negative partial charge and the nearby ESP is negative and nearly uniform; a slight anisotropy remains, with the pole ( ≈ −8.3) being marginally less negative (i.e., more positive) than the belt ( ≈ −8.6). For comparison, chloroform (Fig. 4d) features a protic hydrogen with a positive ESP in its vicinity.

## Conclusion

In summary, the molecular electrostatic potential, not local atomic charges, is the reliable descriptor for predicting and rationalizing the behavior of the Si-H of trisubstituted silanes. Across PBE0-D3/def2-TZVPP computations in the gas phase and in benzene and o-dichlorobenzene, corroborated by solvent-dependent ¹H NMR shifts, we find a clear, substituent-controlled dichotomy:

- EDG-substituted silanes: Hydrogens are hydridic and nucleophilic: q(H) < 0 and the surface ESP near H is negative. Increasing solvent polarity drives ESP more negative and shifts the Si–H resonance upfield, consistent with an electron-rich H.
- EWG-substituted silanes: Hydrogens are hydridic yet electrophilic: q(H) < 0 but the surface ESP near H is positive. Higher dielectric media make ESP more positive and shift the Si–H resonance downfield, revealing an electron-poor H. Local charges alone would incorrectly predict uniform nucleophilicity across both series.

Thus, ESP provides a robust, transferable predictor of Si–H reactivity.

- σ-hole analogy: The positive region near H in EWG-substituted silanes is σ-hole-like in directionality but does not arise from lone-pair depletion on hydrogen; it reflects a collective, molecular ESP effect.

Generalization to other Y–H bonds with small Y/H electronegativity gaps reveals consistent behavior: X₃C–H and X₂P–H remain protic with positive ESP at H (electrophilic) across all cases studied, whereas X₂Al–H mirrors Si–H by exhibiting an ESP-governed switch. Si–H is unusual among neutral nonmetals for its ability to exhibit pronounced ambiphilic behavior. Thus, global ESP features—particularly *V*_s,max,_ *V*_s,min_ at H—outperform local partial charges as predictors of electrophilic versus nucleophilic character. These insights provide practical guidelines for engineering noncovalent interactions, tuning Si–H reactivity in synthesis and catalysis, and avoiding misassignments that arise from relying solely on local atomic charges while neglecting the global electrostatic field.

## Methods

### Computational details

All molecular geometries were optimized using density functional theory (DFT) at the PBE0-D3^21,22^ level with the def2-TZVPP basis set^23^. The geometries were further optimized using the range-separated hybrid meta-GGA functional ωB97M-V, which incorporates VV10 nonlocal correlation and has been shown to provide reliable accuracy for hydrogen-bonded systems^24^. Additional density functional theory calculations were performed using the M06-2X-D3/def2-TZVPP level of theory^25^. Vibrational frequency calculations were carried out at the same level to verify that the optimized structures correspond to local minima (no imaginary frequencies). To further benchmark the reliability of density-derived properties, higher-level calculations have been performed using DLPNO-CCSD(T)/def2-TZVP level of theory^26–28^. Solvent effects of benzene and o-dichlorobenzene were included during geometry optimizations via the COSMO continuum solvation model^29^. All computations were performed using the Gaussian 16 software package^40^. Atomic partial charges were determined using NBO^41^, Hirshfeld^42^, and charge model 5 (CM5) methods^43^ within Gaussian 16. Additionally, atomic dipole–corrected Hirshfeld (ADCH) charges^44^, QTAIM charges^45^ and the maximum electrostatic potential (*V*_s,max_) near hydrogen atoms were calculated using the Multiwfn program^46^. CHELPG charges^47^ and ωB97M-V and DLPNO-CCSD(T) calculations were performed using ORCA6.0^48^. NMR chemical shifts for the optimized geometries were calculated using GIAO method^33^, with tetramethylsilane (TMS) employed as the reference standard. The molecular dynamics (MD) simulations for each silane were conducted at PBE-D3/def2-SVP level using the COSMO solvent model in ORCA6.0. In the presence of 15 explicit solvent molecules, the MD simulations were performed at PBE-D3/def2-SVP level. In the simulation, the step size was set to 1 fs, Nose–Hoover chain thermostat (NHC) with high-order Yoshida integrator^49,50^ with a time constant of 20 fs was employed to control the temperatures. The temperature for all the simulations was set at 100 K.

## Supplementary information

Supplementary Information

## Data Availability

The data that support the findings of this study are available in the supporting information of this article. The publication data are available at ZENODO 10.5281/zenodo.18852906 under the same title.
