# Supplementary material for: Ambiphilic behavior of hydrogen in trisubstituted silanes induced by substituent controlled polarity inversion
Source: Commun Chem. 2026 Mar 20;9:174. doi: 10.1038/s42004-026-01980-1 (PMC13168443; doi:10.1038/s42004-026-01980-1)
Supplement: Supplementary file 1 — Supplementary Information [file 42004_2026_1980_MOESM1_ESM.pdf]

Supplementary Information

Ambiphilic Behavior of Hydrogen in Trisubstituted Silanes Induced by Substituent Controlled Polarity Inversion

P. Hobza et al.

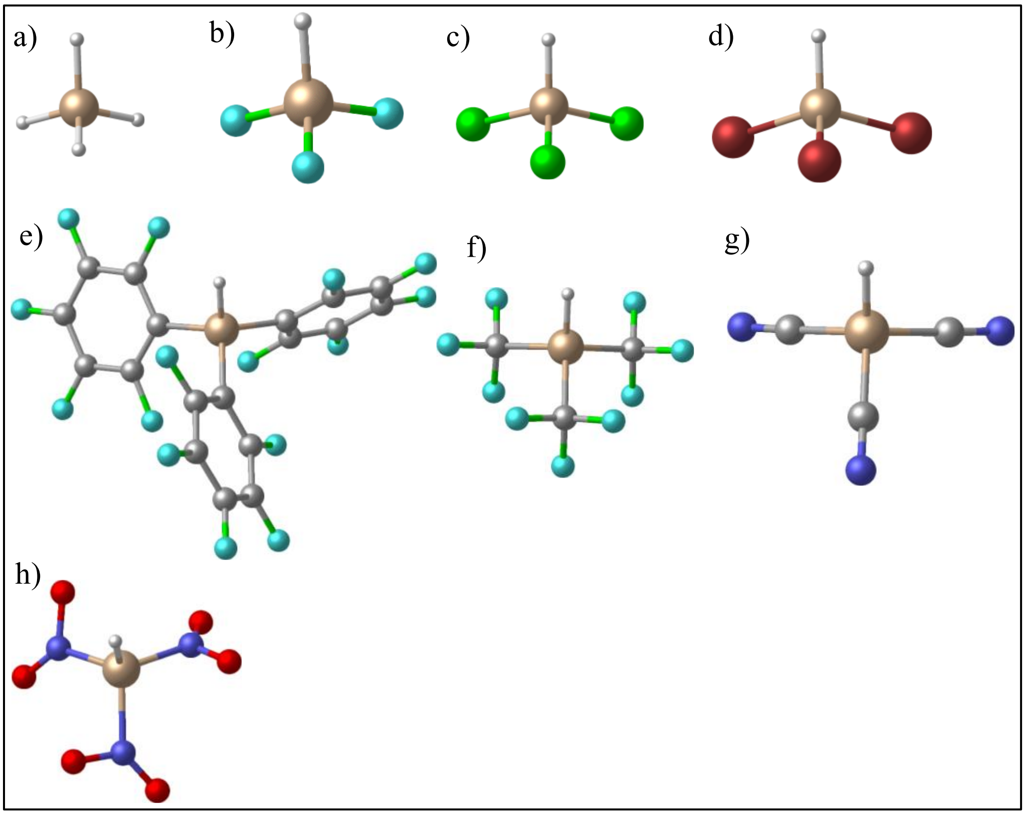
a)
b)
c)
d)
e)
f)
g)
h)

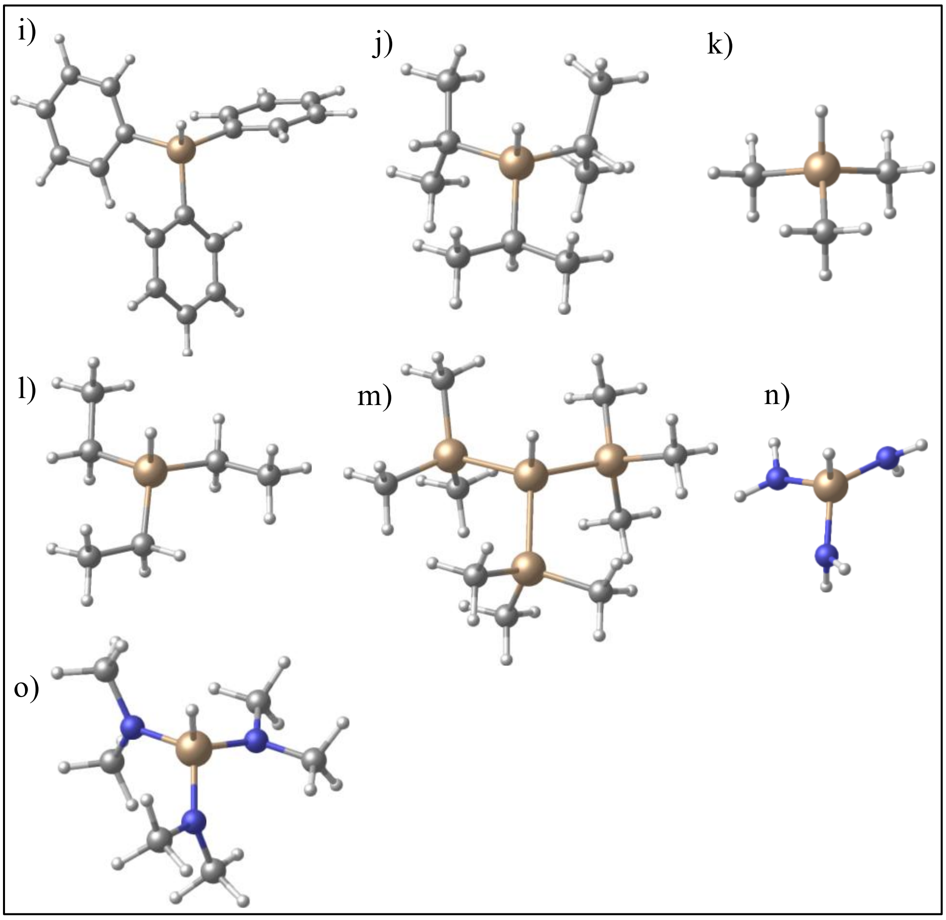
i)
j)
k)
l)
m)
n)
o)

Figure S1. The optimized geometries of silanes: a) SiH_4, b) F_3SiH, c) Cl_3SiH, d) Br_3SiH, e) $(C_6F_5)_3Si$-H, f) $(CF_3)_3SiH$, g) $(CN)_3SiH$, h) $(NO_2)_3SiH$, i) Ph_3SiH, j) $(iPr)_3SiH$, k) Me_3SiH, l) Et_3SiH, m) $(Me_3Si)_3Si$-H, n) $(NH_2)_3SiH$ and o) $(Me_2N)_3SiH$. [C: grey, N: blue, H: white, Si: golden, O: red, F: cyan, Cl: green, Br: reddish-brown]

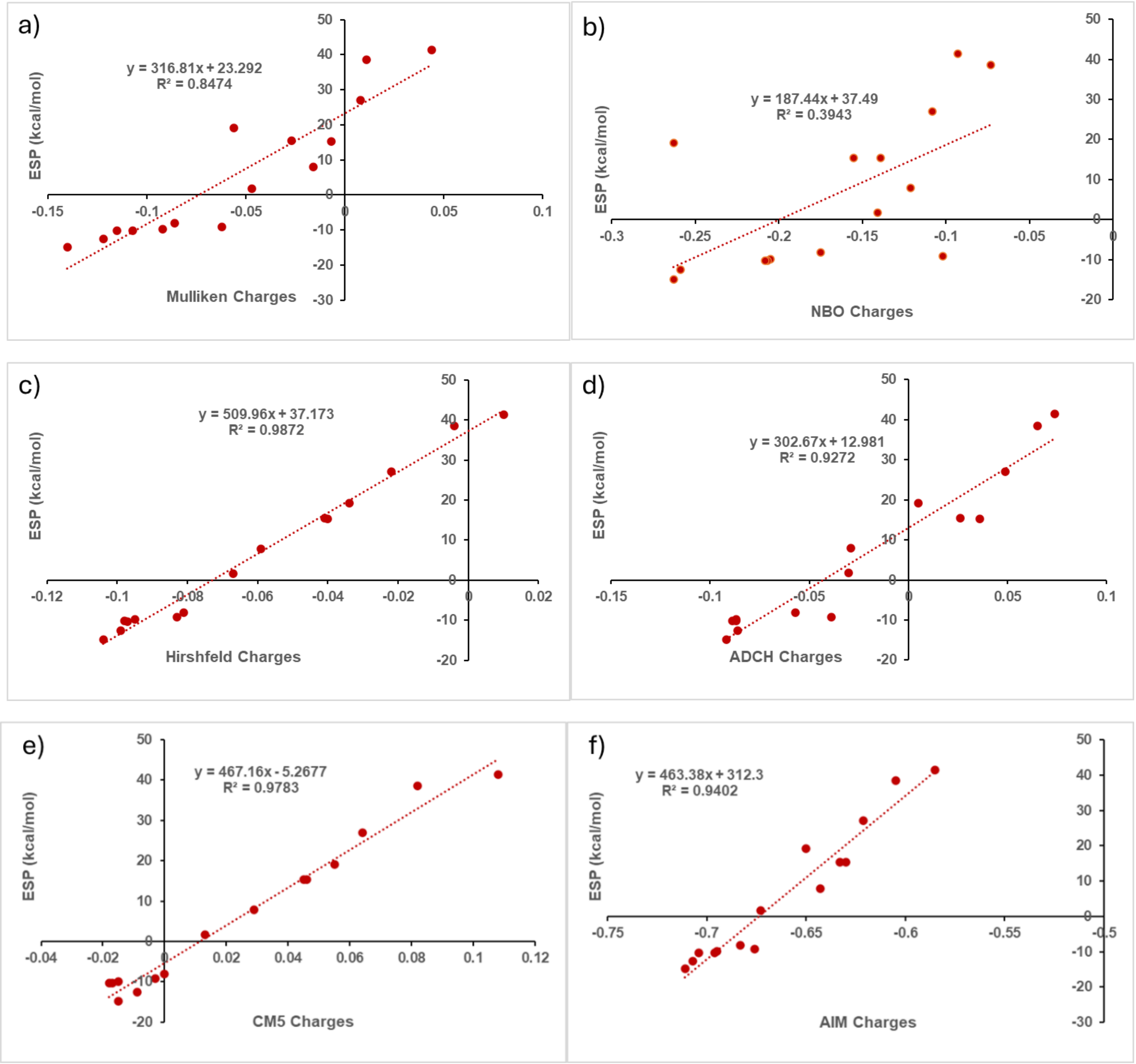

Figure S2. Correlation between all the charges and electrostatic potential (ESP, kcal mol^{-1}) evaluated at electron-density isosurface values of 0.001 a.u., computed at the PBE0-D3/def2-TZVPP level of theory using the COSMO continuum solvation model in benzene medium.

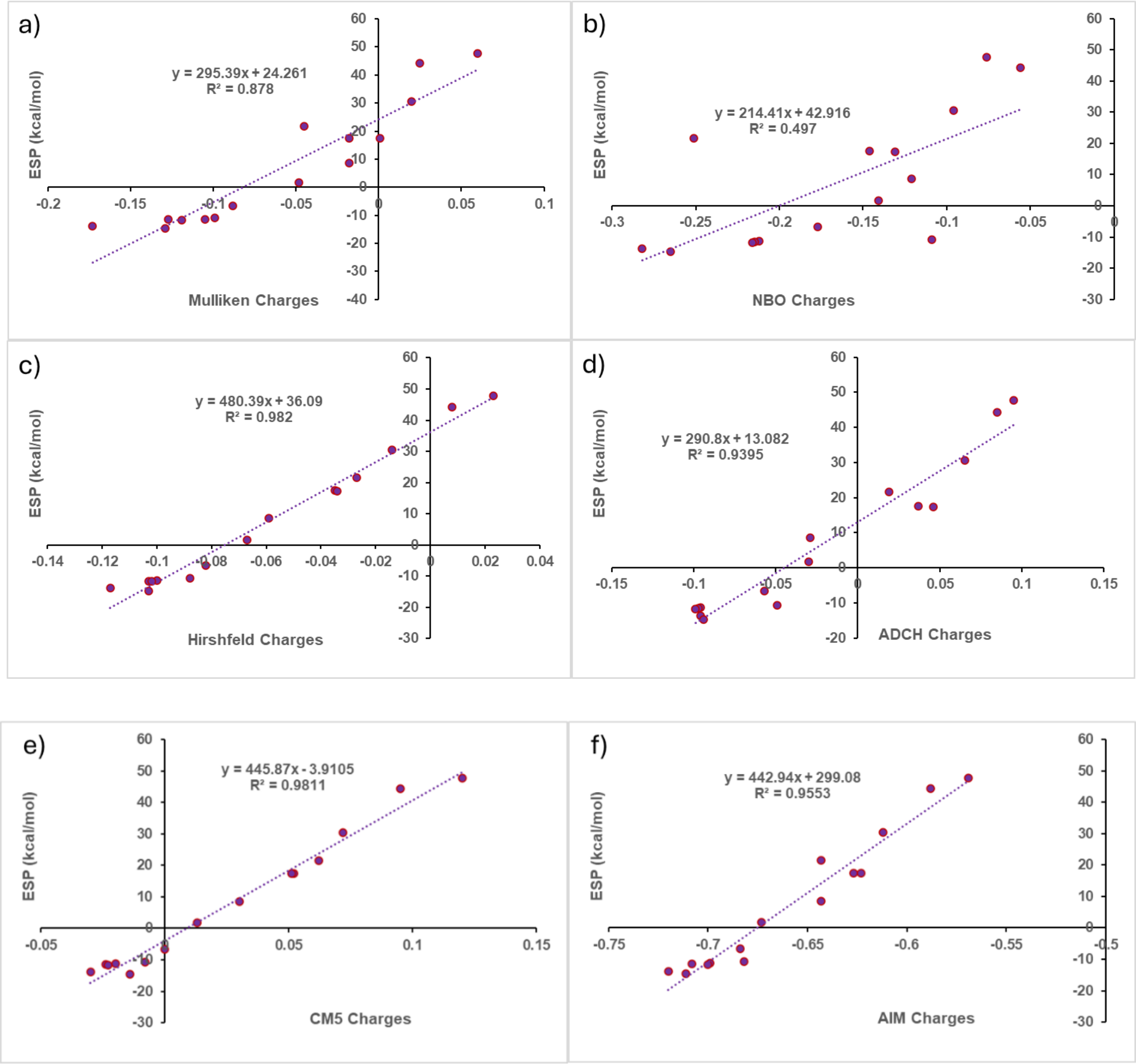

Figure S3. Correlation between all the charges and electrostatic potential (ESP, kcal mol^{-1}) evaluated at electron-density isosurface values of 0.001 a.u., computed at the PBE0-D3/def2-TZVPP level of theory using the COSMO continuum solvation model in o-DCB medium.

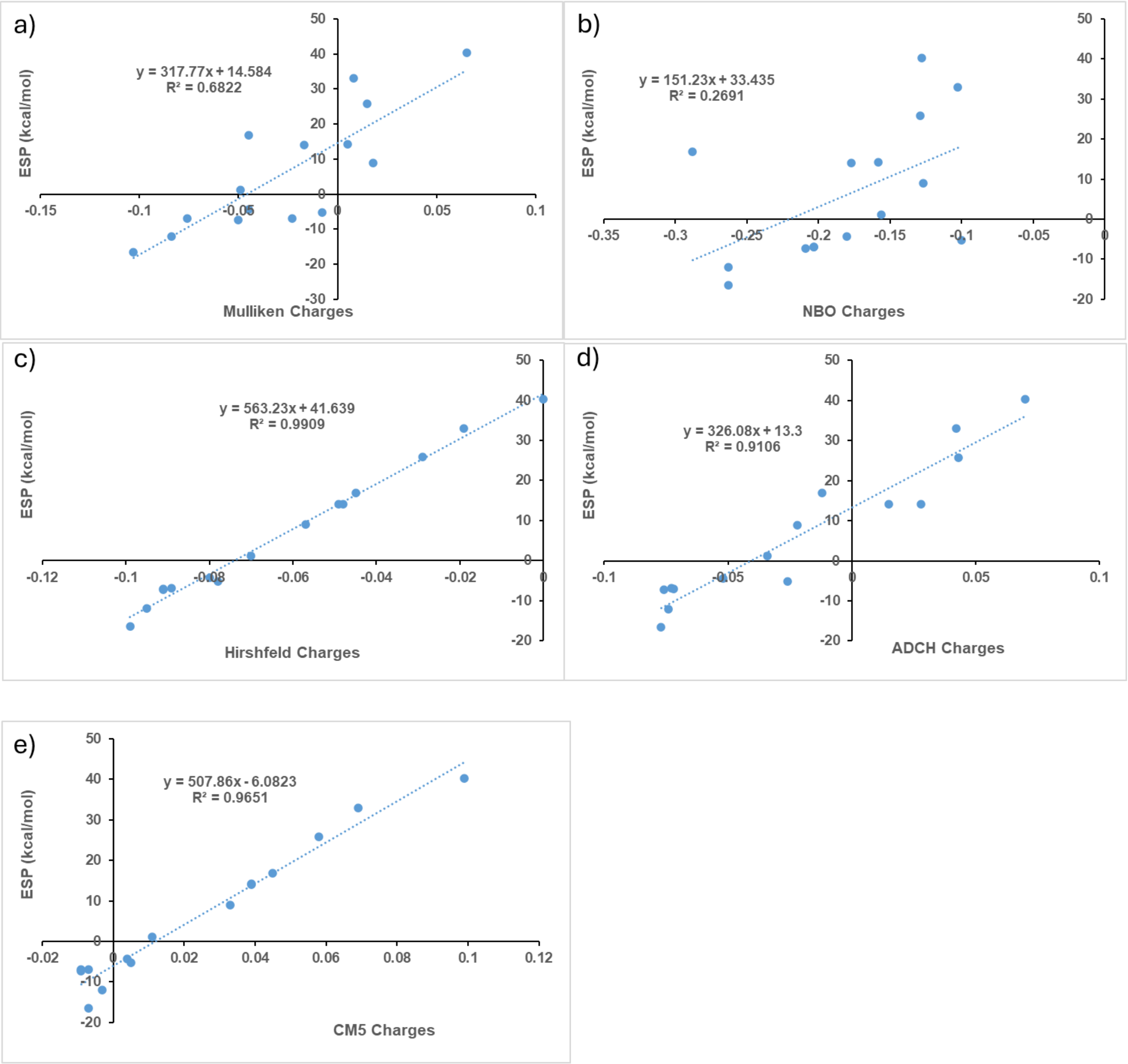

Figure S4. Correlation between all the charges and electrostatic potential (ESP, kcal mol^{-1}) evaluated at electron-density isosurface values of 0.001 a.u., computed at the M06-2X-D3/def2-TZVPP level of theory in the gas phase.

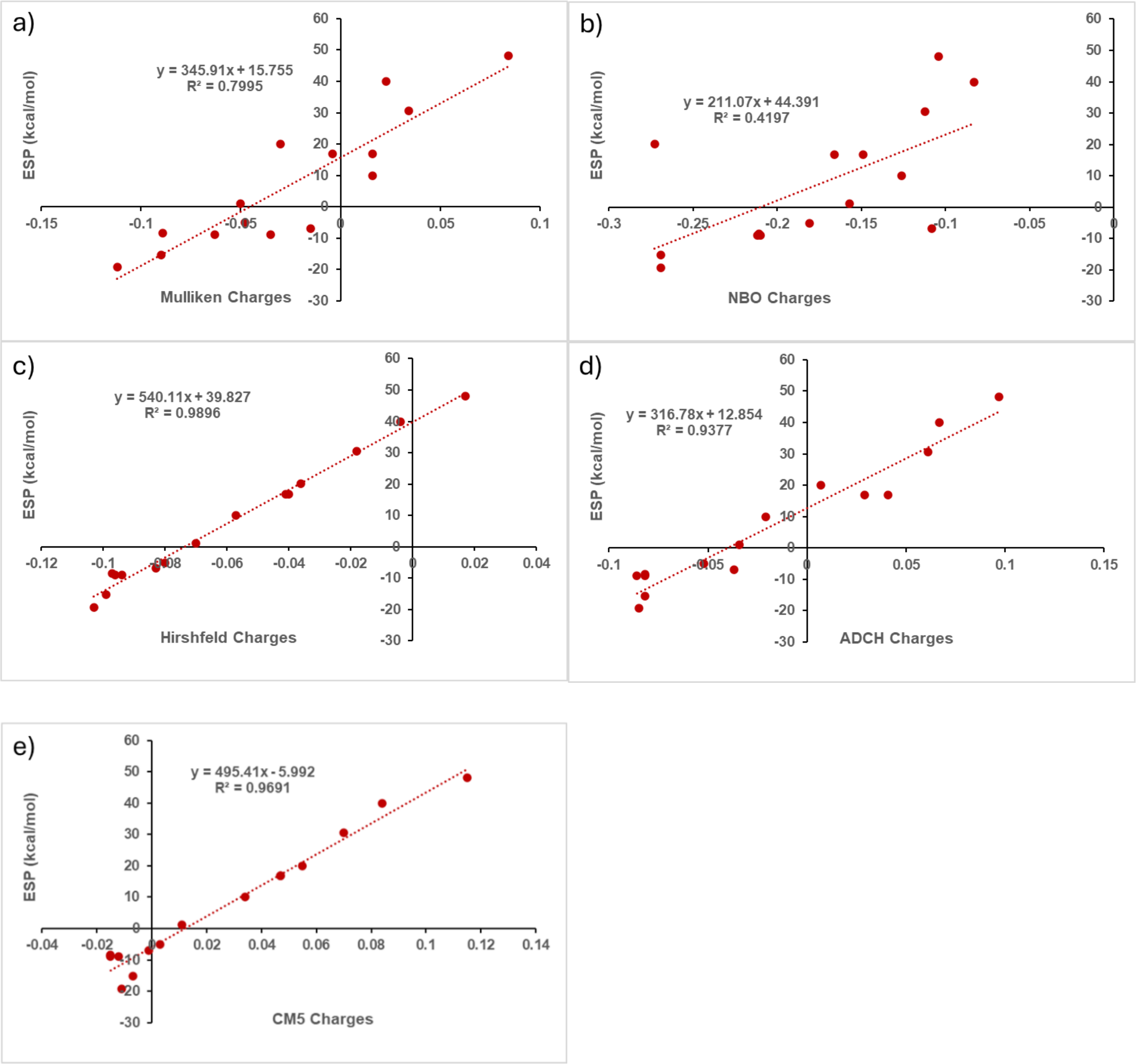

Figure S5. Correlation between all the charges and electrostatic potential (ESP, kcal mol^{-1}) evaluated at electron-density isosurface values of 0.001 a.u., computed at the M06-2X-D3/def2-TZVPP level of theory using the COSMO continuum solvation model in benzene medium.

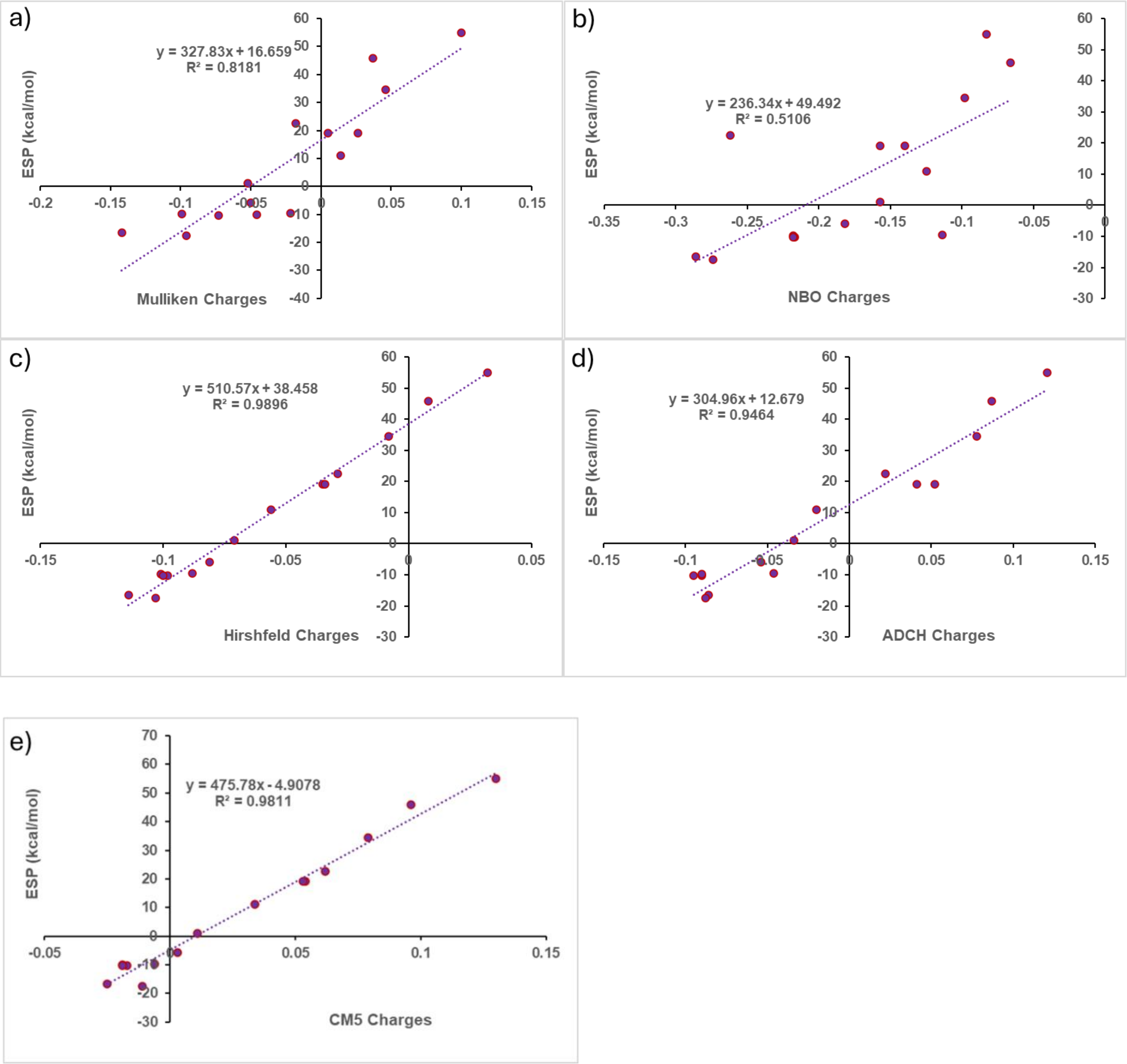

Figure S6. Correlation between all the charges and electrostatic potential (ESP, kcal mol^{-1}) evaluated at electron-density isosurface values of 0.001 a.u., computed at the M06-2X-D3/def2-TZVPP level of theory using the COSMO continuum solvation model in o-DCB medium.

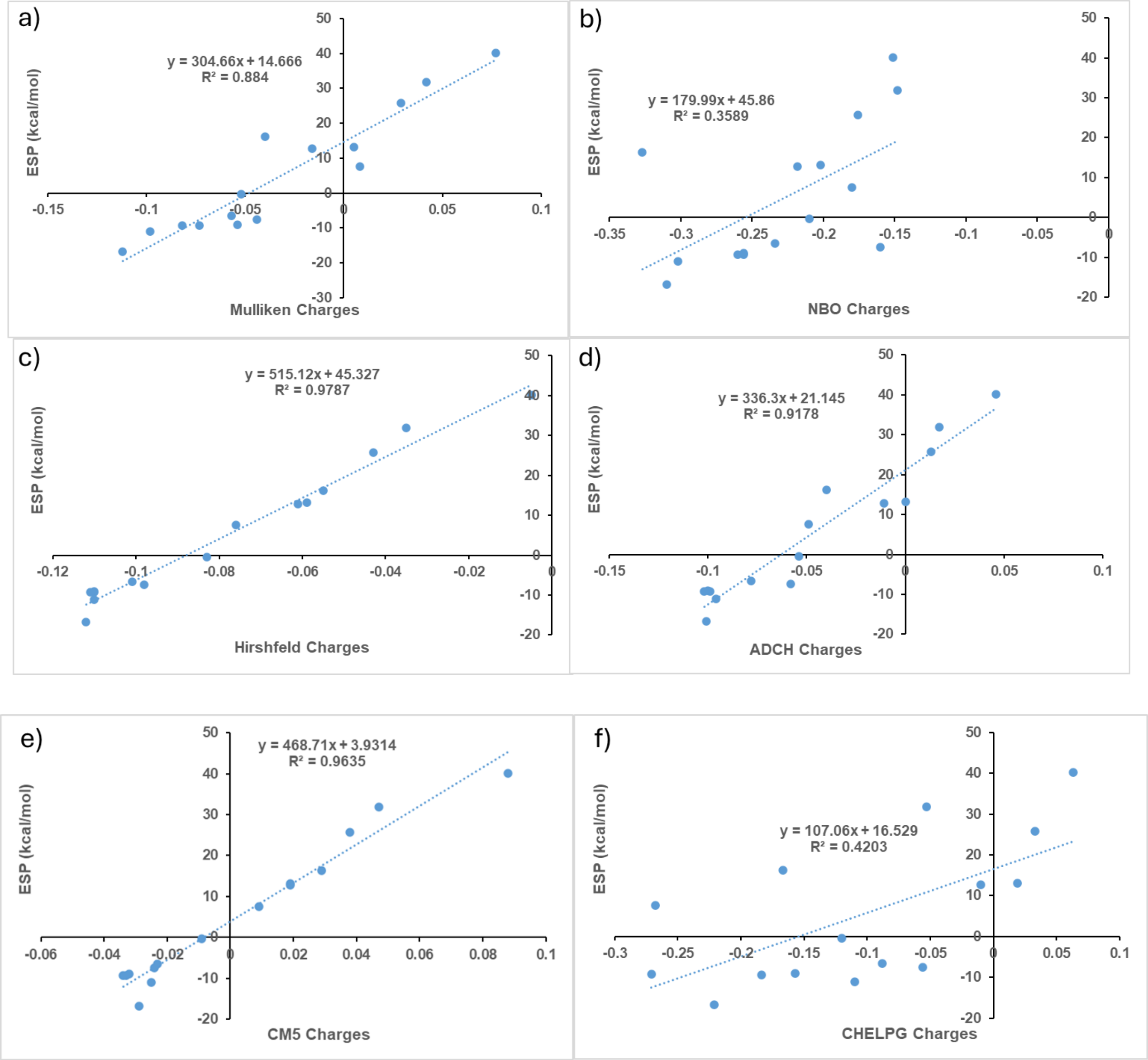

Figure S7. Correlation between all the charges and electrostatic potential (ESP, kcal mol^{-1}) evaluated at electron-density isosurface values of 0.001 a.u., computed at the DLPNO-CCSD(T)/def2-TZVP level of theory in the gas phase.

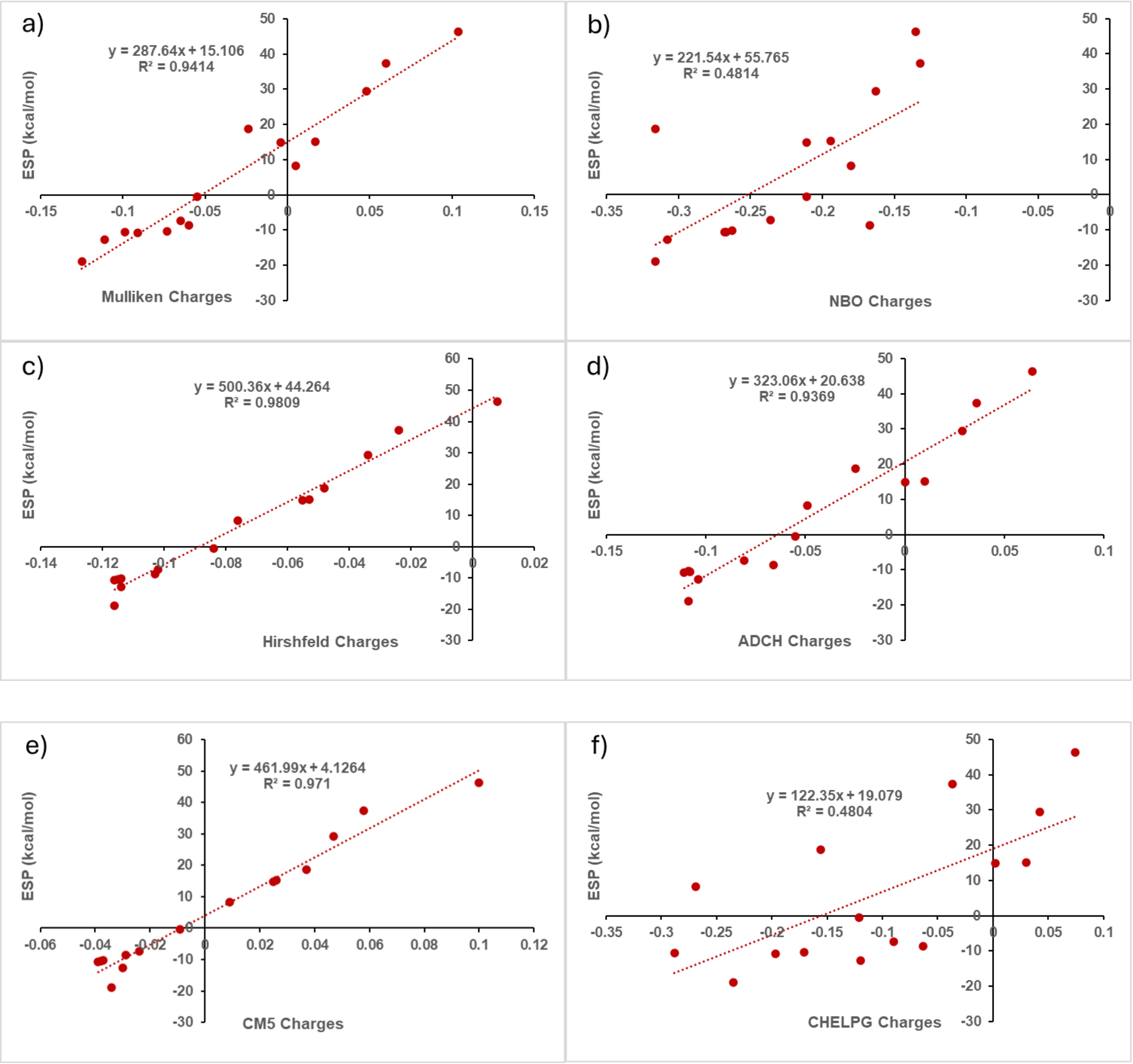

Figure S8. Correlation between all the charges and electrostatic potential (ESP, kcal mol^{-1}) evaluated at electron-density isosurface values of 0.001 a.u., computed at the DLPNO-CCSD(T)/def2-TZVP level of theory using the COSMO continuum solvation model in benzene medium.

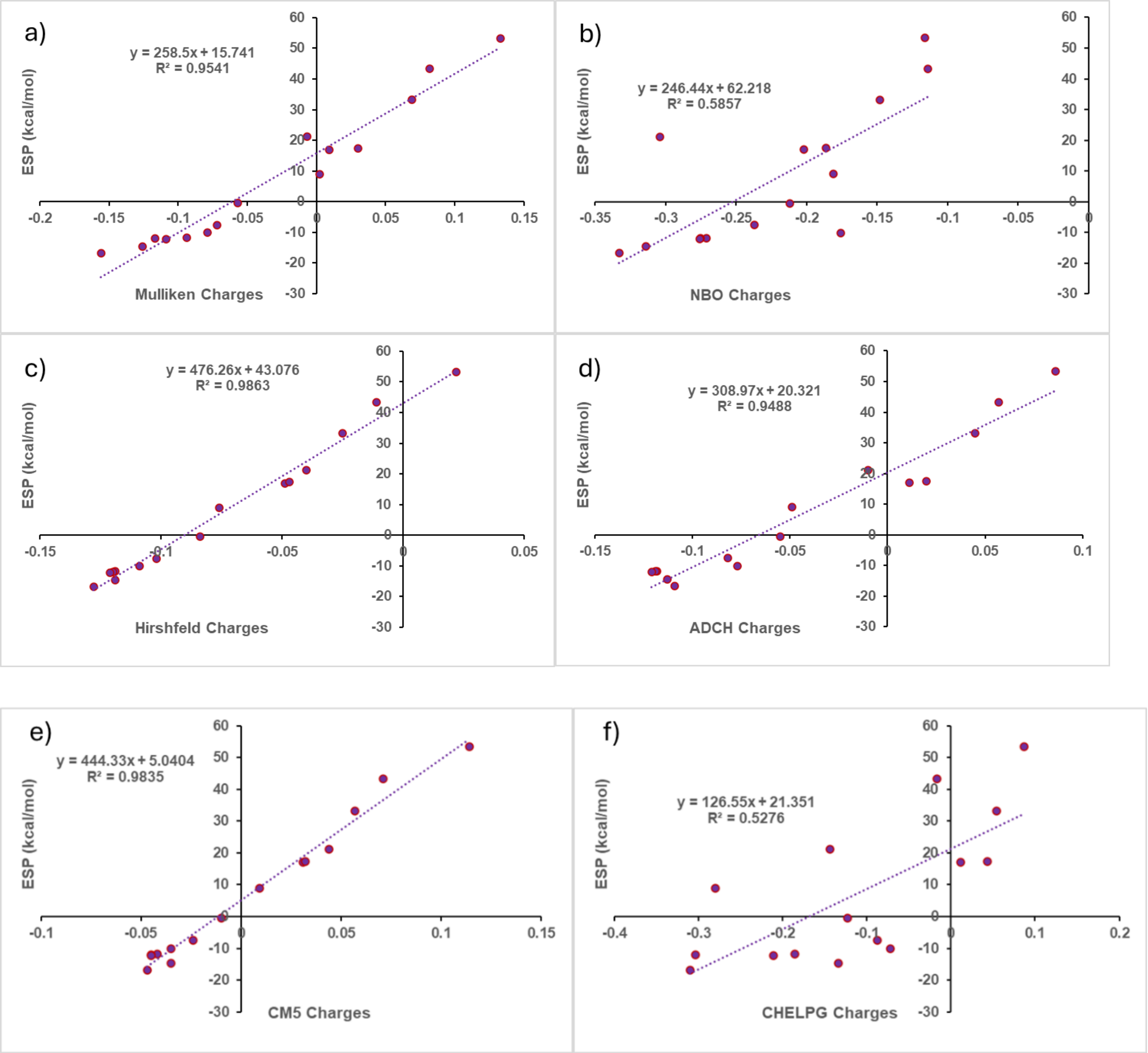

Figure S9. Correlation between all the charges and electrostatic potential (ESP, kcal mol^{-1}) evaluated at electron-density isosurface values of 0.001 a.u., computed at the DLPNO-CCSD(T)/def2-TZVP level of theory using the COSMO continuum solvation model in o-DCB medium.

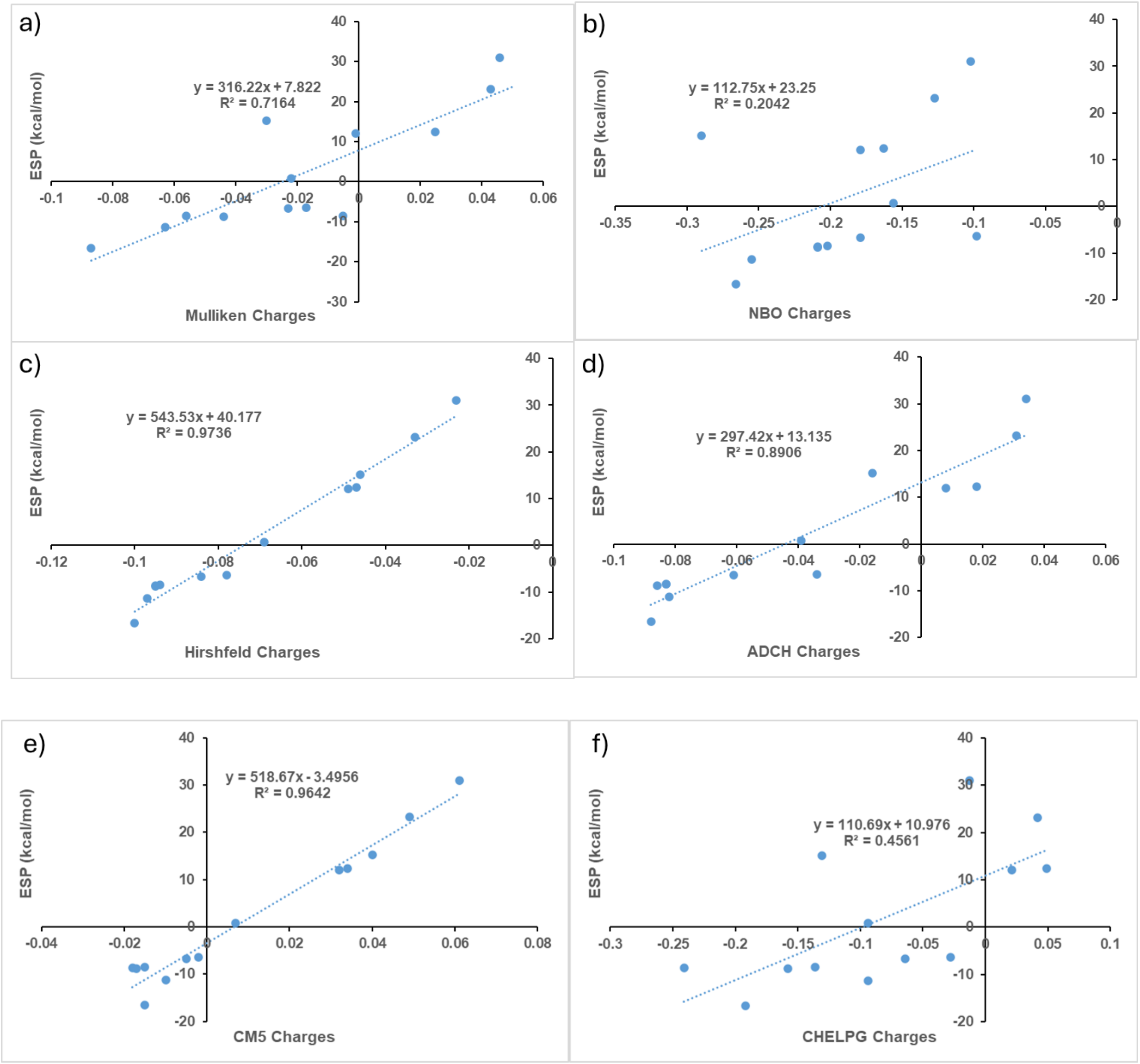

Figure S10. Correlation between all the charges and electrostatic potential (ESP, kcal mol^{-1}) evaluated at electron-density isosurface values of 0.001 a.u., computed at the ωB97M-V/def2-TZVPP level of theory in the gas phase.

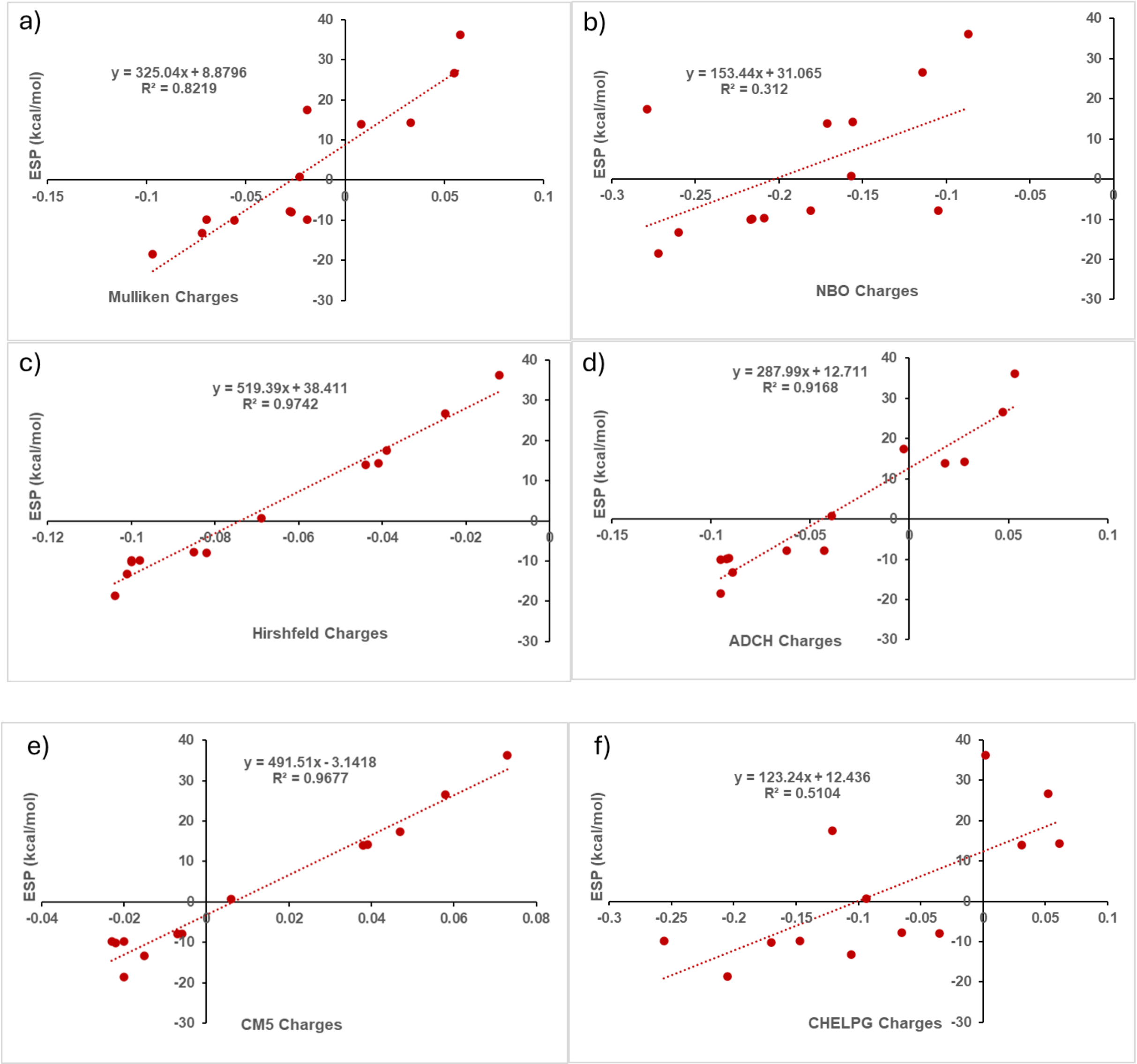

Figure S11. Correlation between all the charges and electrostatic potential (ESP, kcal mol^{-1}) evaluated at electron-density isosurface values of 0.001 a.u., computed at the ωB97M-V/def2-TZVPP level of theory using the COSMO continuum solvation model in benzene medium.

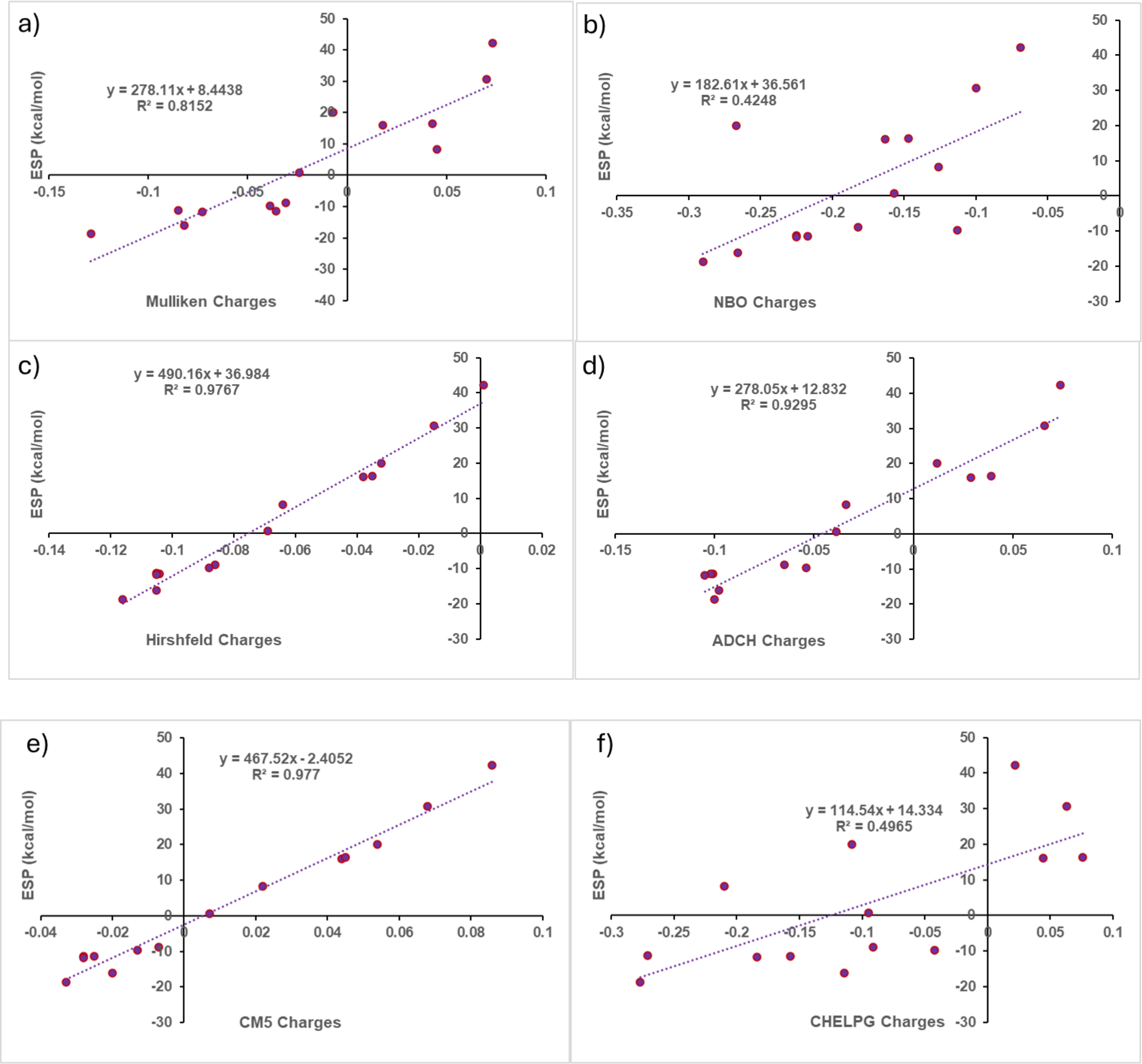

Figure S12. Correlation between all the charges and electrostatic potential (ESP, kcal mol^{-1}) evaluated at electron-density isosurface values of 0.001 a.u., computed at the ωB97M-V/def2-TZVPP level of theory using the COSMO continuum solvation model in o-DCB medium.

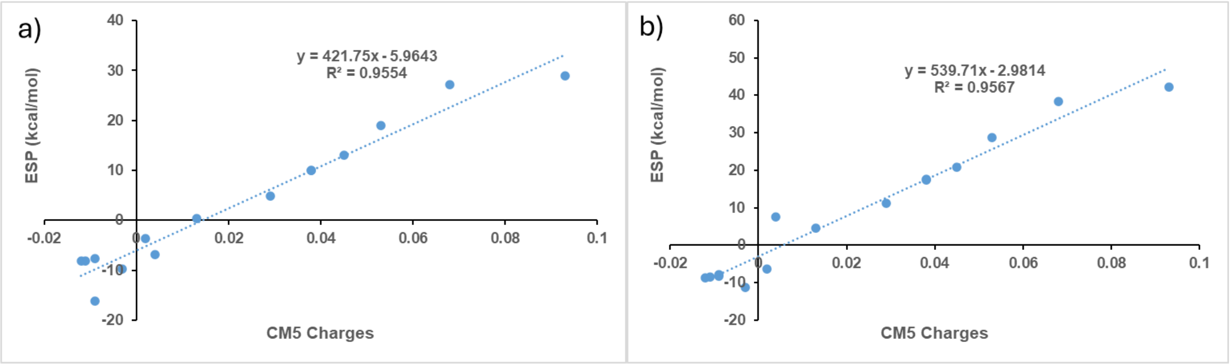

Figure S13. Correlation between CM5 charges and electrostatic potential (ESP, kcal mol^{-1}) evaluated at electron-density isosurface values of (a) 0.0005 a.u. and (b) 0.002 a.u., computed at the PBE0-D3/def2-TZVPP level of theory in the gas phase.

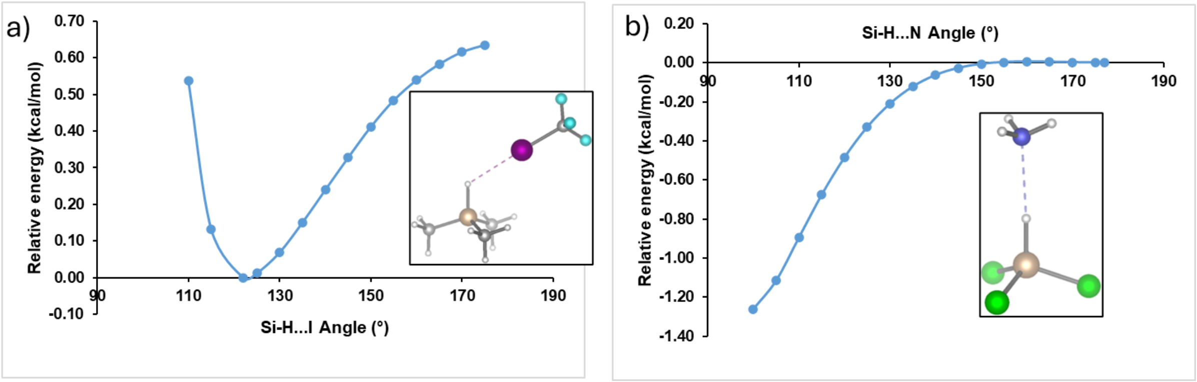

Figure S14. The angle scan of the optimized geometries of $Me_3SiH\ldots ICF_3$ and b) $SiCl_3H\ldots NH_3$ complexes. The optimized geometries are provided in the inset. [C: grey, N: blue, H: white, Si: golden, Cl: green, F:cyan]

Table S1 The calculated Mulliken, Natural Bond Orbital, Hirshfeld, ADCH, CM5 and AIM charges (in e) on the H and Si atoms of X_3Si-H in various media at the PBE0-D3/def2-TZVPP level of theory. The extrema of the ESP on the molecular surface (isosurface of 0.001) in the vicinity of H atom given in kcal/mol. The ESP evaluated at electron-density isosurface values of (a) 0.0005 a.u. and (b) 0.002 a.u. are given in parentheses.

X_3Si-H		Mulliken		NBO		Hirshfeld		ADCH		CM5		AIM		ESP (0.0005/ 0.002)
		H	Si	H	Si	H	Si	H	Si	H	Si	H	Si	
SiH_4	Gas	-0.047	0.187	-0.141	0.562	-0.067	0.267	-0.030	0.118	0.013	-0.052	-0.673	2.691	1.73 (0.38/4.61)
	BEN	-0.047	0.190	-0.141	0.563	-0.067	0.267	-0.030	0.119	0.013	-0.052	-0.673	2.691	1.71
	o-DCB	-0.048	0.192	-0.141	0.564	-0.067	0.267	-0.030	0.119	0.013	-0.052	-0.673	2.692	1.70
F_3SiH	Gas	-0.070	0.915	-0.277	2.185	-0.043	0.595	-0.013	0.541	0.045	0.438	-0.660	3.203	16.10 (13.11/ 20.91)

	BEN	-0.056	0.932	-0.263	2.183	-0.034	0.601	0.005	0.557	0.055	0.443	-0.650	3.201	19.17
	o-DCB	-0.045	0.943	-0.251	2.180	-0.027	0.605	0.019	0.568	0.062	0.447	-0.643	3.197	21.62
Cl_3SiH	Gas	-0.039	0.603	-0.165	1.183	-0.048	0.350	0.013	0.190	0.038	0.131	-0.642	2.899	12.88 (10.08/17.49)
	BEN	-0.027	0.615	-0.155	1.186	-0.041	0.356	0.026	0.199	0.046	0.137	-0.633	2.893	15.44
	o-DCB	-0.018	0.623	-0.146	1.188	-0.035	0.360	0.037	0.206	0.052	0.142	-0.627	2.892	17.55
Br_3SiH	Gas	-0.018	0.485	-0.148	0.882	-0.047	0.280	0.023	0.106	0.038	0.010	-0.636	2.674	12.92 (10.05/17.68)
	BEN	-0.007	0.499	-0.139	0.886	-0.040	0.285	0.036	0.112	0.045	0.016	-0.630	2.675	15.33
	o-DCB	0.001	0.509	-0.131	0.889	-0.034	0.289	0.046	0.117	0.051	0.021	-0.623	2.672	17.37
$(C_6F_5)_3SiH$	Gas	-0.013	0.353	-0.121	1.335	-0.059	0.323	-0.030	0.434	0.029	0.111	-0.643	2.849	7.18 (4.86/11.25)
	BEN	-0.016	0.344	-0.121	1.337	-0.059	0.327	-0.029	0.511	0.029	0.115	-0.643	2.849	7.90
	o-DCB	-0.018	0.339	-0.121	1.338	-0.059	0.329	-0.029	0.573	0.030	0.118	-0.643	2.852	8.57
$(CF_3)_3SiH$	Gas	-0.007	0.175	-0.123	0.939	-0.033	0.325	0.032	0.178	0.053	0.138	-0.632	2.744	22.82 (18.98/28.67)
	BEN	0.008	0.183	-0.108	0.943	-0.022	0.335	0.049	0.193	0.064	0.148	-0.621	2.745	27.04
	o-DCB	0.020	0.189	-0.096	0.947	-0.014	0.343	0.065	0.205	0.072	0.156	-0.612	2.747	30.52
$(CN)_3SiH$	Gas	-0.005	0.538	-0.093	1.169	-0.018	0.482	0.042	0.393	0.068	0.286	-0.619	2.949	31.77 (27.22/38.43)
	BEN	0.011	0.568	-0.073	1.174	-0.004	0.505	0.065	0.501	0.082	0.309	-0.605	2.947	38.55
	o-DCB	0.025	0.589	-0.056	1.177	0.008	0.523	0.085	0.613	0.095	0.328	-0.588	2.941	44.30
$(NO_2)_3SiH$	Gas	0.026	0.521	-0.112	1.430	-0.006	0.447	0.052	0.128	0.093	0.452	-0.602	2.913	34.40 (29.01/42.18)
	BEN	0.044	0.548	-0.093	1.437	0.010	0.473	0.074	0.054	0.108	0.477	-0.585	2.907	41.41
	o-DCB	0.060	0.571	-0.076	1.442	0.023	0.494	0.095	-0.126	0.120	0.498	-0.569	2.899	47.75
Ph_3SiH	Gas	-0.082	0.327	-0.173	1.384	-0.080	0.308	-0.054	0.597	0.002	0.090	-0.682	2.824	-5.34[a]/-7.02 (-5.77/-3.59[a], -6.32)
	BEN	-0.086	0.346	-0.175	1.387	-0.081	0.308	-0.057	0.805	0.000	0.090	-0.683	2.823	-6.28[a]/-8.12
	o-DCB	-0.088	0.357	-0.177	1.389	-0.082	0.307	-0.057	0.966	0.000	0.089	-0.684	2.823	-6.68
$(iPr)_3SiH$	Gas	-0.076	0.201	-0.196	1.428	-0.089	0.306	-0.077	0.216	-0.009	0.065	-0.690	2.761	-8.25 (-7.70/ -6.94[a], -8.22)
	BEN	-0.092	0.203	-0.205	1.433	-0.095	0.306	-0.087	0.218	-0.015	0.065	-0.695	2.761	-9.86
	o-DCB	-0.105	0.204	-0.212	1.438	-0.100	0.306	-0.096	0.219	-0.020	0.064	-0.699	2.761	-11.31
Me_3SiH	Gas	-0.100	0.382	-0.197	1.371	-0.092	0.325	-0.075	0.250	-0.012	0.080	-0.699	2.817	-8.13[a]/-8.65 (-8.01[a], -8.12/ -7.08[a], -8.59)
	BEN	-0.115	0.395	-0.207	1.378	-0.098	0.325	-0.087	0.255	-0.018	0.079	-0.704	2.818	-9.90[a]/-10.21
	o-DCB	-0.127	0.403	-0.215	1.384	-0.103	0.324	-0.097	0.259	-0.024	0.079	-0.708	2.818	-11.36[a]/-11.53
Et_3SiH	Gas	-0.092	0.328	-0.198	1.385	-0.091	0.312	-0.078	0.222	-0.011	0.068	-0.691	2.787	-8.27[a]/-8.60 (-8.04[a], -8.07/ -7.36[a], -8.57)
	BEN	-0.107	0.334	-0.208	1.391	-0.097	0.312	-0.089	0.225	-0.017	0.067	-0.696	2.787	-10.09[a]/-10.25

	o-DCB	-0.119	0.338	-0.216	1.395	-0.102	0.312	-0.099	0.227	-0.023	0.067	-0.700	2.787	-11.62[a]/-11.69
$(Me_3Si)_3SiH$	Gas	-0.081	-0.043	-0.093	-0.204	-0.077	0.021	-0.028	-0.091	0.004	-0.069	-0.669	0.416	-6.10[a]/-7.54 (-6.29[a], -6.86/ -4.57[a], -7.65)
	BEN	-0.062	-0.089	-0.102	-0.205	-0.083	0.018	-0.039	-0.093	-0.003	-0.072	-0.676	0.389	-8.00[a]/-9.18
	o-DCB	-0.099	-0.075	-0.109	-0.206	-0.088	0.016	-0.049	-0.093	-0.008	-0.074	-0.682	0.364	-9.80[a]/-10.77
$(NH_2)_3SiH$	Gas	-0.129	0.542	-0.255	1.810	-0.099	0.348	-0.082	0.420	-0.009	0.337	-0.707	3.080	-13.14 (-16.18/-7.75)
	BEN	-0.140	0.550	-0.263	1.809	-0.104	0.342	-0.092	0.444	-0.015	0.330	-0.711	3.076	-14.86
	o-DCB	-0.173	0.588	-0.282	1.825	-0.117	0.341	-0.096	0.482	-0.030	0.328	-0.720	3.069	-13.75
$(Me_2N)_3SiH$	Gas	-0.113	0.262	-0.252	1.899	-0.093	0.357	-0.076	0.292	-0.003	0.347	-0.701	3.062	-10.50 (-9.66/-11.18)
	BEN	-0.122	0.270	-0.259	1.902	-0.099	0.352	-0.086	0.297	-0.009	0.342	-0.707	3.063	-12.57
	o-DCB	-0.129	0.273	-0.265	1.905	-0.103	0.347	-0.094	0.300	-0.014	0.338	-0.711	3.065	-14.66

[a] The $V_{s,min}$ of H atom on the extension of Si-H.

Table S2 The calculated Mulliken, Natural Bond Orbital, Hirshfeld, ADCH, and CM5 charges (in e) on the H and Si atoms of X_3Si-H in various media at the M06-2X-D3/def2-TZVPP level of theory. The extrema of the ESP on the molecular surface (isosurface of 0.001) in the vicinity of H atom given in kcal/mol.

X_3Si-H		Mulliken		NBO		Hirshfeld		ADCH		CM5		ESP
		H	Si	H	Si	H	Si	H	Si	H	Si	
SiH_4	Gas	-0.049	0.196	-0.156	0.626	-0.070	0.281	-0.034	0.135	0.011	-0.045	1.16
	BEN	-0.050	0.202	-0.157	0.628	-0.070	0.281	-0.034	0.136	0.011	-0.045	1.13
	o-DCB	-0.052	0.205	-0.157	0.629	-0.071	0.282	-0.034	0.137	0.011	-0.044	1.11
F_3SiH	Gas	-0.045	0.922	-0.288	2.237	-0.045	0.618	-0.012	0.573	0.045	0.458	16.90
	BEN	-0.030	0.940	-0.273	2.234	-0.036	0.624	0.007	0.590	0.055	0.464	20.06
	o-DCB	-0.018	0.951	-0.262	2.231	-0.029	0.628	0.022	0.602	0.062	0.468	22.59
Cl_3SiH	Gas	-0.017	0.554	-0.177	1.238	-0.049	0.369	0.015	0.221	0.039	0.148	14.05
	BEN	-0.004	0.567	-0.166	1.242	-0.041	0.376	0.029	0.232	0.047	0.155	16.81
	o-DCB	0.005	0.576	-0.157	1.244	-0.035	0.381	0.041	0.240	0.054	0.160	19.11
Br_3SiH	Gas	0.005	0.502	-0.158	0.926	-0.048	0.290	0.028	0.130	0.039	0.018	14.17
	BEN	0.016	0.518	-0.149	0.931	-0.040	0.296	0.041	0.138	0.047	0.026	16.88
	o-DCB	0.026	0.529	-0.140	0.935	-0.034	0.301	0.052	0.145	0.053	0.031	19.17
$(C_6F_5)_3SiH$	Gas	0.018	0.389	-0.127	1.379	-0.057	0.343	-0.022	0.447	0.033	0.129	8.99
	BEN	0.016	0.373	-0.126	1.380	-0.057	0.348	-0.021	0.553	0.034	0.134	10.01
	o-DCB	0.014	0.362	-0.125	1.381	-0.056	0.351	-0.020	0.634	0.034	0.138	11.03
$(CF_3)_3SiH$	Gas	0.015	0.246	-0.129	1.004	-0.029	0.354	0.043	0.207	0.058	0.163	25.80
	BEN	0.034	0.263	-0.112	1.006	-0.018	0.364	0.061	0.223	0.070	0.174	30.51
	o-DCB	0.046	0.270	-0.098	1.008	-0.008	0.373	0.078	0.236	0.079	0.183	34.51
$(CN)_3SiH$	Gas	0.008	0.466	-0.103	1.221	-0.019	0.508	0.042	0.428	0.069	0.310	33.00

	BEN	0.023	0.492	-0.083	1.226	-0.004	0.532	0.067	0.548	0.084	0.335	39.98
	o-DCB	0.037	0.510	-0.066	1.229	0.008	0.550	0.087	0.667	0.096	0.354	45.91
$(NO_2)_3SiH$	Gas	0.065	0.599	-0.128	1.545	0.000	0.516	0.070	0.245	0.099	0.520	40.28
	BEN	0.084	0.624	-0.104	1.547	0.017	0.540	0.097	0.042	0.115	0.542	48.17
	o-DCB	0.100	0.645	-0.083	1.547	0.032	0.558	0.121	-0.228	0.130	0.560	55.03
Ph_3SiH	Gas	-0.045	0.362	-0.180	1.429	-0.080	0.323	-0.052	0.626	0.004	0.103	-4.32[a]/-6.23
	BEN	-0.048	0.380	-0.181	1.431	-0.080	0.323	-0.052	0.824	0.003	0.103	-5.05
	o-DCB	-0.050	0.395	-0.182	1.432	-0.081	0.322	-0.054	1.019	0.003	0.102	-5.81
$(iPr)_3SiH$	Gas	-0.023	0.344	-0.203	1.481	-0.089	0.320	-0.073	0.228	-0.007	0.075	-6.91[a]/-7.51
	BEN	-0.035	0.345	-0.210	1.485	-0.094	0.320	-0.082	0.230	-0.012	0.075	-8.93
	o-DCB	-0.046	0.346	-0.217	1.489	-0.098	0.320	-0.090	0.231	-0.017	0.075	-10.18
Me_3SiH	Gas	-0.076	0.409	-0.203	1.409	-0.091	0.341	-0.072	0.266	-0.009	0.093	-6.99[a]/-7.45
	BEN	-0.089	0.423	-0.211	1.415	-0.097	0.341	-0.082	0.272	-0.015	0.093	-8.49[a]/-8.76
	o-DCB	-0.099	0.432	-0.218	1.420	-0.101	0.341	-0.090	0.276	-0.019	0.093	-9.69[a]/-9.86
Et_3SiH	Gas	-0.050	0.410	-0.209	1.435	-0.091	0.326	-0.076	0.234	-0.009	0.078	-7.29[a]/-7.66
	BEN	-0.063	0.415	-0.212	1.435	-0.096	0.326	-0.086	0.237	-0.015	0.078	-8.87[a]/-9.08
	o-DCB	-0.073	0.418	-0.218	1.439	-0.100	0.326	-0.095	0.239	-0.019	0.078	-10.21[a]/-10.32
$(Me_3Si)_3SiH$	Gas	-0.008	-0.057	-0.100	-0.182	-0.078	0.028	-0.026	-0.087	0.005	-0.064	-5.17 [a]/-6.73
	BEN	-0.015	-0.075	-0.108	-0.184	-0.083	0.025	-0.037	-0.088	-0.001	-0.067	-6.89 [a]/-8.20
	o-DCB	-0.022	-0.090	-0.114	-0.187	-0.088	0.023	-0.046	-0.089	-0.006	-0.069	-8.49 [a]/ -9.60
$(NH_2)_3SiH$	Gas	-0.103	0.598	-0.263	1.862	-0.099	0.366	-0.077	0.457	-0.007	0.354	-16.51
	BEN	-0.112	0.604	-0.269	1.872	-0.103	0.360	-0.085	0.482	-0.011	0.346	-19.31
	o-DCB	-0.142	0.631	-0.286	1.885	-0.114	0.358	-0.086	0.520	-0.025	0.344	-16.55
$(Me_2N)_3SiH$	Gas	-0.084	0.335	-0.263	1.963	-0.095	0.371	-0.074	0.314	-0.003	0.360	-12.01
	BEN	-0.090	0.347	-0.269	1.966	-0.099	0.365	-0.082	0.322	-0.007	0.355	-15.25
	o-DCB	-0.096	0.355	-0.274	1.968	-0.103	0.361	-0.088	0.328	-0.011	0.351	-17.55

[a] The $V_{s,min}$ of H atom on the extension of Si-H.

Table S3 The calculated Mulliken, Natural Bond Orbital, Hirshfeld, ADCH, CM5 and CHELPG charges (in e) on the H and Si atoms of X_3Si-H in various mediums at the DLPNO-CCSD(T)/def2-TZVP level of theory. The extrema of the ESP on the molecular surface (isosurface of 0.001) in the vicinity of H atom given in kcal/mol.

X_3Si-H		Mulliken		NBO		Hirshfeld		ADCH		CM5		CHELPG		ESP
		H	Si	H	Si	H	Si	H	Si	H	Si	H	Si	
SiH_4	Gas	-0.052	0.208	-0.210	0.842	-0.083	0.333	-0.054	0.216	-0.009	0.036	-0.120	0.477	-0.38
	BEN	-0.055	0.219	-0.211	0.846	-0.084	0.334	-0.055	0.218	-0.009	0.038	-0.121	0.483	-0.46
	o-DCB	-0.057	0.228	-0.212	0.850	-0.084	0.336	-0.055	0.220	-0.010	0.039	-0.123	0.489	-0.53
F_3SiH	Gas	-0.040	1.170	-0.327	2.385	-0.055	0.712	-0.040	0.704	0.029	0.585	-0.167	1.200	16.28
	BEN	-0.024	1.179	-0.316	2.383	-0.048	0.717	-0.025	0.716	0.037	0.589	-0.156	1.216	18.76

	o-DCB	-0.007	1.185	-0.304	2.379	-0.040	0.721	-0.010	0.728	0.044	0.593	-0.144	1.228	21.23
Cl_3SiH	Gas	-0.016	0.804	-0.218	1.396	-0.061	0.428	-0.011	0.310	0.019	0.229	-0.010	0.353	12.76
	BEN	-0.004	0.812	-0.211	1.399	-0.055	0.434	0.000	0.320	0.025	0.235	0.002	0.366	14.84
	o-DCB	0.009	0.819	-0.202	1.402	-0.049	0.439	0.011	0.329	0.031	0.241	0.012	0.382	17.02
Br_3SiH	Gas	0.005	0.679	-0.202	1.110	-0.059	0.376	0.000	0.228	0.019	0.103	0.019	0.230	13.15
	BEN	0.017	0.689	-0.194	1.114	-0.053	0.382	0.010	0.237	0.026	0.110	0.030	0.240	15.18
	o-DCB	0.030	0.697	-0.186	1.118	-0.047	0.387	0.020	0.246	0.032	0.116	0.043	0.251	17.41
$(C_6F_5)_3SiH$	Gas	0.008	0.656	-0.180	1.534	-0.076	0.347	-0.049	0.287	0.009	0.181	-0.268	1.749	7.63
	BEN	0.005	0.650	-0.180	1.536	-0.076	0.351	-0.049	0.370	0.009	0.185	-0.269	1.726	8.24
	o-DCB	0.002	0.644	-0.181	1.538	-0.076	0.355	-0.049	0.460	0.009	0.189	-0.280	1.789	9.01
$(CF_3)_3SiH$	Gas	0.029	0.494	-0.176	1.187	-0.043	0.397	0.013	0.279	0.038	0.250	0.033	0.021	25.73
	BEN	0.048	0.493	-0.163	1.189	-0.034	0.406	0.029	0.292	0.047	0.259	0.042	0.052	29.34
	o-DCB	0.069	0.491	-0.148	1.191	-0.025	0.414	0.045	0.304	0.057	0.267	0.054	0.078	33.17
$(CN)_3SiH$	Gas	0.042	0.798	-0.148	1.397	-0.035	0.535	0.017	0.514	0.047	0.367	-0.053	0.605	31.86
	BEN	0.060	0.812	-0.132	1.399	-0.024	0.555	0.036	0.598	0.058	0.387	-0.037	0.623	37.30
	o-DCB	0.082	0.823	-0.114	1.400	-0.011	0.574	0.057	0.717	0.071	0.407	-0.017	0.636	43.31
$(NO_2)_3SiH$	Gas	0.077	0.802	-0.151	1.728	-0.005	0.588	0.046	0.265	0.088	0.597	0.063	0.337	40.12
	BEN	0.104	0.810	-0.135	1.730	0.008	0.611	0.064	0.216	0.100	0.619	0.074	0.383	46.38
	o-DCB	0.133	0.818	-0.116	1.731	0.022	0.633	0.086	0.048	0.114	0.640	0.087	0.430	53.33
Ph_3SiH	Gas	-0.057	0.516	-0.234	1.586	-0.101	0.322	-0.078	0.965	-0.023	0.151	-0.088	-0.001	-6.58
	BEN	-0.065	0.535	-0.236	1.589	-0.102	0.323	-0.081	1.142	-0.024	0.151	-0.090	-0.001	-7.25
	o-DCB	-0.072	0.549	-0.237	1.592	-0.102	0.322	-0.082	1.346	-0.024	0.151	-0.087	-0.008	-7.58
$(iPr)_3SiH$	Gas	-0.054	0.318	-0.256	1.618	-0.110	0.326	-0.100	0.283	-0.032	0.132	-0.157	0.033	-8.99
	BEN	-0.073	0.327	-0.263	1.623	-0.114	0.327	-0.109	0.286	-0.037	0.133	-0.171	0.039	-10.29
	o-DCB	-0.094	0.336	-0.271	1.629	-0.119	0.327	-0.118	0.288	-0.042	0.133	-0.185	0.044	-11.77
Me_3SiH	Gas	-0.082	0.557	-0.256	1.563	-0.110	0.357	-0.099	0.331	-0.033	0.153	-0.271	1.058	-9.12[a]/ -9.22
	BEN	-0.099	0.575	-0.267	1.570	-0.115	0.357	-0.108	0.337	-0.038	0.153	-0.288	1.099	-10.50[a]/ -10.53
	o-DCB	-0.117	0.593	-0.275	1.577	-0.120	0.358	-0.119	0.343	-0.045	0.155	-0.303	1.133	-11.95
Et_3SiH	Gas	-0.073	0.466	-0.260	1.578	-0.111	0.338	-0.102	0.295	-0.034	0.139	-0.184	0.509	-9.29[a]/ -9.31
	BEN	-0.091	0.478	-0.268	1.584	-0.116	0.339	-0.111	0.299	-0.039	0.139	-0.197	0.533	-10.68
	o-DCB	-0.109	0.491	-0.276	1.590	-0.121	0.340	-0.121	0.303	-0.045	0.140	-0.211	0.557	-12.17
$(Me_3Si)_3SiH$	Gas	-0.044	-0.057	-0.160	-0.116	-0.098	0.037	-0.058	-0.054	-0.024	-0.034	-0.056	-0.631	-7.20[a]/ -7.47
	BEN	-0.060	-0.058	-0.167	-0.115	-0.103	0.037	-0.066	-0.053	-0.029	-0.034	-0.063	-0.659	-8.51[a]/ -8.65
	o-DCB	-0.079	-0.058	-0.176	-0.113	-0.109	0.036	-0.077	-0.052	-0.035	-0.034	-0.072	-0.696	-0.11[a]/ -10.16
$(NH_2)_3SiH$	Gas	-0.112	0.877	-0.310	2.010	-0.112	0.431	-0.101	0.555	-0.029	0.428	-0.221	1.215	-16.79

	BEN	-0.125	0.877	-0.316	2.010	-0.116	0.428	-0.109	0.577	-0.034	0.424	-0.235	1.252	-18.93
	o-DCB	-0.156	0.900	-0.333	2.024	-0.128	0.428	-0.109	0.615	-0.047	0.424	-0.310	1.356	-16.79
$(Me_2N)_3SiH$	Gas	-0.098	0.566	-0.302	2.080	-0.110	0.425	-0.096	0.387	-0.025	0.434	-0.110	0.193	-11.07
	BEN	-0.111	0.580	-0.308	2.084	-0.114	0.421	-0.104	0.394	-0.030	0.431	-0.120	0.212	-12.71
	o-DCB	-0.126	0.590	-0.314	2.088	-0.119	0.418	-0.113	0.400	-0.035	0.428	-0.134	0.242	-14.60

[a] The $V_{s,min}$ of H atom on the extension of Si-H.

Table S4 The calculated Mulliken, Natural Bond Orbital, Hirshfeld, ADCH, CM5 and CHELPG charges (in e) on the H and Si atoms of X_3Si-H in various mediums at the ωB97M-V/def2-TZVP level of theory. The extrema of the ESP on the molecular surface (isosurface of 0.001) in the vicinity of H atom given in kcal/mol.

X_3Si-H		Mulliken		NBO		Hirshfeld		ADCH		CM5		CHELPG		ESP
		H	Si	H	Si	H	Si	H	Si	H	Si	H	Si	
SiH_4	Gas	-0.022	0.086	-0.156	0.625	-0.069	0.275	-0.039	0.154	0.007	-0.028	-0.094	0.373	0.77
	BEN	-0.023	0.091	-0.157	0.627	-0.069	0.276	-0.039	0.156	0.006	-0.027	-0.094	0.376	0.74
	o-DCB	-0.024	0.096	-0.157	0.629	-0.069	0.276	-0.039	0.157	0.007	-0.027	-0.095	0.379	0.71
F_3SiH	Gas	-0.030	0.883	-0.290	2.218	-0.046	0.562	-0.016	0.551	0.040	0.435	-0.131	1.000	15.17
	BEN	-0.019	0.896	-0.279	2.216	-0.039	0.566	-0.003	0.563	0.047	0.440	-0.121	1.015	17.45
	o-DCB	-0.007	0.906	-0.267	2.213	-0.032	0.570	0.012	0.575	0.054	0.444	-0.108	1.025	19.98
Cl_3SiH	Gas	-0.001	0.499	-0.179	1.227	-0.049	0.344	0.008	0.213	0.032	0.144	0.021	0.216	12.03
	BEN	0.008	0.507	-0.171	1.229	-0.044	0.348	0.018	0.220	0.038	0.148	0.031	0.226	13.92
	o-DCB	0.018	0.515	-0.163	1.231	-0.038	0.353	0.029	0.227	0.044	0.153	0.044	0.233	16.09
Br_3SiH	Gas	0.025	0.371	-0.163	0.956	-0.047	0.300	0.018	0.137	0.034	0.023	0.049	0.102	12.37
	BEN	0.033	0.382	-0.156	0.959	-0.041	0.304	0.028	0.142	0.039	0.028	0.061	0.105	14.25
	o-DCB	0.043	0.392	-0.147	0.962	-0.035	0.309	0.039	0.148	0.045	0.033	0.076	0.108	16.44
$(C_6F_5)_3SiH$	Gas	0.051	0.359	-0.131	1.367	-0.062	0.283	-0.025	0.377	0.022	0.118	-0.188	1.302	
	BEN	0.053	0.348	-0.129	1.366	-0.061	0.286	-0.024	0.301	0.023	0.121	-0.195	1.370	
	o-DCB	0.045	0.339	-0.126	1.364	-0.064	0.289	-0.034	0.560	0.022	0.124	-0.210	1.407	8.28
$(CF_3)_3SiH$	Gas	0.043	0.178	-0.127	1.000	-0.033	0.290	0.031	0.198	0.049	0.142	0.042	0.035	23.18
	BEN	0.055	0.183	-0.114	1.002	-0.025	0.298	0.047	0.210	0.058	0.151	0.052	0.062	26.61
	o-DCB	0.070	0.189	-0.100	1.004	-0.015	0.308	0.066	0.223	0.068	0.160	0.063	0.100	30.73
$(CN)_3SiH$	Gas	0.046	0.478	-0.102	1.207	-0.023	0.471	0.034	0.435	0.061	0.302	-0.013	0.429	31.04
	BEN	0.058	0.498	-0.087	1.210	-0.012	0.489	0.053	0.522	0.073	0.321	0.002	0.451	36.20
	o-DCB	0.073	0.519	-0.069	1.213	0.001	0.509	0.074	0.640	0.086	0.340	0.022	0.467	42.26
Ph_3SiH	Gas	-0.023	0.366	-0.179	1.413	-0.084	0.272	-0.061	0.664	-0.005	0.100	-0.064	-0.076	-5.69[a]/-6.69
	BEN	-0.028	0.381	-0.181	1.416	-0.085	0.272	-0.062	0.822	-0.006	0.100	-0.065	-0.071	-6.31[a]/-7.82
	o-DCB	-0.031	0.395	-0.182	1.417	-0.086	0.271	-0.065	0.986	-0.007	0.099	-0.091	-0.063	-8.88
$(iPr)_3SiH$	Gas	-0.005	0.288	-0.202	1.469	-0.094	0.269	-0.083	0.228	-0.015	0.074	-0.136	-0.025	-8.50
	BEN	-0.019	0.291	-0.209	1.473	-0.098	0.269	-0.091	0.230	-0.020	0.074	-0.147	-0.023	-9.79
	o-DCB	-0.036	0.295	-0.217	1.479	-0.104	0.269	-0.101	0.231	-0.025	0.074	-0.157	-0.030	-11.38
Me_3SiH	Gas	-0.056	0.391	-0.209	1.405	-0.095	0.301	-0.083	0.270	-0.018	0.096	-0.241	0.937	-8.45[a]/-8.59
	BEN	-0.070	0.404	-0.216	1.411	-0.100	0.300	-0.092	0.274	-0.023	0.096	-0.256	0.974	-9.79[a]/-9.84
	o-DCB	-0.085	0.416	-0.225	1.417	-0.105	0.300	-0.102	0.279	-0.028	0.096	-0.271	1.006	-11.30
Et_3SiH	Gas	-0.044	0.376	-0.209	1.423	-0.095	0.281	-0.086	0.237	-0.017	0.081	-0.158	0.417	-8.72[a]/-8.79
	BEN	-0.056	0.382	-0.217	1.428	-0.100	0.281	-0.095	0.240	-0.022	0.081	-0.170	0.433	-10.11[a]/ -10.12
	o-DCB	-0.073	0.389	-0.225	1.433	-0.105	0.281	-0.105	0.242	-0.028	0.081	-0.184	0.454	-11.75
$(Me_3Si)_3SiH$	Gas	-0.017	-0.086	-0.098	-0.197	-0.078	0.004	-0.034	-0.086	-0.002	-0.068	-0.028	-0.635	-6.41
	BEN	-0.027	-0.097	-0.105	-0.198	-0.082	0.002	-0.043	-0.087	-0.007	-0.070	-0.035	-0.663	-7.90
	o-DCB	-0.039	-0.106	-0.113	-0.199	-0.088	-0.001	-0.054	-0.087	-0.013	-0.071	-0.042	-0.703	-9.70
$(NH_2)_3SiH$	Gas	-0.087	0.553	-0.266	1.834	-0.100	0.352	-0.088	0.447	-0.015	0.347	-0.192	1.046	-16.59
	BEN	-0.097	0.560	-0.272	1.833	-0.104	0.347	-0.095	0.467	-0.020	0.342	-0.205	1.083	-18.54
	o-DCB	-0.129	0.583	-0.290	1.849	-0.116	0.345	-0.100	0.508	-0.033	0.340	-0.277	1.201	-18.67

$(Me_2N)_3SiH$	Gas	-0.063	0.277	-0.255	1.921	-0.097	0.344	-0.082	0.310	-0.010	0.353	-0.094	0.138	-11.30
	BEN	-0.072	0.289	-0.260	1.923	-0.101	0.340	-0.089	0.316	-0.015	0.350	-0.106	0.183	-13.26
	o-DCB	-0.082	0.298	-0.266	1.927	-0.105	0.336	-0.098	0.323	-0.020	0.345	-0.114	0.191	-16.07

[a] The $V_{s,min}$ of H atom on the extension of Si-H.

Table S5 The calculated Mulliken, Natural Bond Orbital, Hirshfeld, ADCH and CM5 charges (in e) on the H and C atoms of X_3C-H in various media at the PBE0-D3/def2-TZVPP level of theory. The extrema of the ESP on the molecular surface in the vicinity of H atom given in kcal/mol.

X_3C-H	Medium	Mulliken		NBO		Hirshfeld		ADCH		CM5		ESP
		H	C	H	C	H	C	H	C	H	C	
$(CF_3)_3CH$	Gas	0.128	-0.171	0.277	-0.500	0.072	-0.065	0.151	-0.172	0.138	-0.100	40.57
	BEN	0.148	-0.154	0.294	-0.505	0.084	-0.062	0.171	-0.166	0.151	-0.096	46.29
	o-DCB	0.163	-0.142	0.308	-0.509	0.094	-0.059	0.186	-0.161	0.160	-0.093	50.77
$(CN)_3CH$	Gas	0.156	0.428	0.328	-0.493	0.113	0.077	0.204	0.125	0.175	0.040	57.39
	BEN	0.173	0.483	0.355	-0.502	0.131	0.087	0.238	0.140	0.193	0.050	66.91
	o-DCB	0.187	0.520	0.375	-0.508	0.145	0.095	0.266	0.152	0.207	0.058	74.36
$(NO_2)_3CH$	Gas	0.205	-0.019	0.272	0.016	0.089	0.097	0.181	-0.102	0.187	0.212	54.00
	BEN	0.220	0.011	0.293	0.019	0.105	0.108	0.212	-0.308	0.202	0.223	62.17
	o-DCB	0.232	0.032	0.309	0.021	0.118	0.117	0.235	-0.543	0.214	0.231	68.75
$(NH_2)_3CH$	Gas	0.077	0.153	0.138	0.243	0.021	0.078	0.073	0.229	0.107	0.175	8.63
	BEN	0.086	0.113	0.122	0.246	0.013	0.075	0.079	0.274	0.096	0.172	12.71
	o-DCB	0.093	0.110	0.129	0.246	0.019	0.075	0.093	0.305	0.103	0.171	15.69
$(Me_2N)_3CH$	Gas	0.055	0.097	0.155	0.282	0.021	0.091	0.060	0.156	0.114	0.204	8.79
	BEN	0.068	0.124	0.161	0.278	0.025	0.090	0.067	0.165	0.117	0.203	10.35
	o-DCB	0.076	0.099	0.163	0.280	0.026	0.090	0.070	0.179	0.119	0.202	11.30
$(Me_3Si)_3CH$	Gas	0.078	0.010	0.284	-1.663	0.040	-0.270	0.129	-0.346	0.112	-0.216	13.34
	BEN	0.083	0.005	0.288	-1.664	0.043	-0.269	0.133	-0.347	0.115	-0.216	14.93
	o-DCB	0.087	0.004	0.291	-1.666	0.045	-0.269	0.137	-0.347	0.117	-0.216	16.25

Table S6 The calculated Mulliken, Natural Bond Orbital, Hirshfeld, ADCH and CM5 charges (in e) on the H and Al atoms of X_2Al-H at the PBE0-D3/def2-TZVPP level of theory. The extrema of the ESP on the molecular surface in the vicinity of H atom given in kcal/mol.

X_2Al-H	Medium	Mulliken		NBO		Hirshfeld		ADCH		CM5		ESP
		H	Al	H	Al	H	Al	H	Al	H	Al	
$(CF_3)_2AlH$	Gas	-0.091	0.507	-0.369	1.298	-0.124	0.560	-0.083	0.442	-0.046	0.428	7.19

	BEN											
	o-DCB											
$(CN)_2AlH$	Gas	-0.093	0.695	-0.336	1.449	-0.116	0.645	-0.082	0.504	-0.036	0.504	12.26
	BEN	-0.140	0.847	-0.399	1.618	-0.132	0.773	-0.106	0.858	-0.055	0.641	14.25
	o-DCB	-0.182	1.004	-0.460	1.799	-0.148	0.917			-0.075	0.796	
$(NO_2)_2AlH$	Gas	-0.127	0.562	-0.369	1.535	-0.131	0.489	-0.097	0.083	-0.047	0.489	2.77
	BEN	-0.131	0.842	-0.448	1.946	-0.133	0.771	-0.097	0.716	-0.055	0.726	9.92
	o-DCB	-0.185	1.067	-0.509	2.088	-0.154	0.912	-0.463	1.671	-0.080	0.868	11.91
$(NH_2)_2AlH$	Gas	-0.148	0.513	-0.405	1.665	-0.155	0.461	-0.139	0.380	-0.077	0.463	-14.17
	BEN	-0.207	0.498	-0.455	1.791	-0.177	0.536	-0.178	0.465	-0.102	0.537	-16.05
	o-DCB	-0.281	0.663	-0.508	1.913	-0.201	0.622	-0.214	0.584	-0.131	0.623	-18.38
$(Me_2N)_2AlH$	Gas	-0.141	0.338	-0.399	1.660	-0.152	0.450	-0.144	0.359	-0.074	0.451	-13.34
	BEN	-0.207	0.498	-0.455	1.791	-0.177	0.536	-0.178	0.465	-0.102	0.537	-16.05
	o-DCB	-0.281	0.663	-0.508	1.913	-0.201	0.622	-0.214	0.585	-0.131	0.623	-18.3
$(Me_3Si)_2AlH$	Gas	-0.131	0.301	-0.389	0.797	-0.168	0.297	-0.130	0.211	-0.097	0.229	-15.33
	BEN											
	o-DCB											

Table S7 The calculated Mulliken, Natural Bond Orbital, Hirshfeld, ADCH and CM5 charges (in e) on the H and P atoms of X_2P-H at the PBE0-D3/def2-TZVPP level of theory. The extrema of the ESP on the molecular surface in the vicinity of H atom given in kcal/mol.

X_2P-H	Medium	Mulliken		NBO		Hirshfeld		ADCH		CM5		ESP
		H	P	H	P	H	P	H	P	H	P	
$(CF_3)_2PH$	Gas	0.056	0.049	0.009	0.373	0.015	0.106	0.095	-0.023	0.101	-0.035	25.59
	BEN	0.070	0.060	0.025	0.380	0.026	0.117	0.118	-0.015	0.113	-0.025	30.02
	o-DCB	0.081	0.068	0.03	0.386	0.035	0.126	0.137	-0.008	0.122	-0.016	33.69
$(CN)_2PH$	Gas	0.080	0.248	0.042	0.571	0.038	0.259	0.110	0.209	0.123	0.111	35.67
	BEN	0.096	0.284	0.062	0.586	0.053	0.286	0.135	0.254	0.139	0.139	42.73
	o-DCB	0.109	0.315	0.079	0.600	0.066	0.309	0.156	0.289	0.151	0.162	48.68
$(NO_2)_2PH$	Gas	0.114	0.229	0.009	0.668	0.028	0.252	0.095	0.151	0.129	0.229	34.01
	BEN	0.130	0.263	0.024	0.700	0.041	0.285	0.113	0.191	0.142	0.261	39.87
	o-DCB	0.142	0.291	0.037	0.726	0.052	0.311	0.128	0.225	0.153	0.288	44.78
$(NH_2)_2PH$	Gas	0.004	0.170	-0.086	0.882	-0.025	0.096	0.080	-0.083	0.070	0.065	1.63
	BEN	0.003	0.145	-0.089	0.861	-0.029	0.075	0.092	-0.126	0.065	0.045	2.95
	o-DCB	0.009	0.130	-0.085	0.843	-0.027	0.061	0.105	-0.163	0.066	0.030	3.45
$(Me_2N)_2PH$	Gas	-0.025	0.135	-0.105	0.916	-0.034	0.116	0.059	-0.093	0.057	0.088	0.94
	BEN	-0.017	0.113	-0.098	0.900	-0.031	0.101	0.074	-0.134	0.061	0.073	1.57
	o-DCB	-0.009	0.095	-0.092	0.887	-0.028	0.089	0.087	-0.167	0.064	0.060	2.26
$(Me_3Si)_2PH$	Gas	0.023	-0.252	0.033	-0.445	-0.008	-0.134	0.058	-0.245	0.078	-0.216	6.17
	BEN	0.023	-0.254	0.033	-0.449	-0.008	-0.137	0.073	-0.307	0.077	-0.219	6.58
	o-DCB	0.041	-0.362	0.044	-0.506	-0.005	-0.176	0.085	-0.362	0.081	-0.258	6.81

Table S8 The calculated Mulliken, Natural Bond Orbital, Hirshfeld, ADCH and CM5 charges (in e) on the H and Ge atoms of X_3Ge-H in the gas phase at the PBE0-D3/def2-TZVPP level of theory. The extrema of the ESP on the molecular surface in the vicinity of H atom given in kcal/mol.

X_3Ge-H	Mulliken		NBO		Hirshfeld		ADCH		CM5		ESP
	H	Ge	H	Ge	H	Ge	H	Ge	H	Ge	
GeH_4	-0.018	0.071	-0.113	0.452	-0.063	0.251	-0.029	0.115	-0.004	0.014	2.07
F_3GeH	0.006	1.003	-0.214	2.174	-0.002	0.691	0.040	0.667	0.062	0.656	28.33
Cl_3GeH	0.040	0.739	-0.131	1.221	-0.021	0.430	0.042	0.286	0.042	0.329	20.50
Br_3GeH	0.049	0.673	-0.118	0.914	-0.024	0.347	0.046	0.176	0.038	0.196	18.69
$(C_6F_5)_3Ge$-H	0.056	0.405	-0.096	1.279	-0.045	0.355	-0.013	0.460	0.021	0.257	10.72
$(CF_3)_3GeH$	0.069	0.089	-0.104	0.888	-0.026	0.327	0.034	0.185	0.038	0.244	23.14
$(CN)_3GeH$	0.102	0.350	-0.058	1.128	-0.002	0.523	0.055	0.348	0.062	0.433	36.19
$(NO_2)_3GeH$	0.082	0.628	-0.108	1.301	0.010	0.510	0.070	0.271	0.079	0.602	38.69
Ph_3GeH	-0.020	0.365	-0.147	1.325	-0.072	0.328	-0.050	0.565	-0.012	0.221	-3.38[a]/ -5.25
$(iPr)_3GeH$	-0.047	0.229	-0.178	1.335	-0.084	0.321	-0.075	0.246	-0.025	0.196	-7.11[a]/ -7.99
Me_3GeH	-0.053	0.329	-0.178	1.327	-0.087	0.344	-0.076	0.280	-0.028	0.211	-6.60[a]/ -7.20
Et_3GeH	-0.052	0.265	-0.181	1.326	-0.087	0.329	-0.081	0.253	-0.028	0.199	-7.29[a]/ -7.71
$(Me_3Ge)_3GeH$	-0.032	-0.145	-0.079	-0.205	-0.076	0.024	-0.026	-0.099	-0.016	-0.040	-5.12[a]/ -6.80
$(NH_2)_3GeH$	-0.057	0.529	-0.217	1.758	-0.077	0.423	-0.056	0.502	-0.012	0.513	
$(Me_2N)_3GeH$	-0.038	0.330	-0.206	1.828	-0.070	0.432	-0.050	0.427	-0.004	0.536	

[a] The $V_{s,min}$ of H atom on the extension of Ge-H.

Table S9 The calculated Mulliken, Natural Bond Orbital, Hirshfeld, ADCH and CM5 charges (in e) on the H and Sn atoms of X_3Sn-H in the gas phase at the PBE0-D3/def2-TZVPP level of theory. The extrema of the ESP on the molecular surface in the vicinity of H atom given in kcal/mol.

X_3Sn-H	Mulliken		NBO		Hirshfeld		ADCH		CM5		ESP
	H	Sn	H	Sn	H	Sn	H	Sn	H	Sn	
SnH_4	-0.118	0.470	-0.166	0.662	-0.079	0.314	-0.053	0.211	-0.032	0.127	-0.024[a]/ -0.829
F_3SnH	-0.066	1.426	-0.240	2.318	-0.001	0.823	0.037	0.814	0.050	0.867	31.14
Cl_3SnH	-0.072	1.054	-0.161	1.430	-0.023	0.533	0.041	0.403	0.026	0.504	22.10
Br_3SnH	-0.069	0.938	-0.148	1.149	-0.029	0.436	0.045	0.275	0.019	0.356	19.28
$(C_6F_5)_3SnH$	-0.064	0.931	-0.144	1.414	-0.053	0.439	-0.021	0.381	-0.002	0.417	10.54
$(CF_3)_3SnH$	-0.066	0.421	-0.157	0.985	-0.041	0.412	0.012	0.277	0.008	0.398	19.69
$(CN)_3SnH$	-0.045	1.071	-0.109	1.336	-0.014	0.639	0.036	0.419	0.037	0.620	34.66
$(NO_2)_3SnH$	-0.031	0.921	-0.165	1.391	-0.006	0.600	0.044	0.507	0.045	0.754	34.29
Ph_3SnH	-0.132	0.973	-0.197	1.427	-0.086	0.395	-0.069	0.579	-0.039	0.361	-4.71[a]/ -5.51
$(iPr)_3SnH$	-0.146	0.698	-0.224	1.361	-0.102	0.376	-0.105	0.307	-0.056	0.324	-9.24[a]/ -9.54
Me_3SnH	-0.160	0.851	-0.224	1.347	-0.102	0.410	-0.104	0.352	-0.056	0.347	-8.23[a]/ -8.49

Et_3SnH	-0.160	0.786	-0.229	1.338	-0.104	0.389	-0.112	0.320	-0.058	0.331	-9.37[a]/ -9.54
$(Me_3Sn)_3SnH$	-0.118	-0.149	-0.120	-0.179	-0.090	0.038	-0.041	-0.096	-0.044	-0.009	-6.64[a]/ -7.57
$(NH_2)_3SnH$	-0.155	1.042	-0.255	1.837	-0.086	0.513	-0.071	0.568	-0.038	0.690	1.20[a]/ 1.11
$(Me_2N)_3SnH$	-0.125	0.995	-0.236	1.920	-0.073	0.521	-0.051	0.519	-0.023	0.718	0.05

[a] The $V_{s,min}$ of H atom on the extension of Sn-H.

Table S10 The calculated Mulliken, Natural Bond Orbital, Hirshfeld, ADCH and CM5 charges (in e) on the H and Pb atoms of X_3Pb-H in the gas phase at the PBE0-D3/def2-TZVPP level of theory. The extrema of the ESP on the molecular surface in the vicinity of H atom given in kcal/mol.

X_3Pb-H	Mulliken		NBO		Hirshfeld		ADCH		CM5		ESP
	H	Pb	H	Pb	H	Pb	H	Pb	H	Pb	
PbH_4	-0.109	0.434	-0.124	0.498	-0.062	0.245	-0.045	0.181	-0.026	0.106	0.70[a]/ -0.25
F_3PbH	-0.016	1.371	-0.138	2.142	0.058	0.794	0.100	0.814	0.097	0.879	42.78
Cl_3PbH	-0.038	1.016	-0.085	1.313	0.022	0.520	0.088	0.421	0.059	0.542	30.08
Br_3PbH	-0.044	0.914	-0.083	1.052	0.010	0.424	0.086	0.287	0.045	0.401	25.71
$(C_6F_5)_3PbH$	-0.066	0.871	-0.102	1.260	-0.027	0.397	-0.007	0.291	0.010	0.431	14.88
$(CF_3)_3PbH$	-0.062	0.298	-0.119	0.813	-0.023	0.351	0.021	0.246	0.013	0.383	19.13
$(CN)_3PbH$	-0.012	0.943	-0.044	1.198	0.026	0.601	0.072	0.388	0.065	0.634	41.21
$(NO_2)_3PbH$	-0.050	0.840	-0.151	1.128	-0.005	0.519	0.035	0.452	0.030	0.673	27.94
Ph_3PbH	-0.123	0.821	-0.159	1.248	-0.067	0.339	-0.061	0.560	-0.032	0.359	-3.51[a]/ -4.36
$(iPr)_3PbH$	-0.155	0.611	-0.203	1.153	-0.095	0.313	-0.115	0.292	-0.062	0.315	-10.91[a]/ -11.11
Me_3PbH	-0.154	0.753	-0.191	1.151	-0.087	0.351	-0.103	0.338	-0.054	0.341	-8.04[a]/ -8.30
Et_3PbH	-0.164	0.695	-0.204	1.135	-0.094	0.328	-0.120	0.310	-0.061	0.324	-10.46[a]/ -10.58
$(Me_3Pb)_3PbH$											
$(NH_2)_3PbH$	-0.130	0.930	-0.197	1.611	-0.054	0.465	-0.047	0.543	-0.018	0.667	6.24
$(Me_2N)_3PbH$	-0.109	0.892	-0.185	1.698	-0.045	0.472	-0.034	0.534	-0.008	0.693	3.49

[a] The $V_{s,min}$ of H atom on the extension of Pb-H.

Materials

Tris(pentafluorophenyl)silane (TPFPS), triphenylsilane (TPS, 97%), triethylsilane (TES, 99 %), tetramethylsilane (TMS, >99%), benzene-D_6 (99.6 atom% D), cyclohexane-D_{12} (>99.6 atom% D), and chloroform-D ($CDCl_3$, 99.8 atom% D) were purchased from Sigma Aldrich (Merck). 1,1,1,3,3,3-Hexamethyl-2-(trimethylsilyl)trisilane (tris(trimethylsilyl)silane, TTMSS, 95% (stabilized with 0.05 % TBBPA)) was purchased from BLDpharm. 1,2-Dichlorobenzene-D_4 (o-DCB-D_4, 98 atom% D) was purchased from Fisher Scientific. All the materials were used directly without additional purification.

Sample preparation

With the exception of tris(pentafluorophenyl)silane, 50 μmol of the silane of interest was dissolved in 700 μL of deuterated solvent and placed into the 5mm NMR tube (Wilmad®, >400 MHz) and capped. Tris(pentafluorophenyl)silane was poorly soluble in all the tested media; therefore, the tests were conducted with 4 μmol (2 mg) of the compound, resulting in only partial dissolution in all of the tested solvents. A small amount of TMS was added for referencing.

NMR measurements and data processing

NMR measurements were performed using a JEOL spectrometer JNM-ECZ400R with a superconducting coil having a magnetic field of 9.4 T (working frequency: 399.8 MHz for ^{1}H) equipped with a 5 mm liquid probe. Proton spectra were measured at 25 °C using a simple single-pulse job for proton NMR measurement with a 45° pulse angle and a relaxation delay of 5 s, with the sample tube spinning at 15 Hz. Variable temperature (VT) proton NMR spectra were recorded in CD_2Cl_2 solvent at temperatures ranging from -65 °C to 25 °C, and in benzene-D_6 solvent at temperatures ranging from 15 °C to 65 °C. Magnetic shim coils were adjusted automatically or manually along the Z1, Z2, Z3, and Z4 axes. After the measurements, the acquired spectra were processed and visualized using MestReNova software. First, zero- and first-order phase corrections were applied to the acquired spectra. Spectral referencing with respect to tetramethylsilane (0 ppm) or solvent reference shifts (benzene-D_5 proton signal in benzene-D_6 to 7.16 ppm, H4 proton signal of *o*-DCB to 6.94 ppm, cyclohexane-D_{11} proton signal in cyclohexane-D_{12} to 1.38 ppm, and $CHCl_3$ proton signal in $CDCl_3$ to 7.26 ppm) was done only for chemical shift comparison in different solvents at 25 °C (see Table S11). Referencing was not applied to the VT NMR trends due to the varying temperature drift of reference peaks. The Si-H proton signal was recognized according to the presence of a doublet signal caused by ^{1}J-coupling of ^{29}Si-^{1}H nuclei with $^{1}J_{Si\text{-}H}$ constant ranging from 150 to 260 Hz, appearing as intense sidebands along the main ^{28}Si-^{1}H signal (singlet or heptet depending on the presence of other interacting ^{1}H or ^{19}F nuclei). The chemical shift positions and $^{1}J_{Si\text{-}H}$ constants for all the tested silanes in various solvents are summarized in Table S11. The individual spectra are presented in Figures S15-S34.

To determine the ^{29}Si chemical shift position of the inspected Si-H environment, an approximate result was first obtained by recording a gradient-enhanced ^{1}H-^{29}Si HMQC spectrum with 256 y-points (corresponding to the ^{29}Si chemical shift). The Si-H environment

was recognized by observing two x-correlation points at a single y-value separated by $^{1}J_{Si\text{-}H}$ splitting constant value. Then, a ^{29}Si single-pulse ^{1}H-decoupled spectrum with ^{1}H NOE enhancement was recorded to get a more precise ^{29}Si chemical shift value. In the case of TPFPS, due to its limited solubility, only HMQC spectra were successfully recorded. The results are summarized in Table S14. The individual spectra are presented in Figures S35-S47.

Finally, ^{1}H spectra of neat liquid silanes with a small addition of TMS for referencing have been recorded using a simple ^{1}H single-pulse job without an active lock channel. The results are presented in Figures S48-S49.

Table S11. Proton chemical shifts and ^{1}J ^{29}Si-^{1}H coupling constants of the tested silanes in varying solvents. In parenthesis, the calculated proton chemical shifts are given at the PBE0-D3/def2-TZVPP level of theory using the COSMO continuum solvation model.

solvent	silane							
	**TPFPS**		**TTMSS**		**TES**		**TPS**	
	δ (ppm)	$^{1}J_{Si\text{-}H}$ (Hz)	δ (ppm)	$^{1}J_{Si\text{-}H}$ (Hz)	δ (ppm)	$^{1}J_{Si\text{-}H}$ (Hz)	δ (ppm)	$^{1}J_{Si\text{-}H}$ (Hz)
Neat			2.29	155.0	3.70	179.5		
Benz.-D_6	5.81 (6.609)	252.0	2.54 (2.636)	153.9	3.90 (4.016)	177.5	5.71 (6.199)	197.9
oDCB-D_4	5.95 (6.611)	252.4	2.31 (2.513)	154.2	3.69 (3.927)	177.5	5.50 (6.151)	198.4
CHX-D12	5.91 (6.613)	251.1	2.27 (2.652)	155.6	3.69 (4.031)	179.6	5.45 (6.205)	198.4
$CDCl_3$	5.90 (6.608)	252.5	2.14 (2.554)	153.4	3.60 (3.960)	176.5	5.49 (6.181)	199.4
CD_2Cl_2	5.91 (6.608)	253.0	2.16 (2.515)	153.1	3.60 (3.931)	176.3	5.45 (6.167)	199.3

Relation of the measured chemical shifts to the DFT-calculated ESPs

Simply translated, the DFT-calculated ESPs on the protons of silanes with EWG groups are positive. Upon transition from a non-polar to a polar environment, the ESP increases, which translates into a downfield chemical shift change. On the other hand, EDG substituents produce negative ESPs and upfield shifts. However, in the experimental data (Table S11), such trends are not strictly followed. In the case of tris(pentafluorophenyl)silane (TPFPS), upon the transition from benzene-D_6 to oDCB-D_4 solvents, the trend is clearly followed, but little to no changes in chemical shifts are observed for non-aromatic solvents. Similar to TPFPS, EDG silanes follow this trend in aromatic solvents as well, but not strictly in non-aromatic solvents. Only TTMSS and TES follow all the trends well, while the trend for TPS is slightly opposite in non-aromatic solvents.

On the other hand, the ^{29}Si chemical shifts (Table S14) were relatively less affected by the change of the solvent compared to the proton shifts. However, here the downfield trend is common for all negative chemical shifts (with respect to TMS), and upfield for positive chemical shifts (in the case of triethylsilane).

Table S12. The proton chemical shifts (in ppm) of silanes in various conformations, calculated at the PBE0-D3/def2-TZVPP level of theory using the COSMO continuum solvation model in benzene and o-DCB.

	Conformations	BEN	o-DCB
$(C_6F_5)_3$Si-H	1	6.612	6.612
	2	6.608	6.612
	3	6.608	6.611
	4	6.610	6.610
	5	6.608	6.610
$(Me_3Si)_3$Si-H	1	2.636	2.510
	2	2.638	2.515
	3	2.632	2.512
	4	2.638	2.516
	5	2.636	2.512
Et_3SiH	1	4.015	3.927
	2	4.018	3.928
	3	4.014	3.927
	4	4.018	3.928

	5	4.017	3.927
Ph_3SiH	1	6.199	6.168
	2	6.201	6.160
	3	6.199	6.138
	4	6.199	6.123
	5	6.198	6.166

Table S13. The proton chemical shifts (in ppm) of silanes in various conformations, calculated at the PBE0-D3/def2-SVP level of theory using the COSMO continuum solvation model in benzene and o-DCB.

	Conformations	BEN	Average	o-DCB	Average
$(C_6F_5)_3Si$-H	1	3.828	4.821	5.610	5.307
	2	3.991		5.116	
	3	4.799		5.115	
	4	5.601		5.116	
	5	5.606		5.441	
	6	5.101		5.442	
$(Me_3Si)_3Si$-H	1	2.218	1.790	0.978	0.960
	2	1.504		0.378	
	3	1.503		0.185	
	4	1.556		0.765	
	5	2.329		1.582	
	6	1.631		1.871	
Et_3SiH	1	3.211	3.165	2.819	2.780
	2	3.594		2.798	
	3	3.267		2.715	
	4	2.710		2.782	
	5	3.005		2.795	
	6	3.203		2.769	

Table S14. ^{29}Si chemical shifts of the tested silanes in aromatic solvents.

solvent	^{29}Si δ (ppm)			
	**TPFPS**	**TTMSS**	**TES**	**TPS**
benzene-D_6	-54.7	-115.56	0.52	-17.49
oDCB-D_4	-54.3	-115.34	0.45	-17.45
CD_2Cl_2	-54.7	-114.85	0.93	-17.68

NMR data

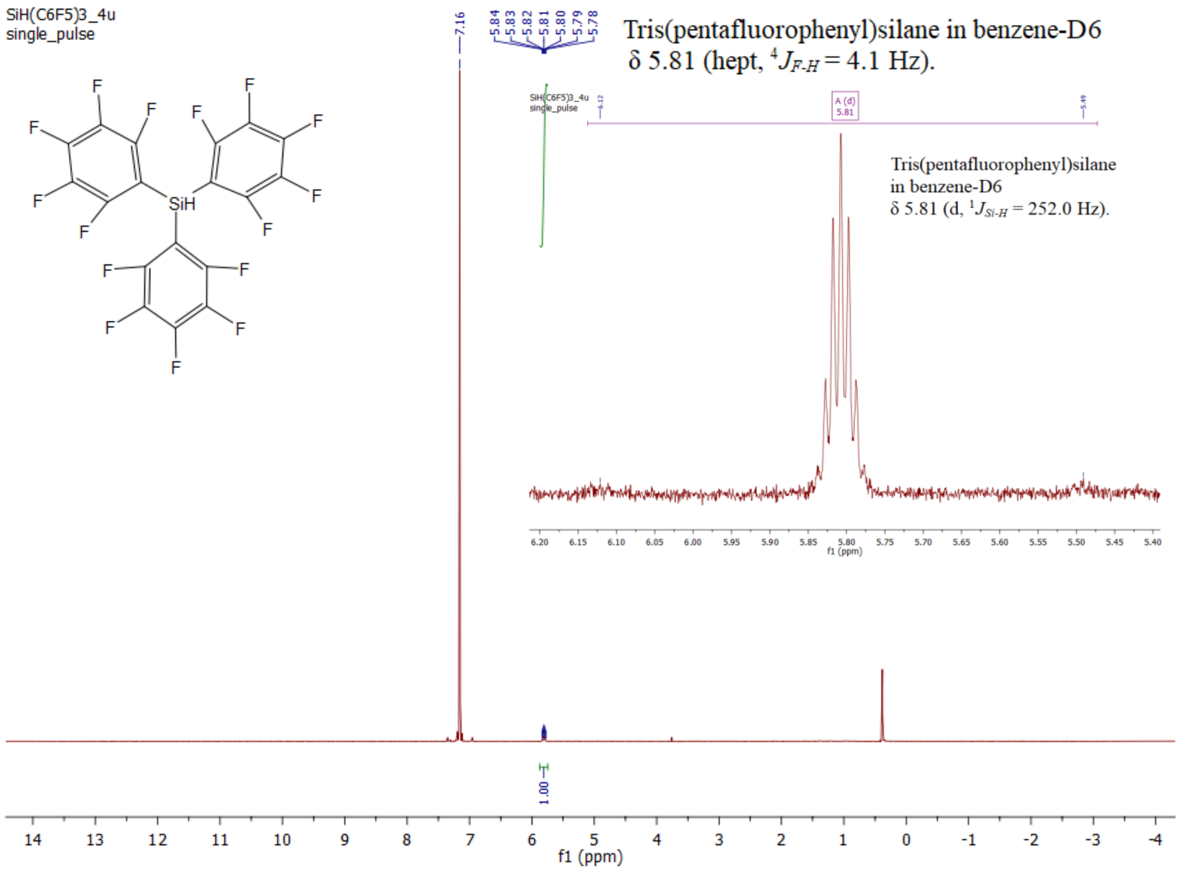

Figure S15. ^{1}H NMR spectrum of tris(pentafluorophenyl)silane in benzene-D_6 with the magnified region of the Si-H proton signal in the inset.

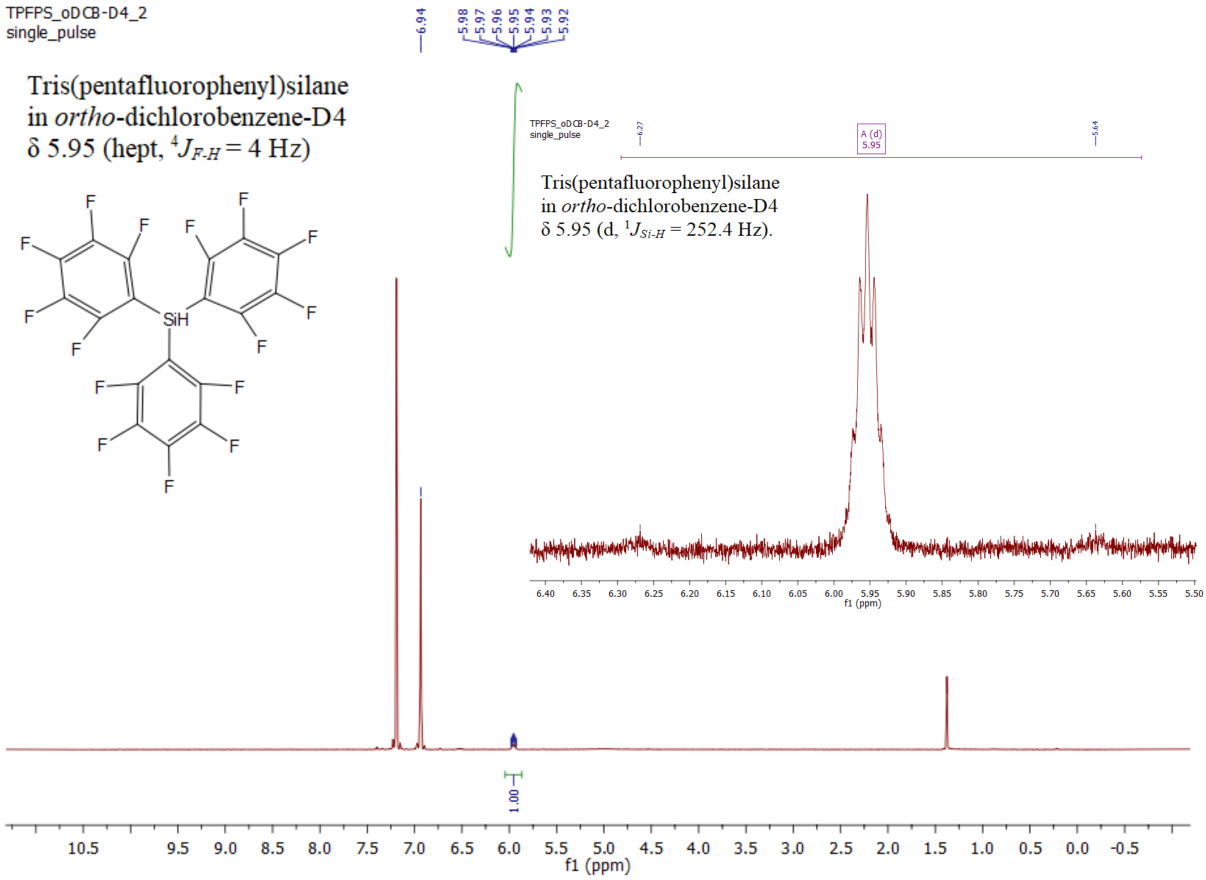

Figure S16. ^{1}H NMR spectrum of tris(pentafluorophenyl)silane in oDCB-D_4 with the magnified region of the Si-H proton signal in the inset.

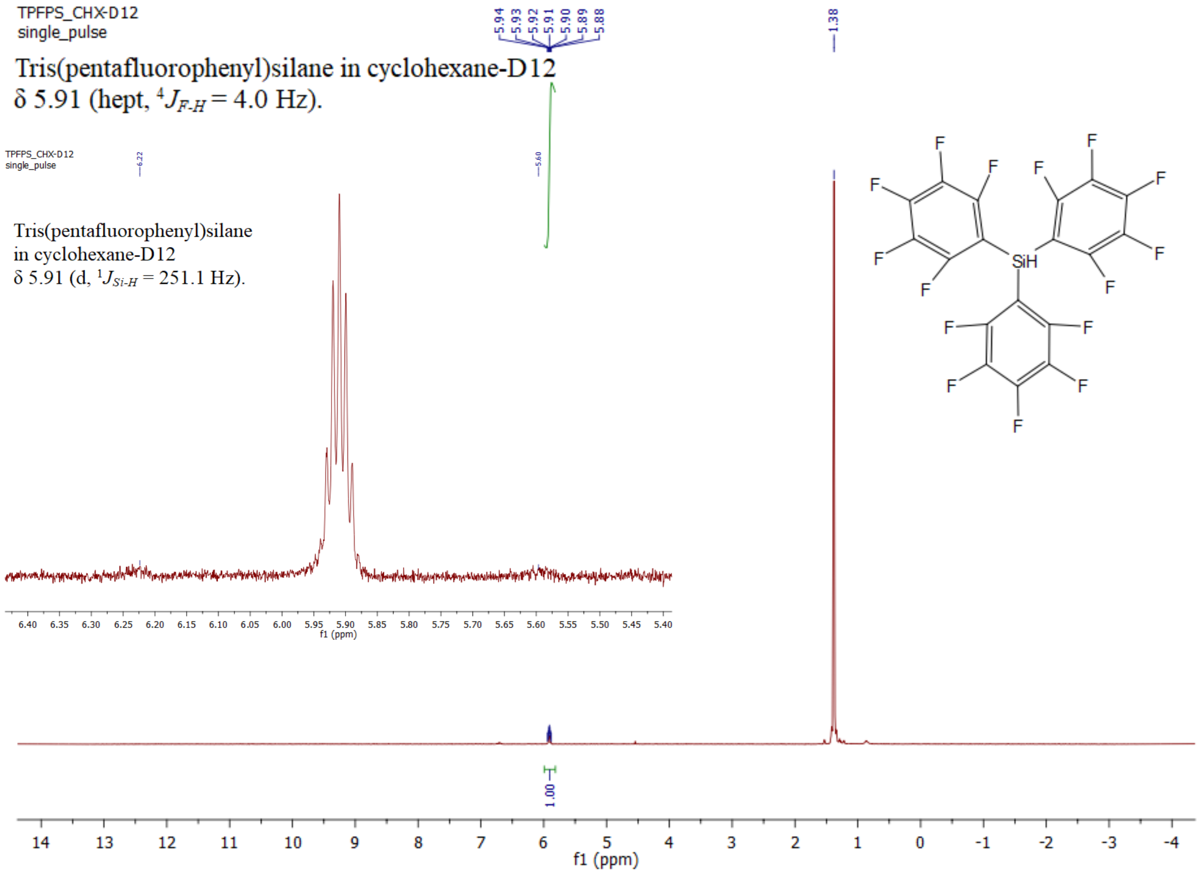

Figure S17. ^{1}H NMR spectrum of tris(pentafluorophenyl)silane in cyclohexane-D_{12} with the magnified region of the Si-H proton signal in the inset.

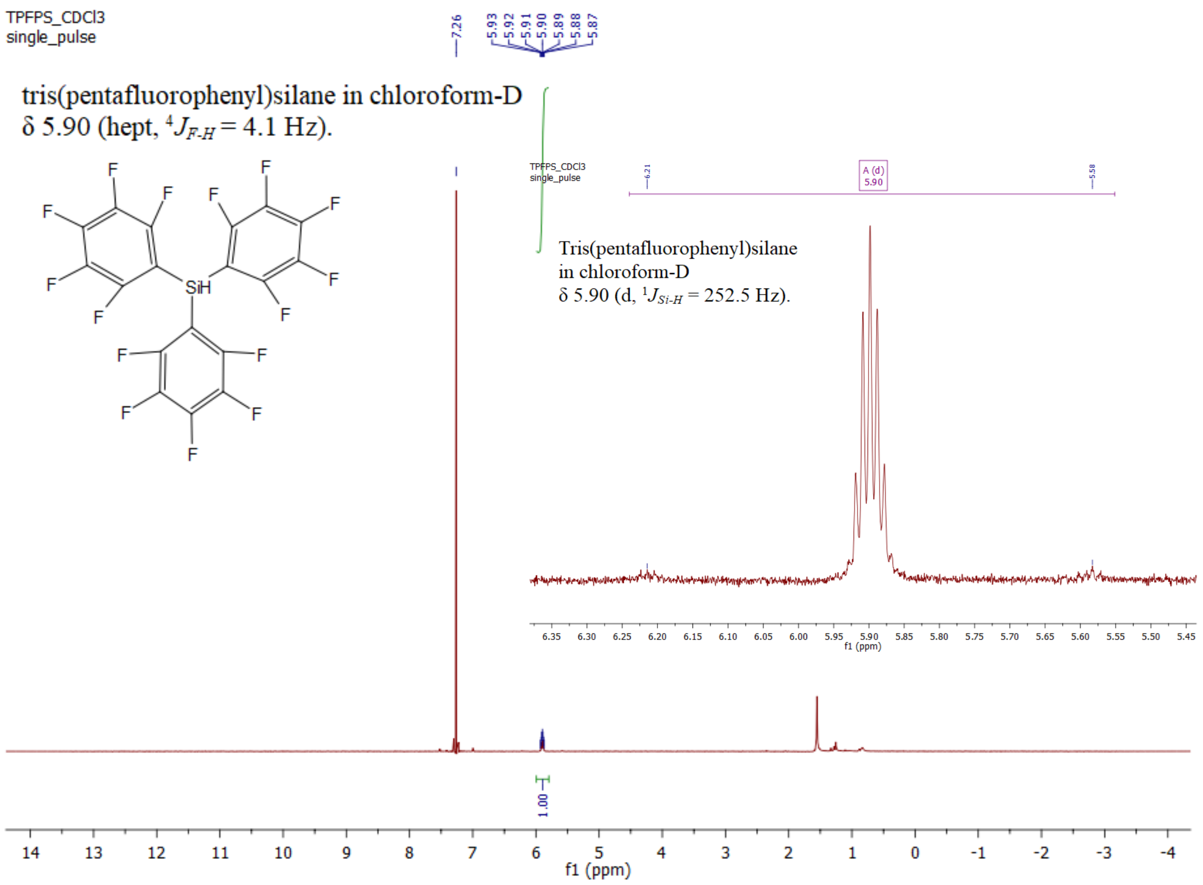

Figure S18. ^{1}H NMR spectrum of tris(pentafluorophenyl)silane in $CDCl_3$ with the magnified region of the Si-H proton signal in the inset.

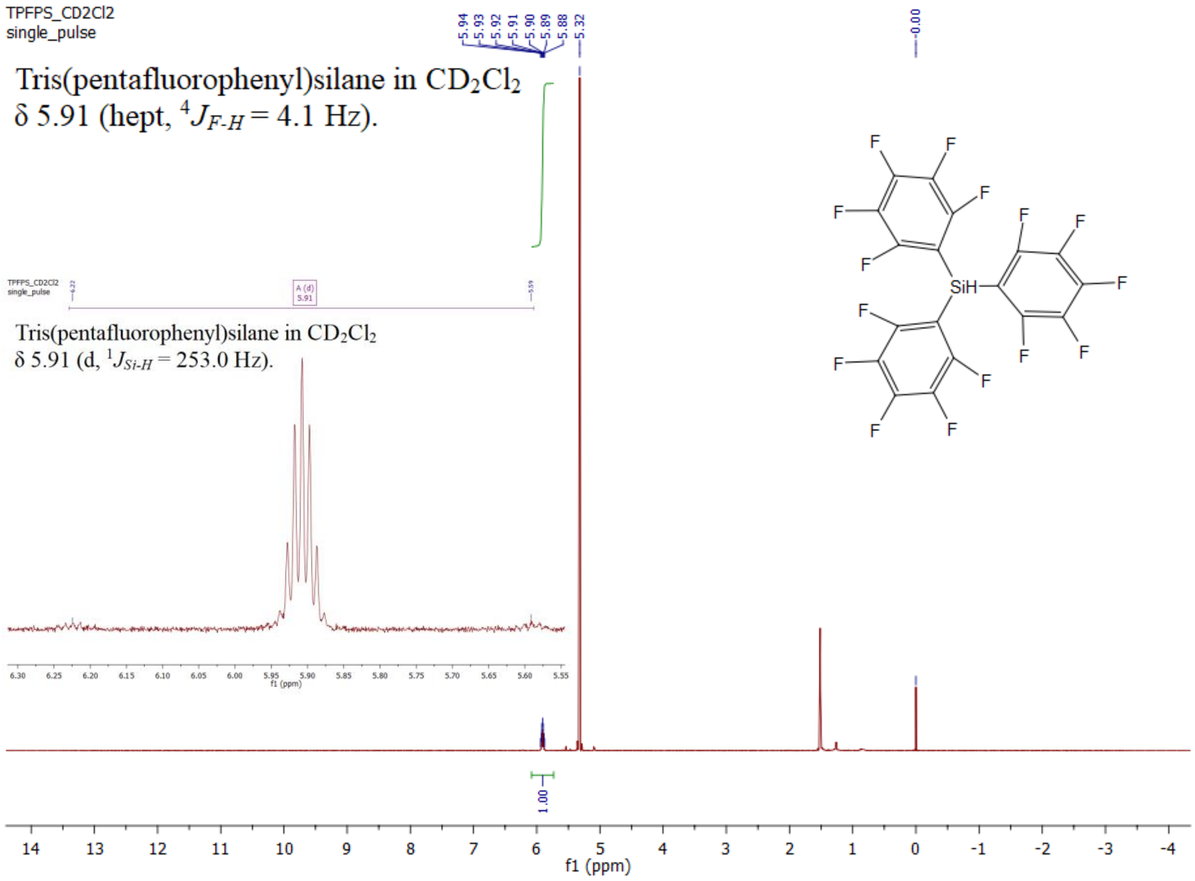

Figure S19. ^1H NMR spectrum of tris(pentafluorophenyl)silane in CD_2Cl_2 with the magnified region of the Si-H proton signal in the inset.

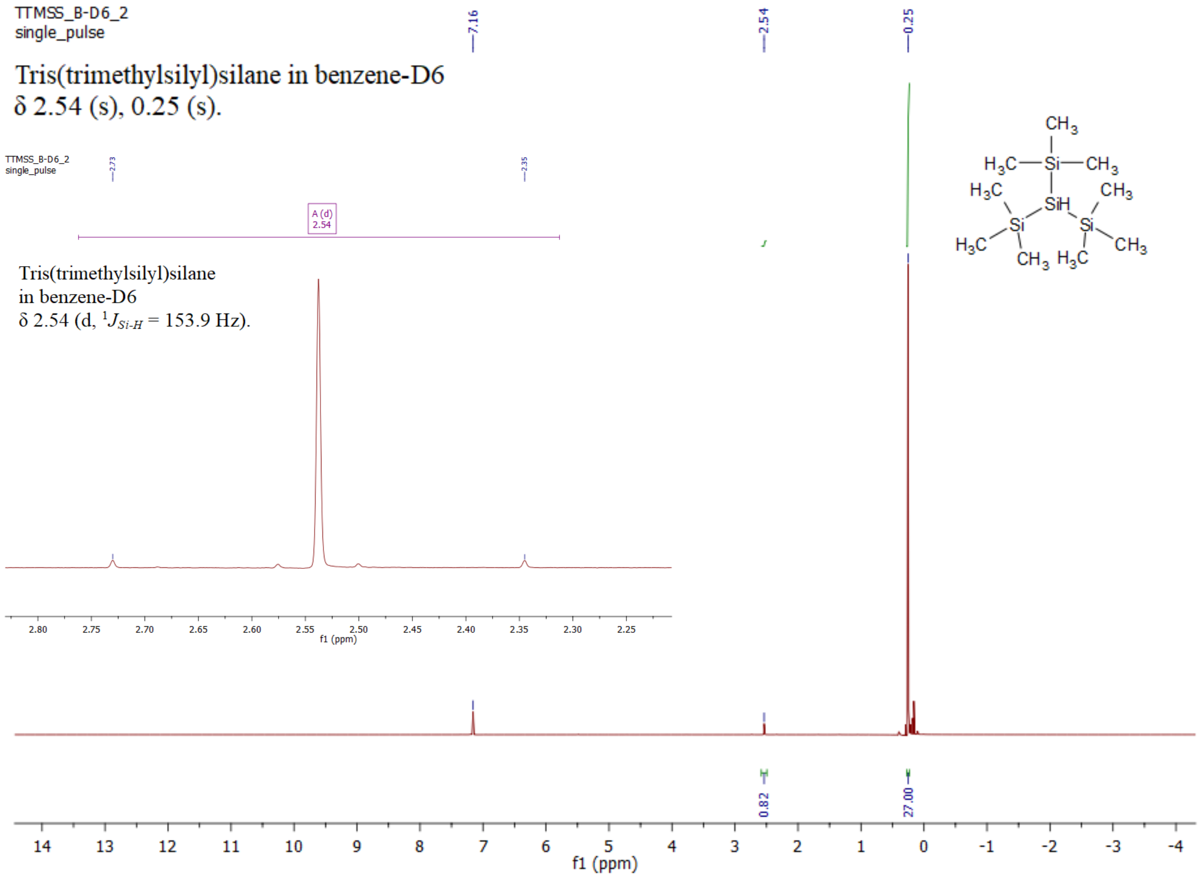

Figure S20. ^{1}H NMR spectrum of tris(trimethylsilyl)silane in benzene-D_6 with the magnified region of the Si-H proton signal in the inset.

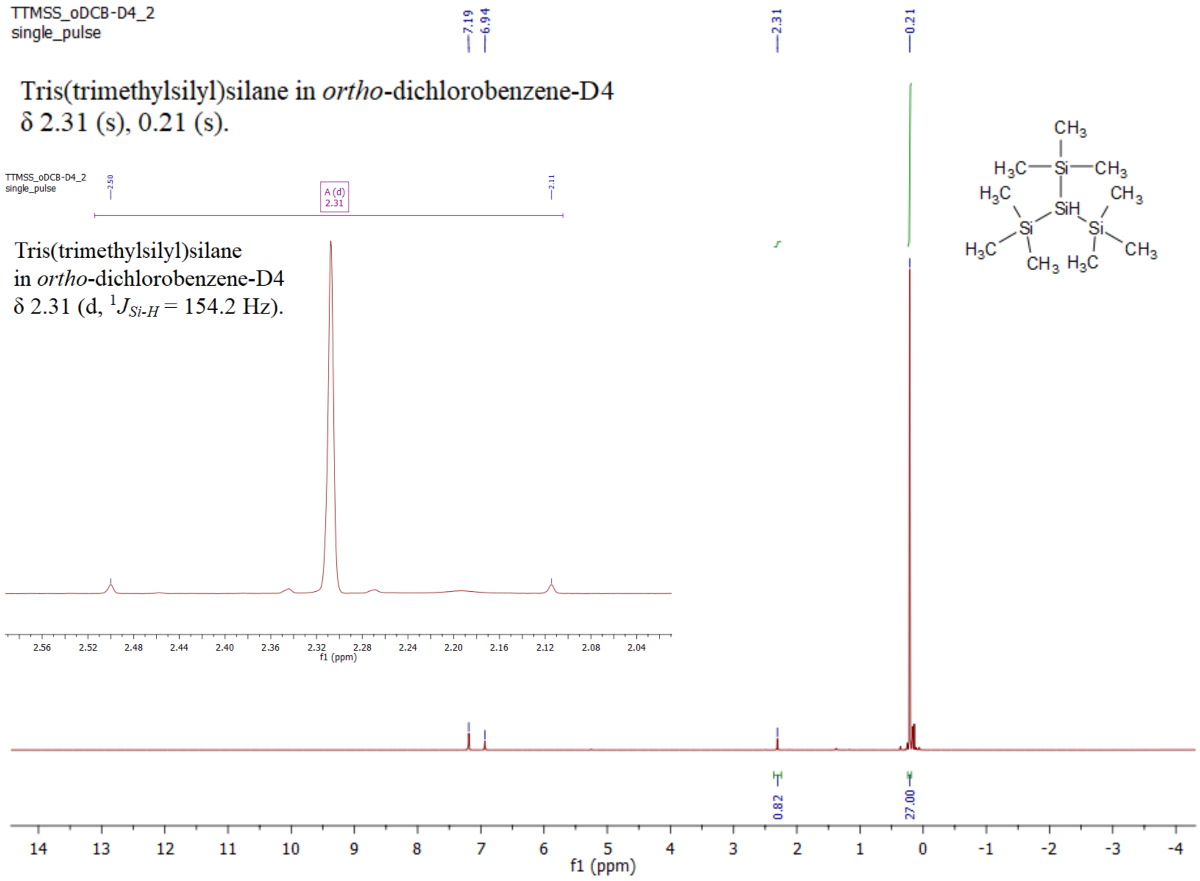

Figure S21. ^{1}H NMR spectrum of tris(trimethylsilyl)silane in oDCB-D_4 with the magnified region of the Si-H proton signal in the inset.

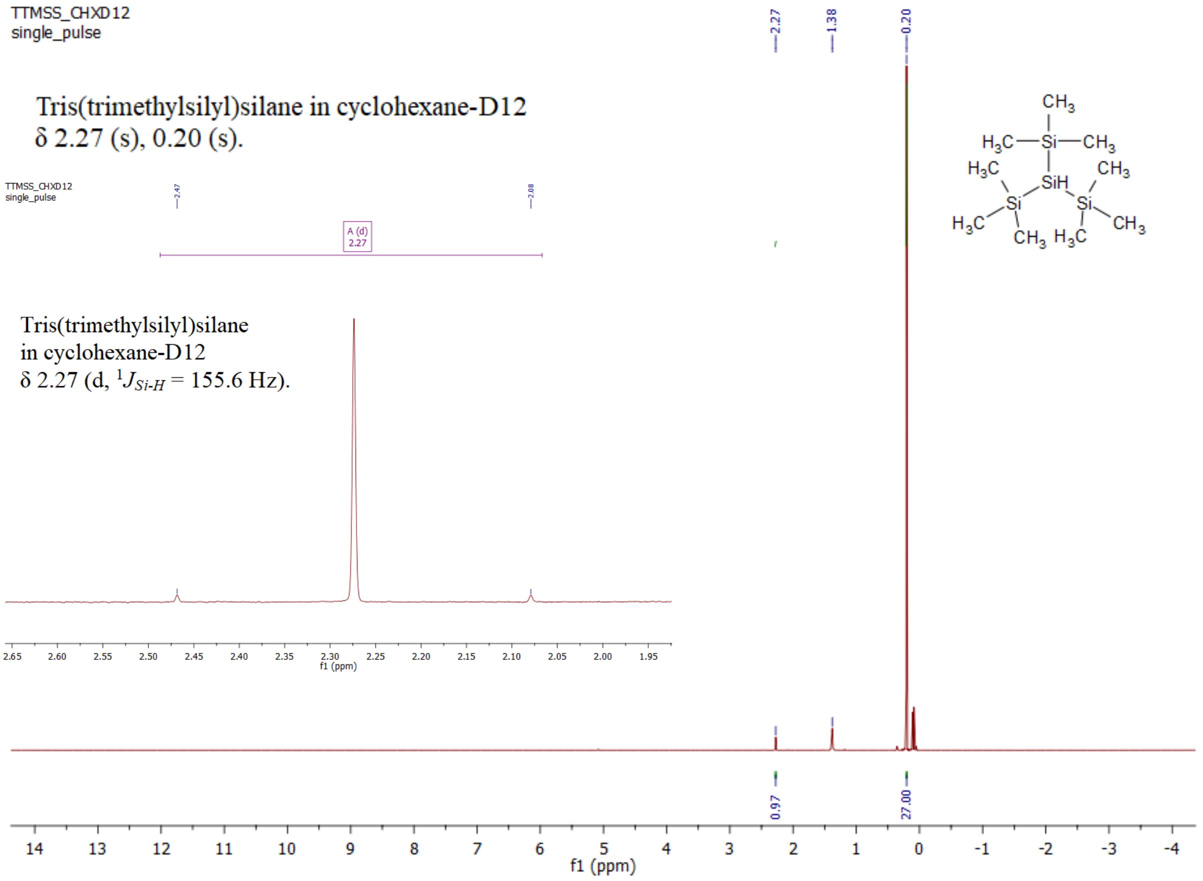

Figure S22. ^{1}H NMR spectrum of tris(trimethylsilyl)silane in cyclohexane-D_{12} with the magnified region of the Si-H proton signal in the inset.

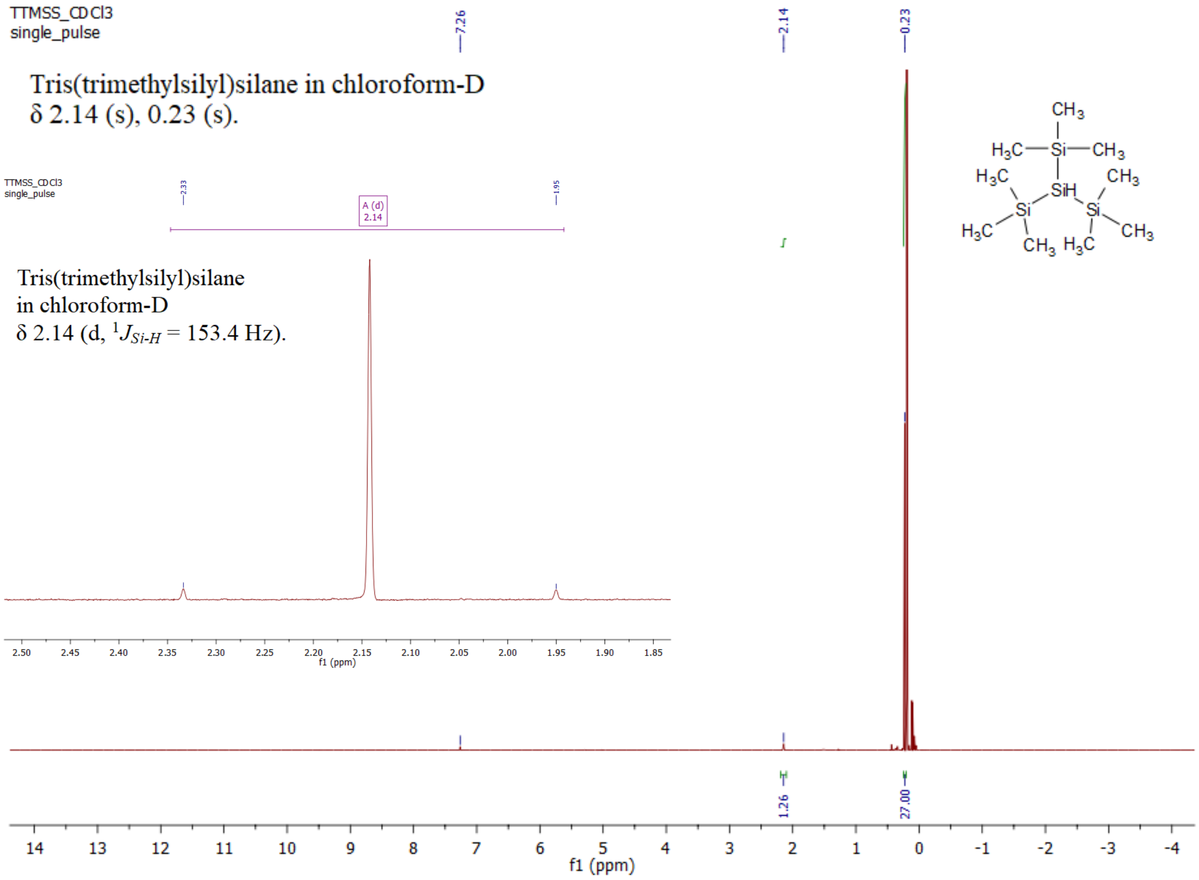

Figure S23. ^{1}H NMR spectrum of tris(trimethylsilyl)silane in $CDCl_3$ with the magnified region of the Si-H proton signal in the inset.

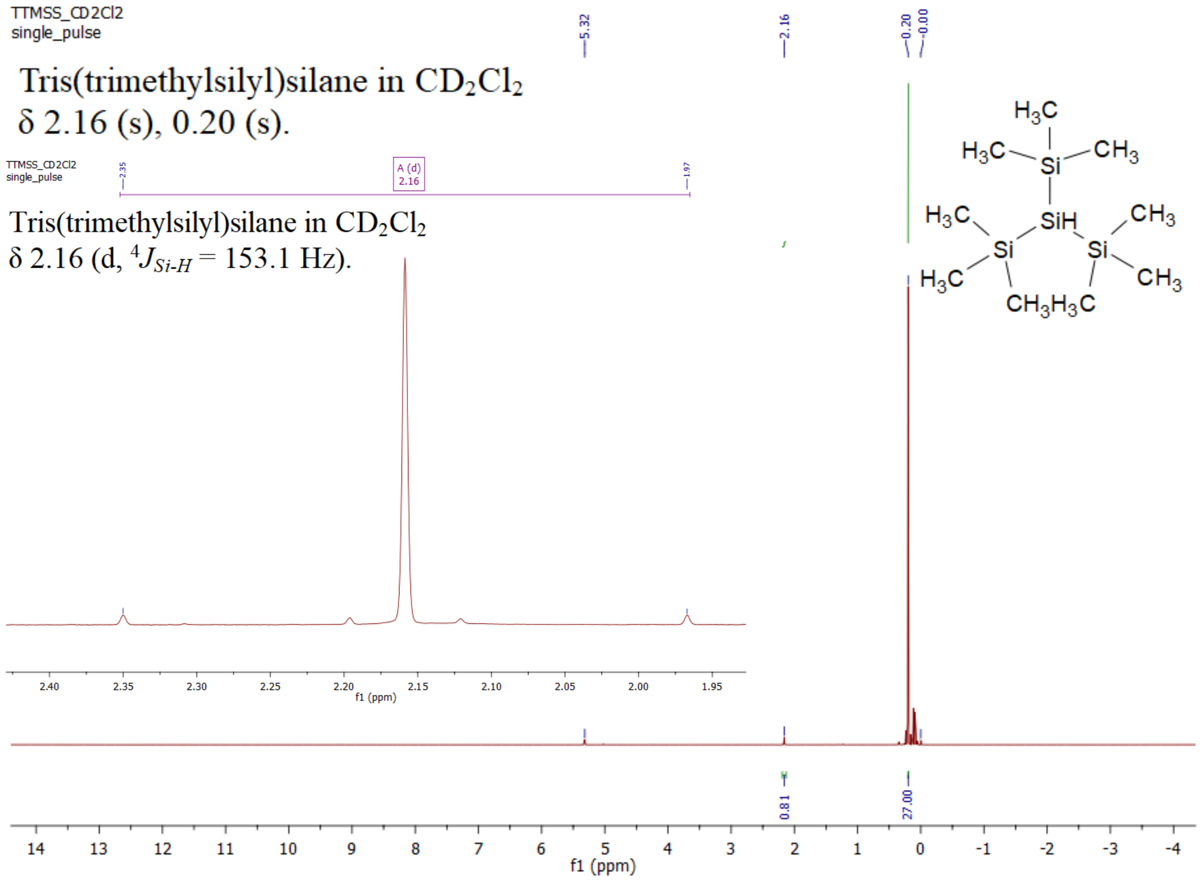

Figure S24. ^{1}H NMR spectrum of tris(trimethylsilyl)silane in CD_2Cl_2 with the magnified region of the Si-H proton signal in the inset.

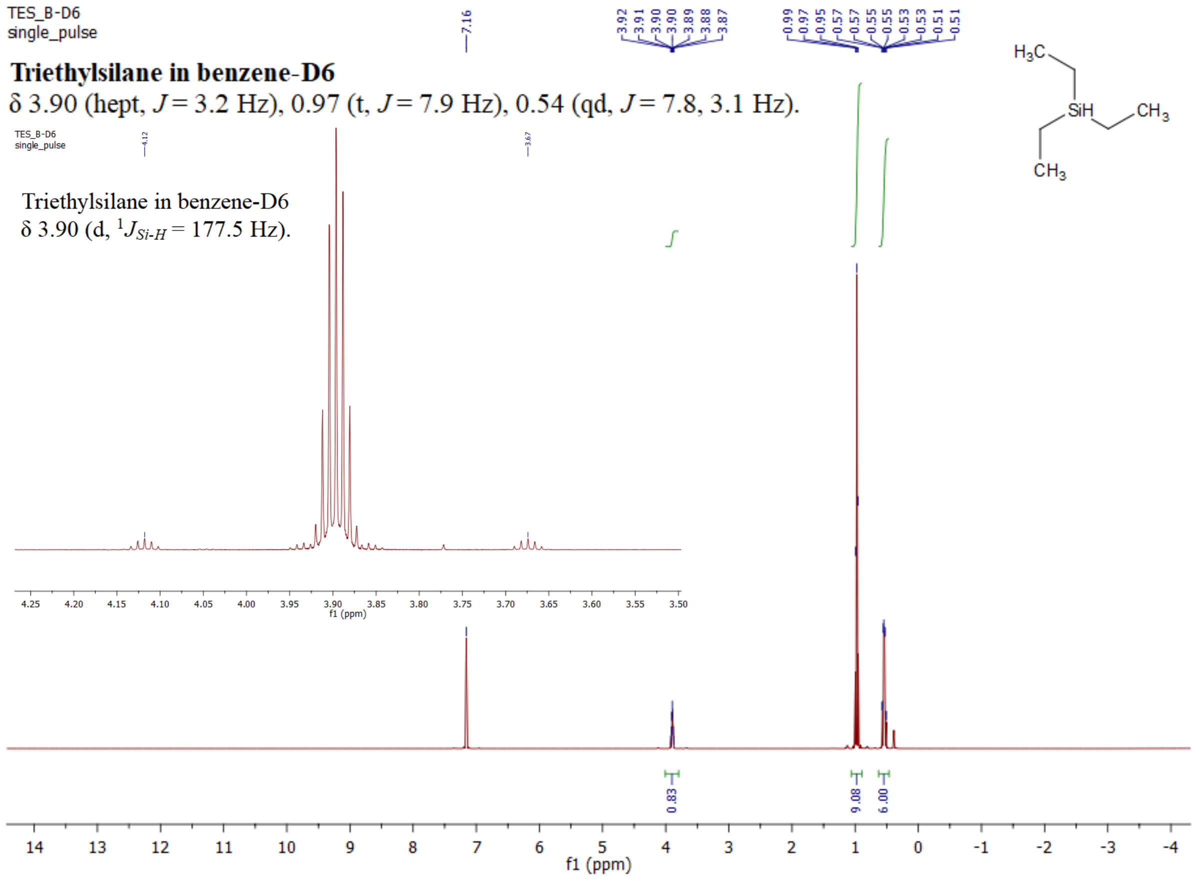

Figure S25. ^{1}H NMR spectrum of triethylsilane in benzene-D_6 with the magnified region of the Si-H proton signal in the inset.

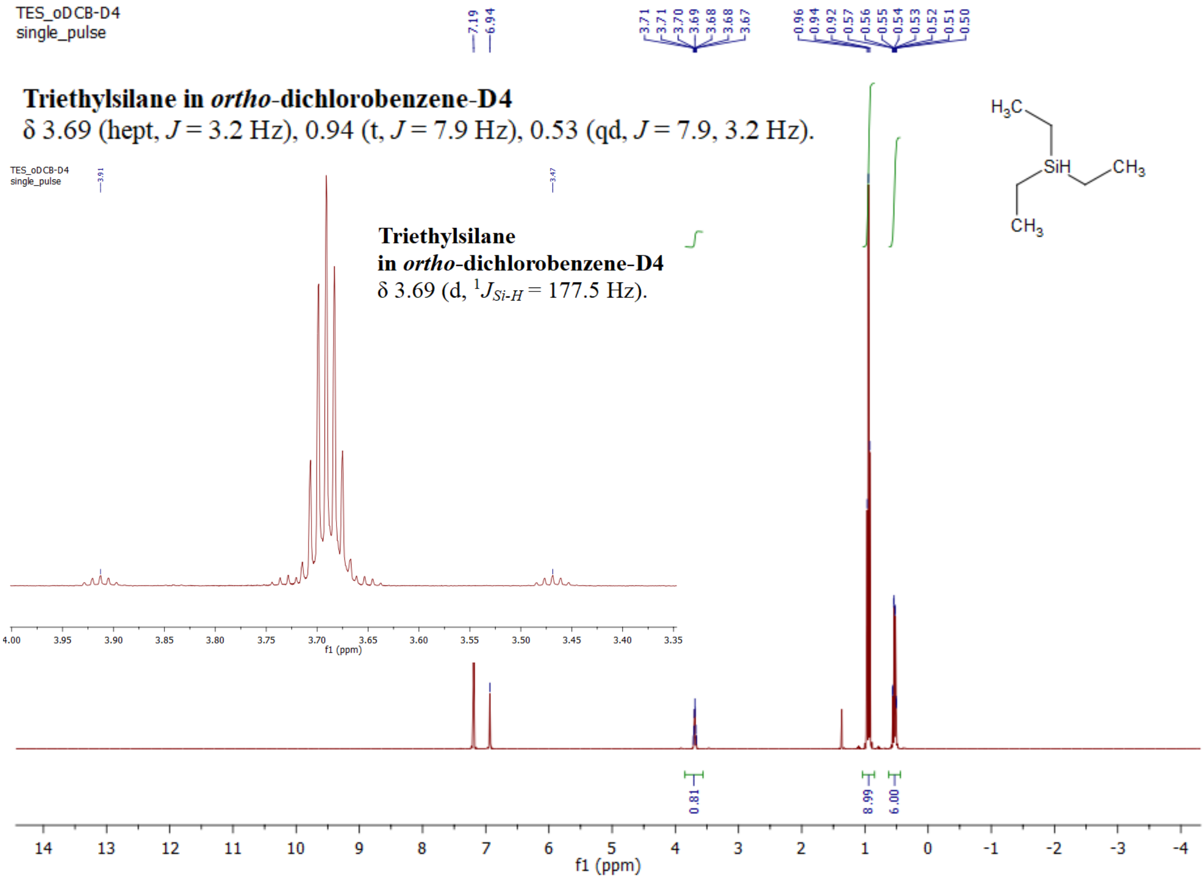

Figure S26. ^{1}H NMR spectrum of triethylsilane in oDCB-D_4 with the magnified region of the Si-H proton signal in the inset.

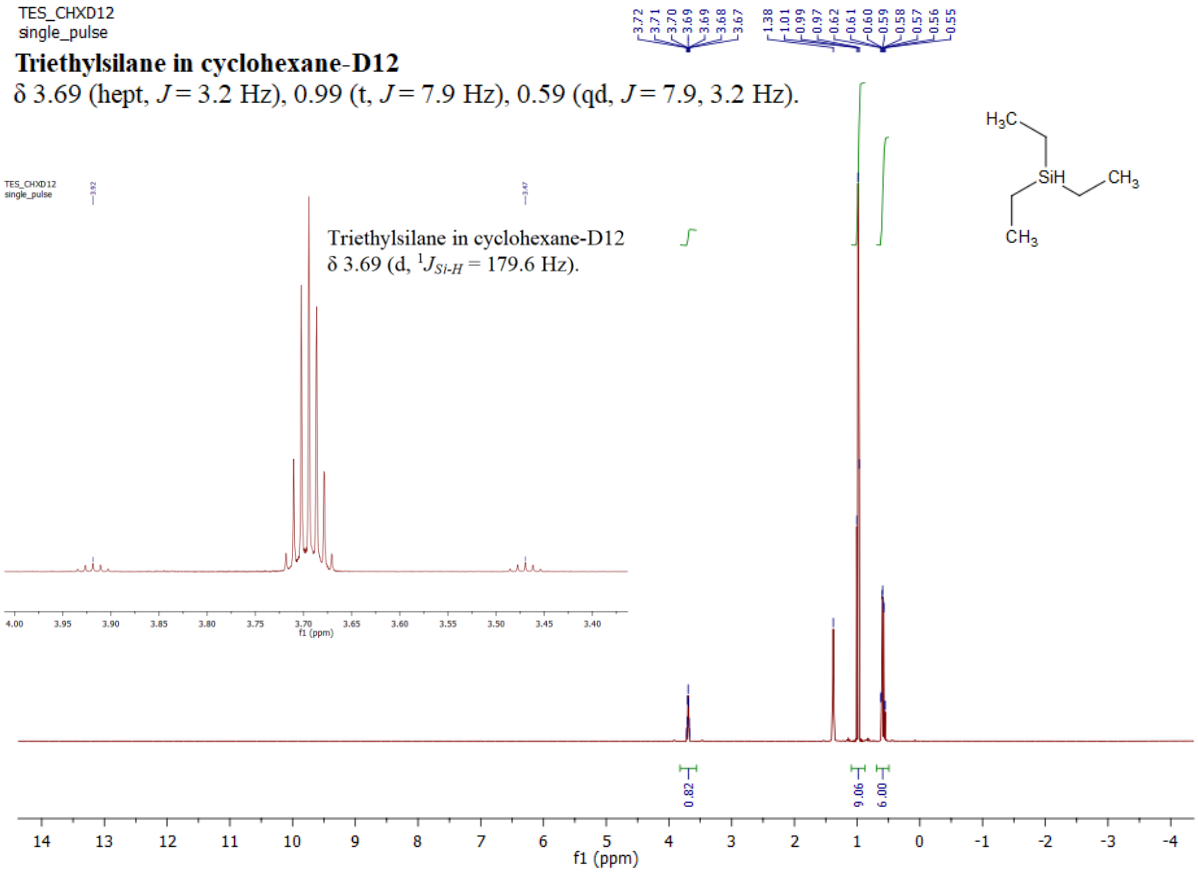

Figure S27. ^{1}H NMR spectrum of triethylsilane in cyclohexane-D_{12} with the magnified region of the Si-H proton signal in the inset.

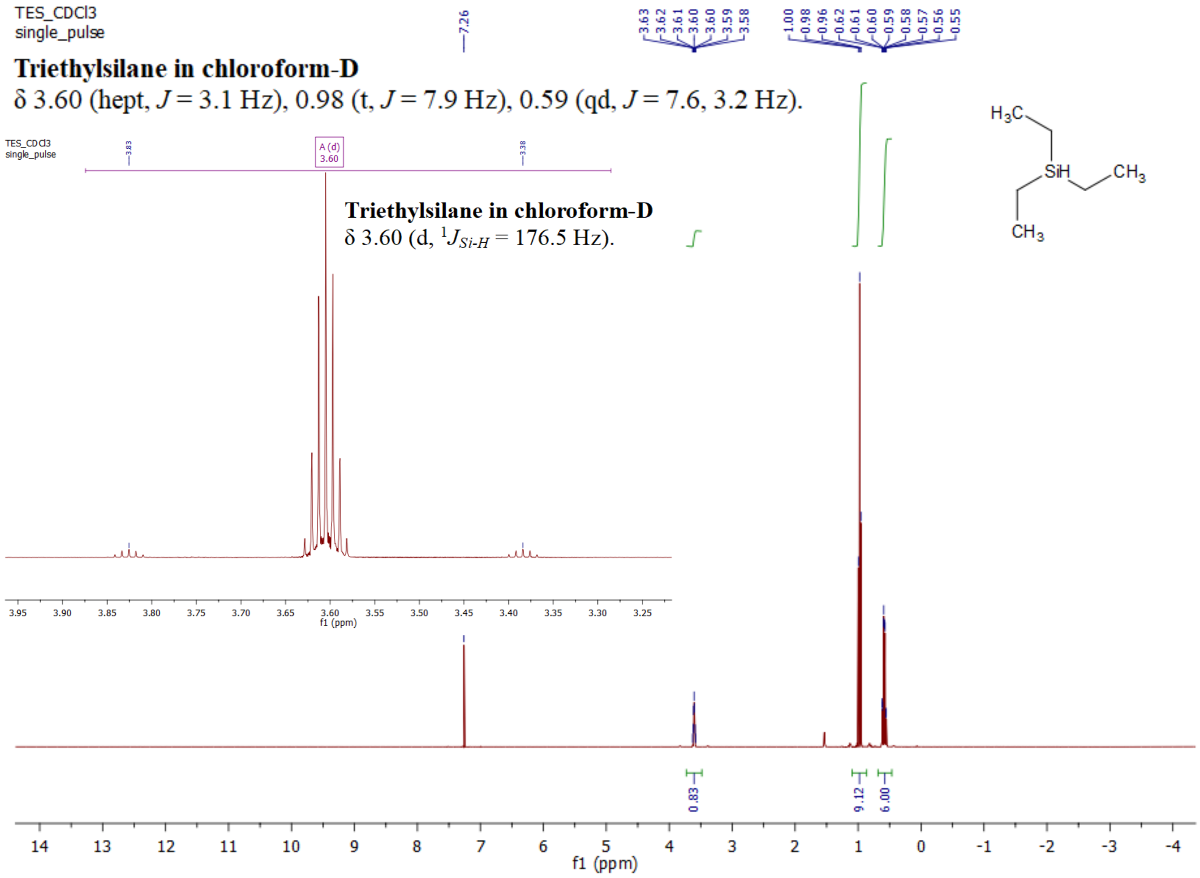

Figure S28. ^{1}H NMR spectrum of triethylsilane in $CDCl_3$ with the magnified region of the Si-H proton signal in the inset.

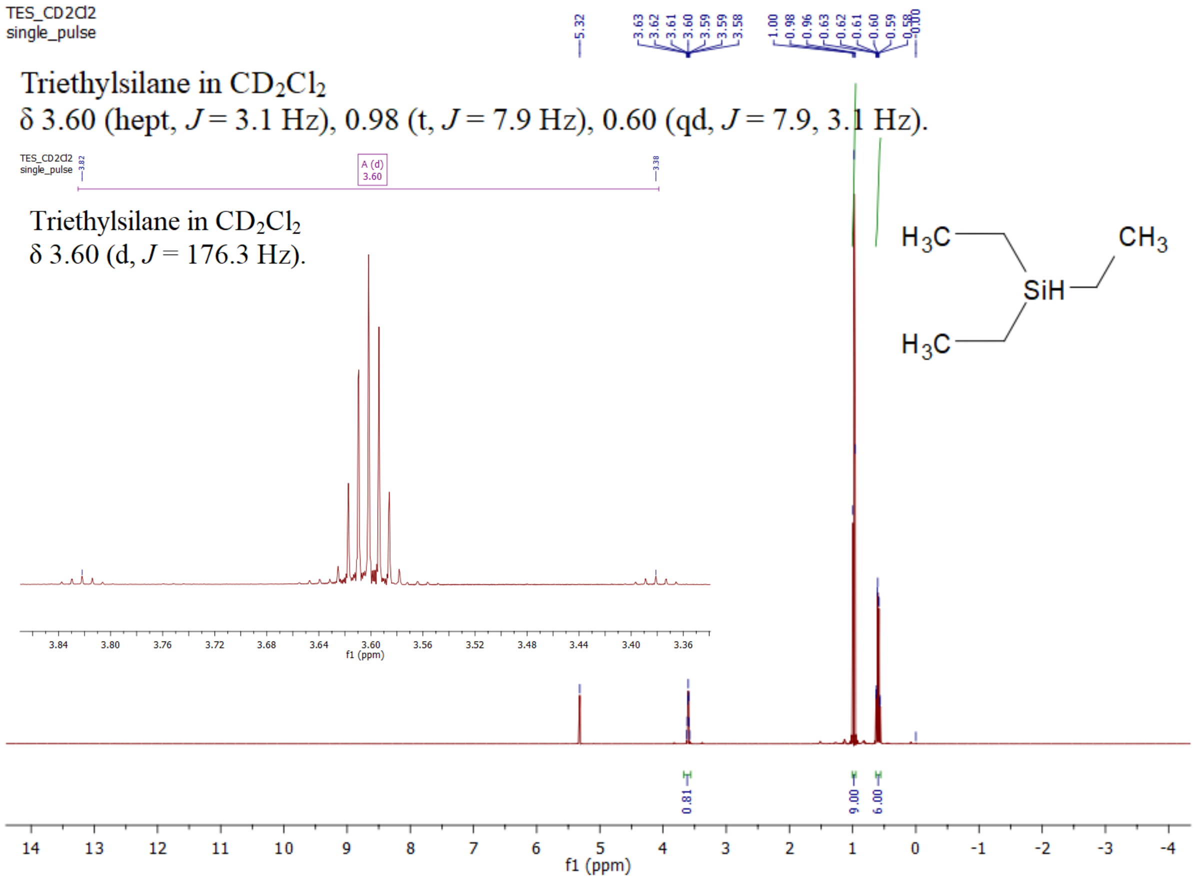

Figure S29. ^{1}H NMR spectrum of triethylsilane in CD_2Cl_2 with the magnified region of the Si-H proton signal in the inset.

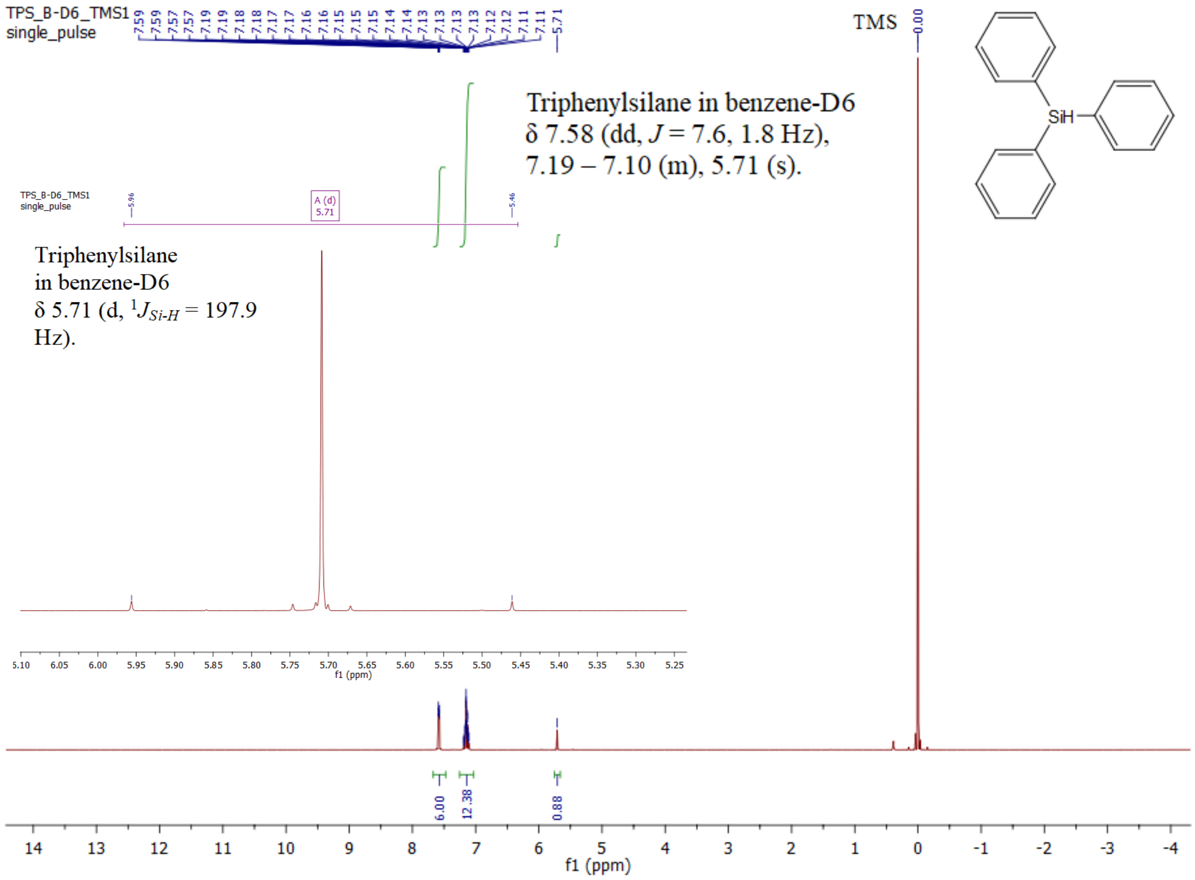

Figure S30. ^{1}H NMR spectrum of triphenylsilane in benzene-D$_6$ with the magnified region of the Si-H proton signal in the inset.

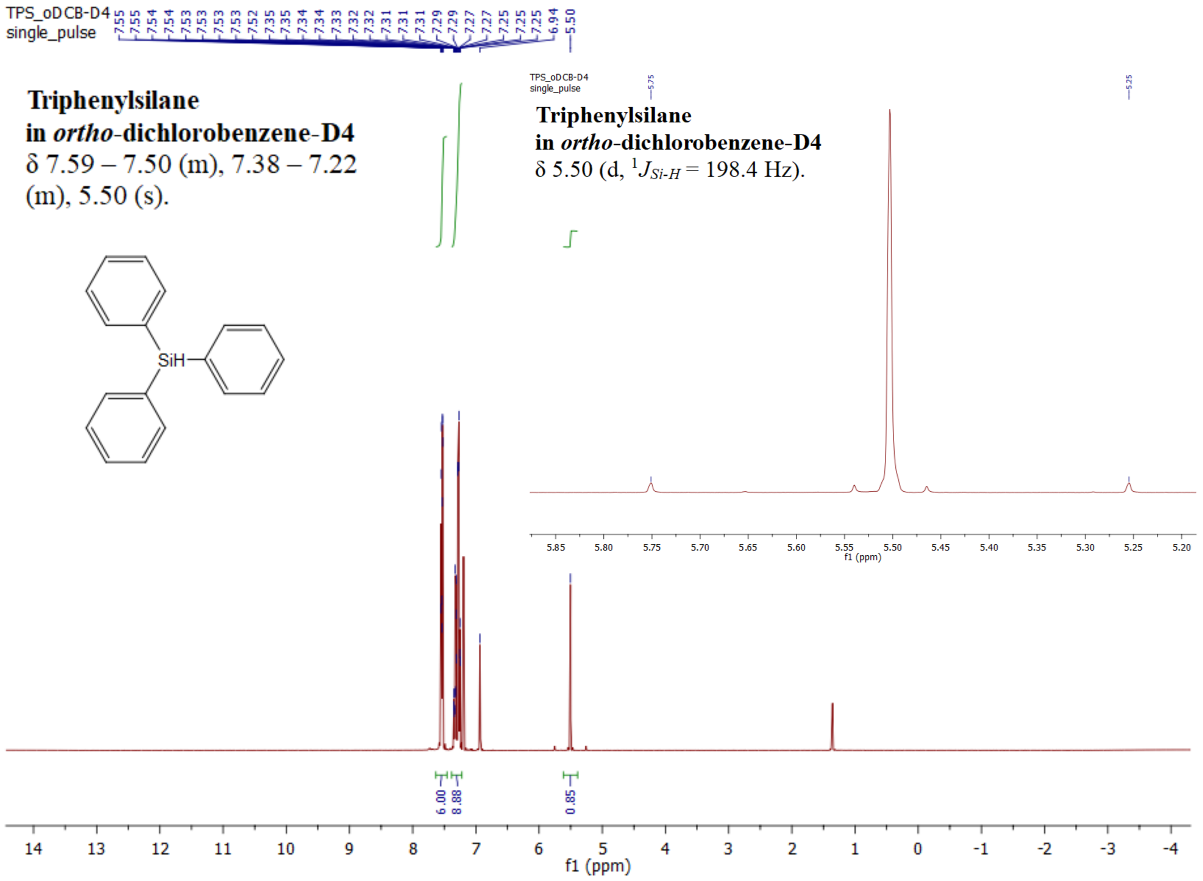

Figure S31. ^{1}H NMR spectrum of triphenylsilane in oDCB-D$_4$ with the magnified region of the Si-H proton signal in the inset.

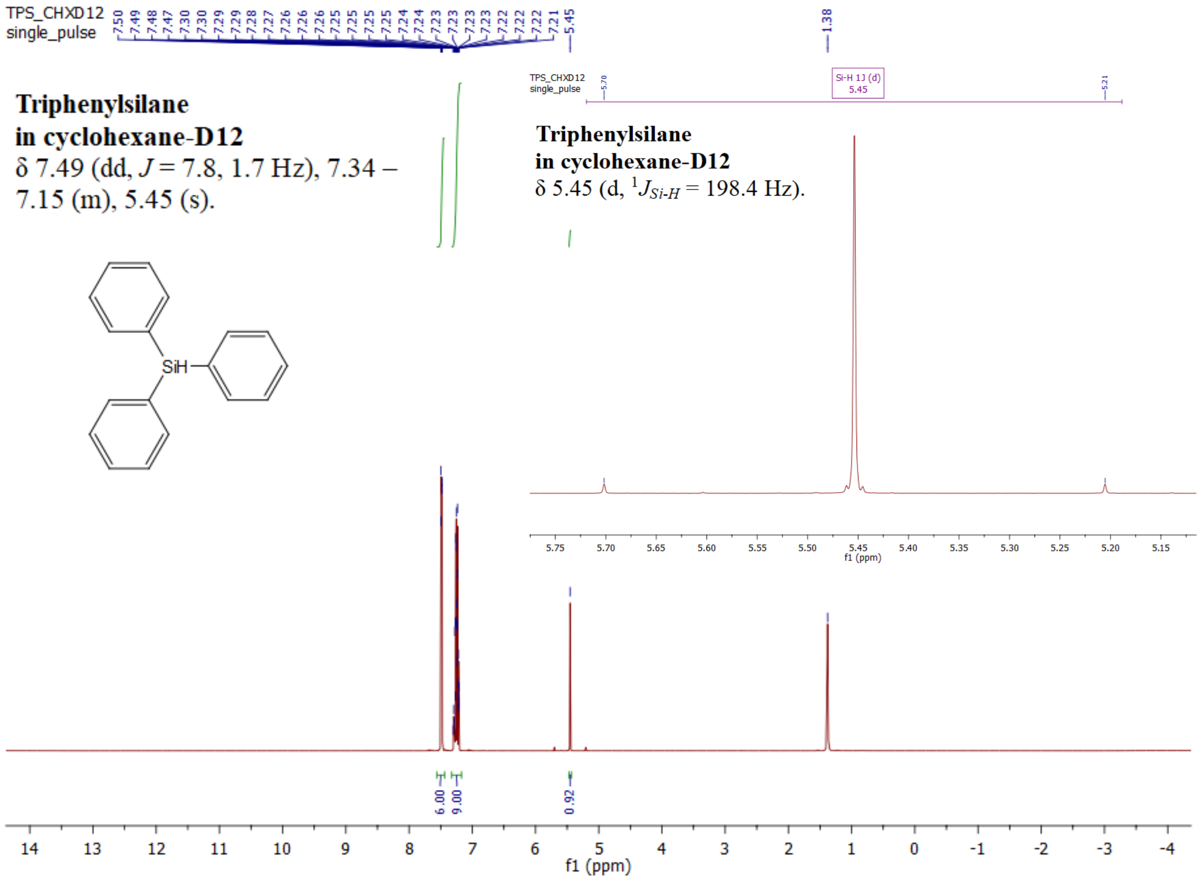

Figure S32. ^{1}H NMR spectrum of triphenylsilane in cyclohexane-D_{12} with the magnified region of the Si-H proton signal in the inset.

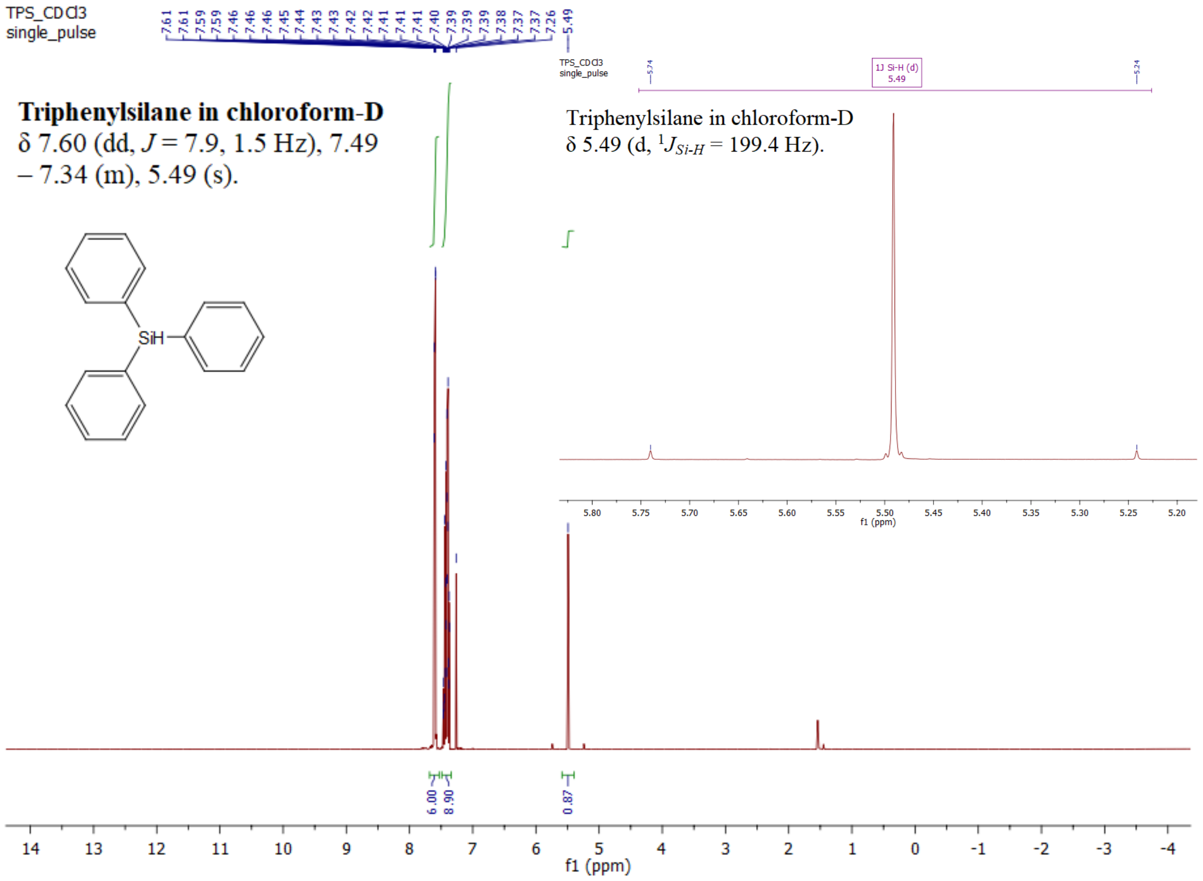

Figure S33. ^{1}H NMR spectrum of triphenylsilane in $CDCl_3$ with the magnified region of the Si-H proton signal in the inset.

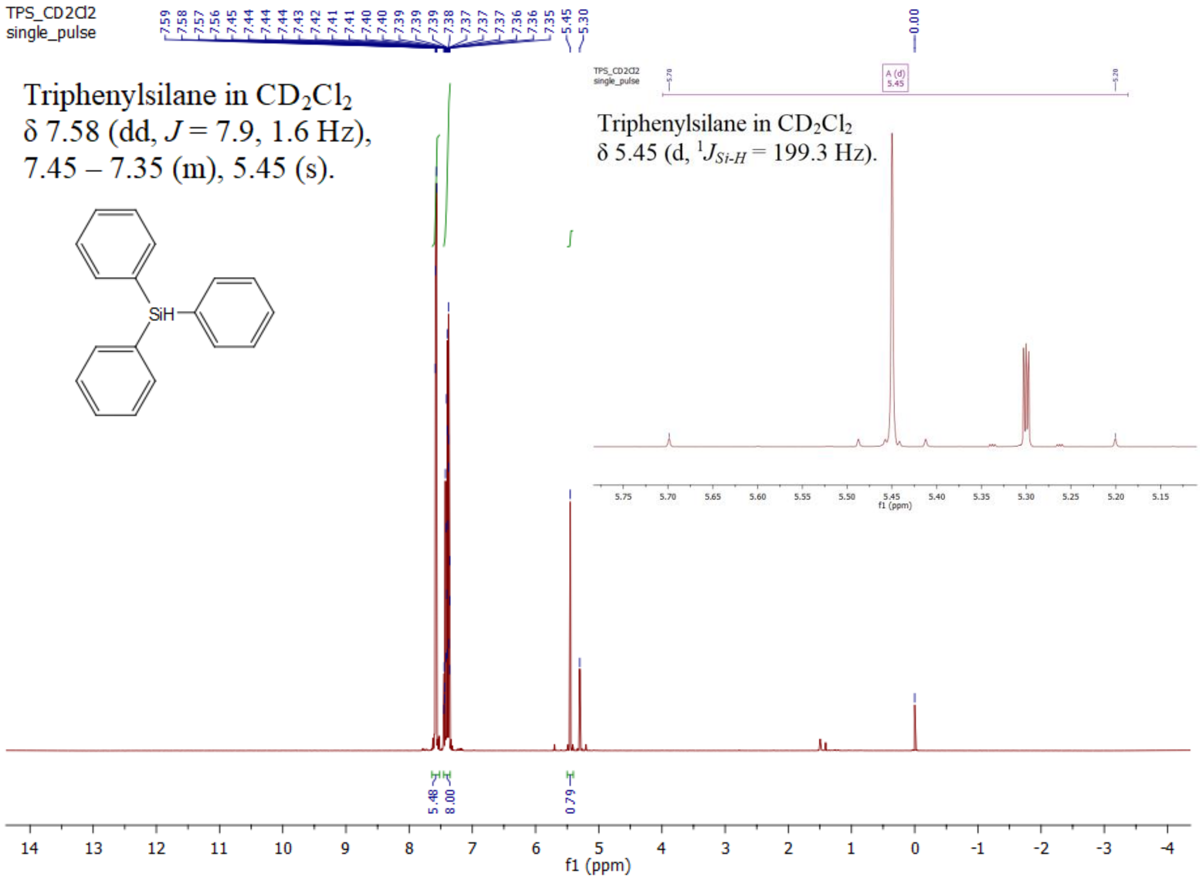

Figure S34. ^{1}H NMR spectrum of triphenylsilane in CD_2Cl_2 with the magnified region of the Si-H proton signal in the inset.

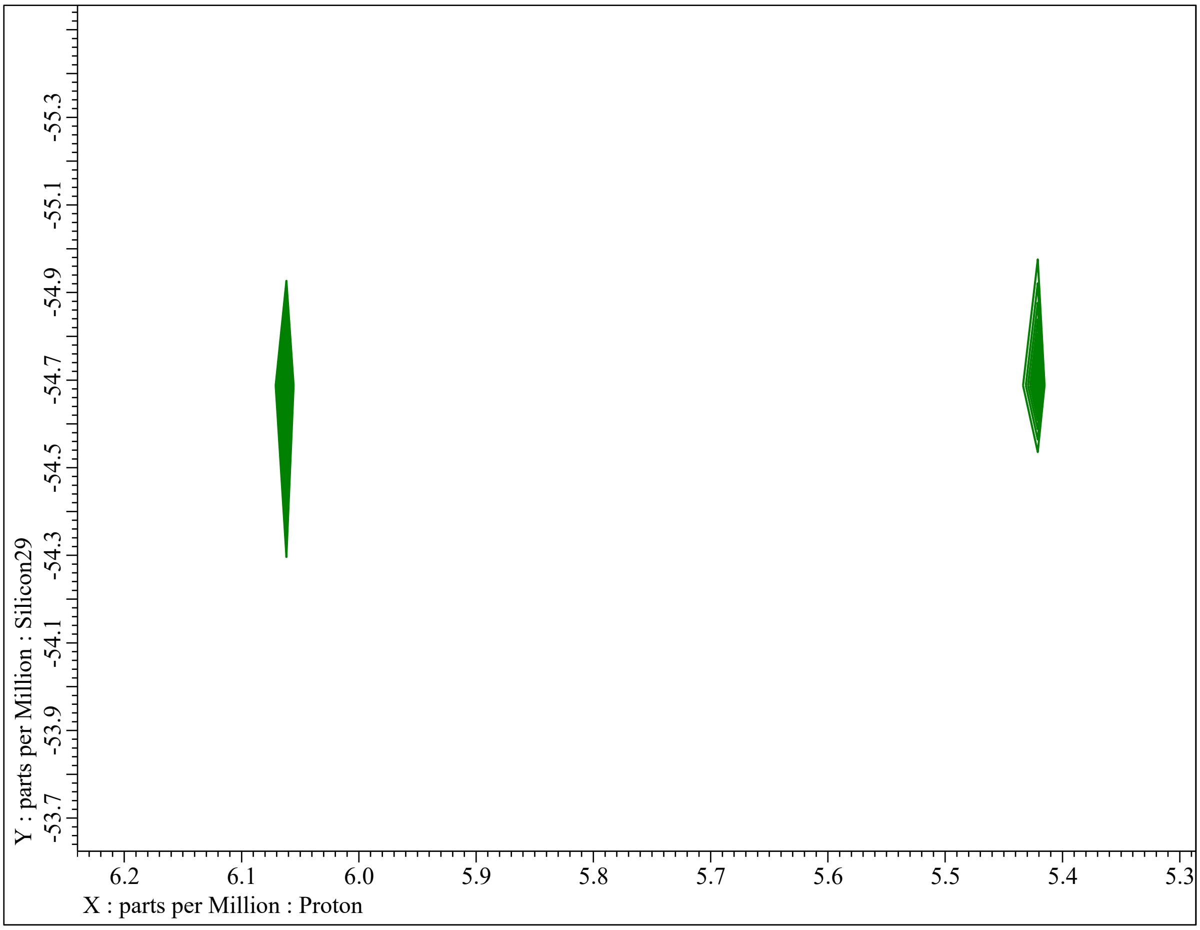

Figure S35. ^{1}H-^{29}Si HMQC spectrum of tris(pentafluorophenyl)silane in benzene-D_6 solvent.

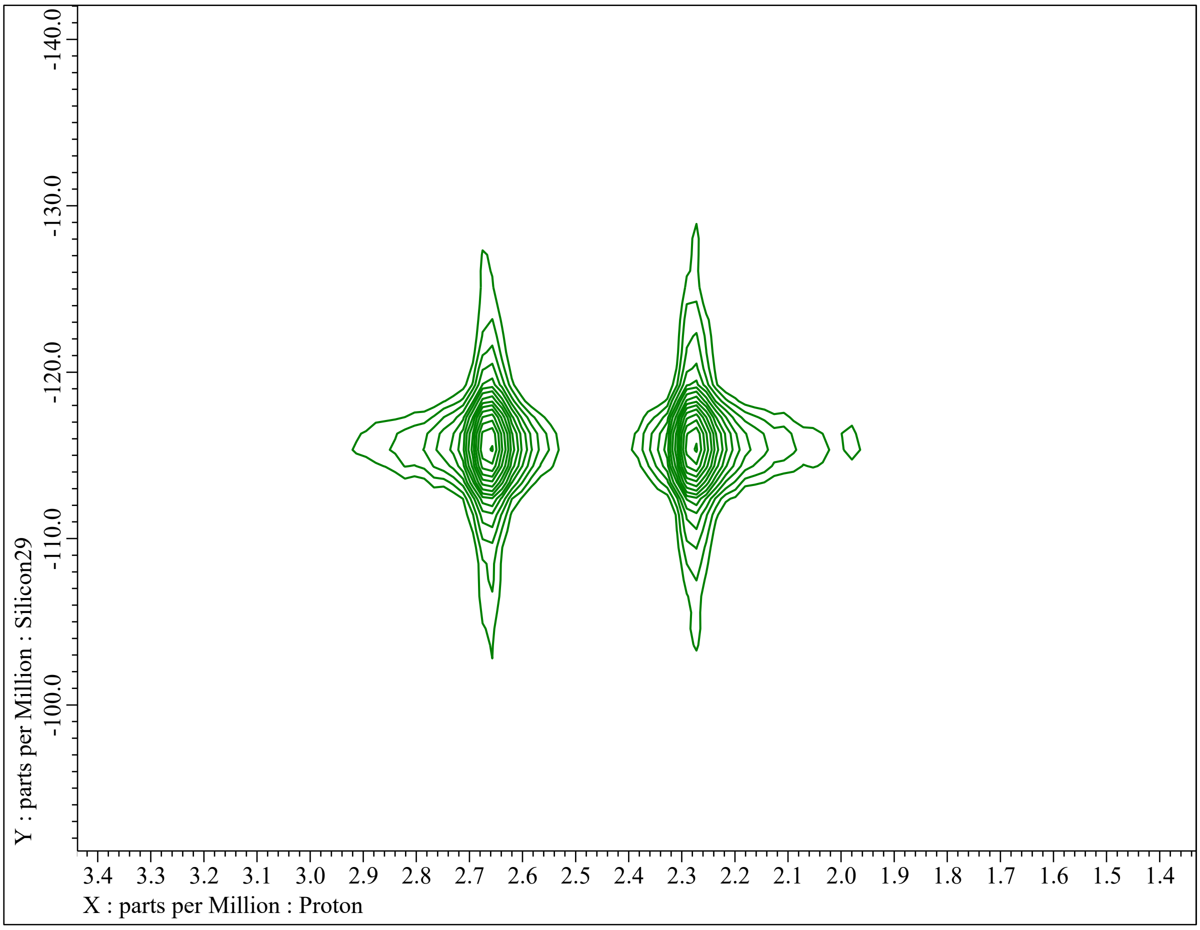

Figure S36. ^{1}H-^{29}Si HMQC spectrum of tris(trimethylsilyl)silane in benzene-D_6 solvent.

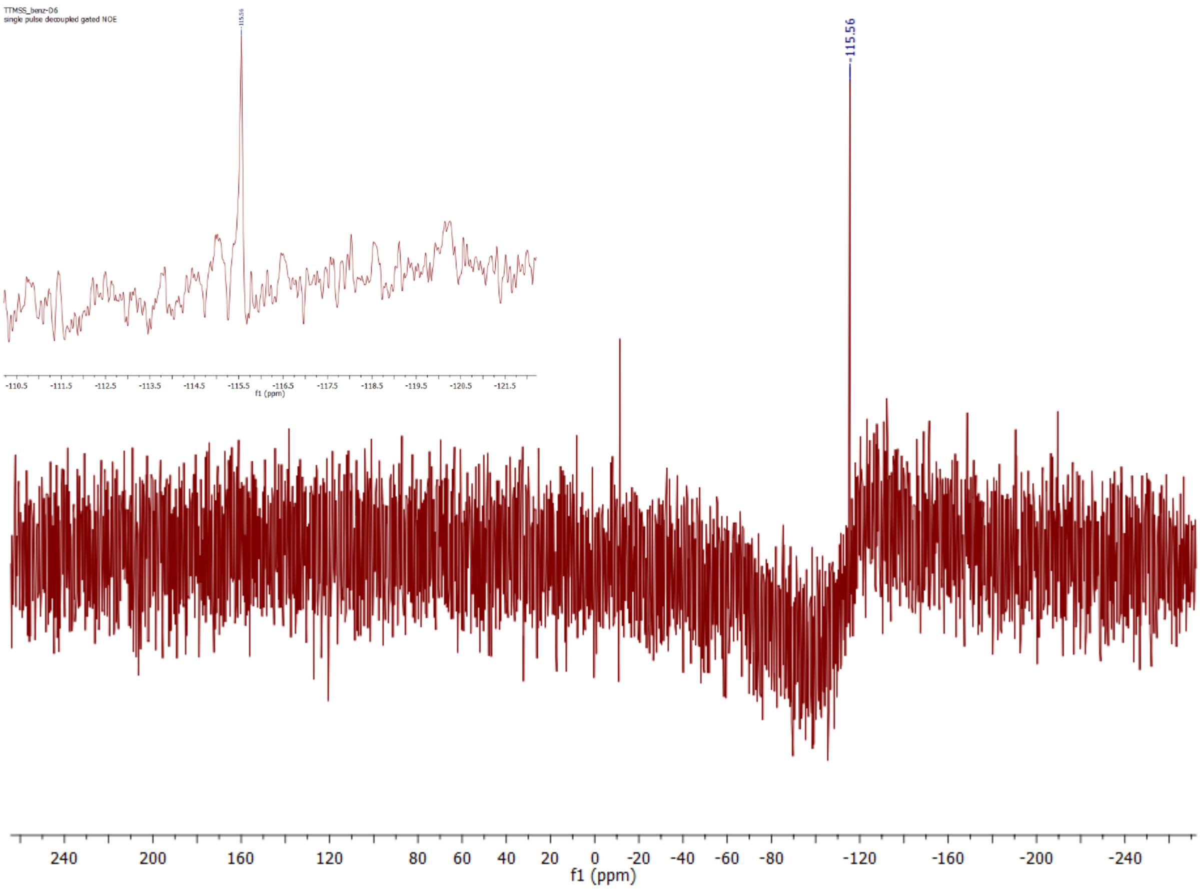

Figure S37. ^{29}Si spectrum of tris(trimethylsilyl)silane in benzene-D_6 solvent.

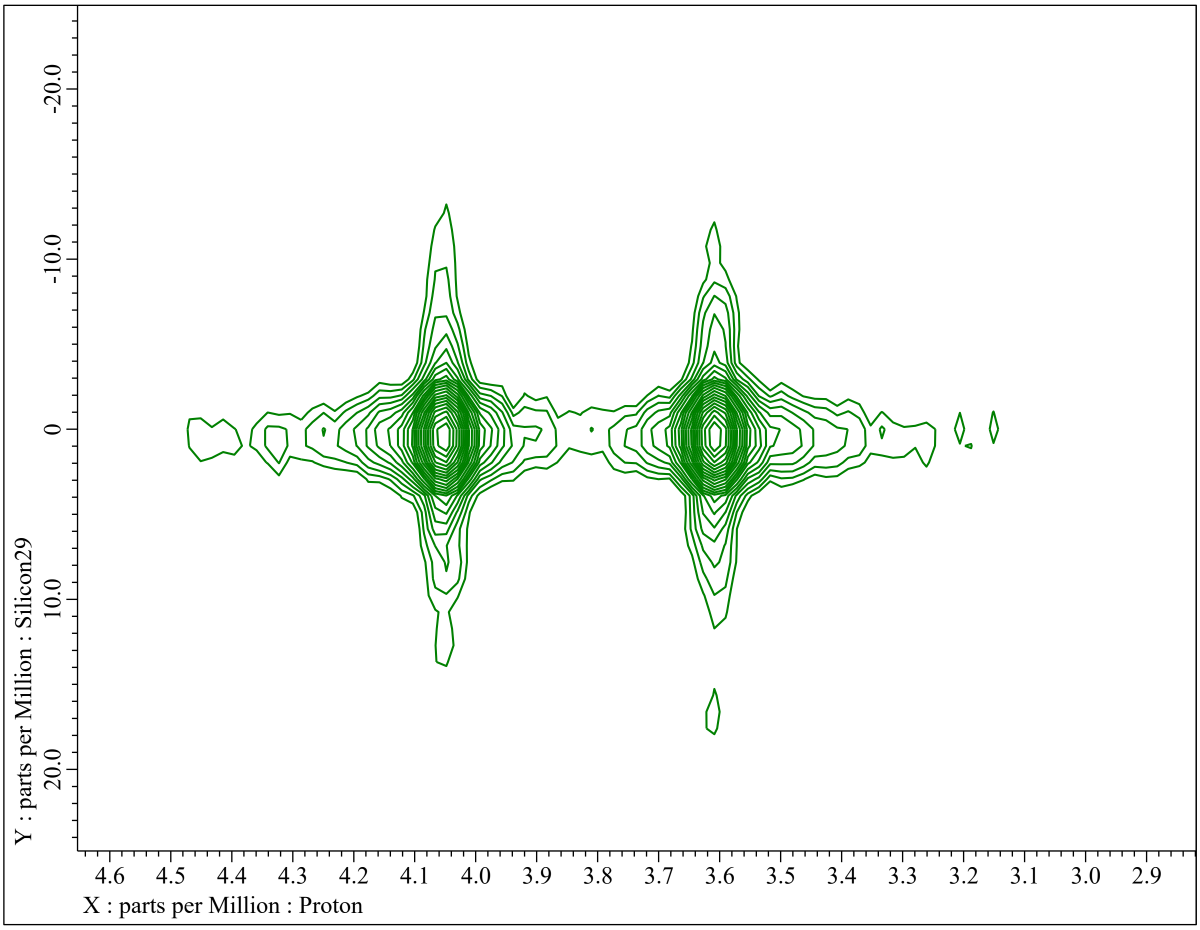

Figure S38. ^{1}H-^{29}Si HMQC spectrum of triethylsilane in benzene-D_6 solvent.

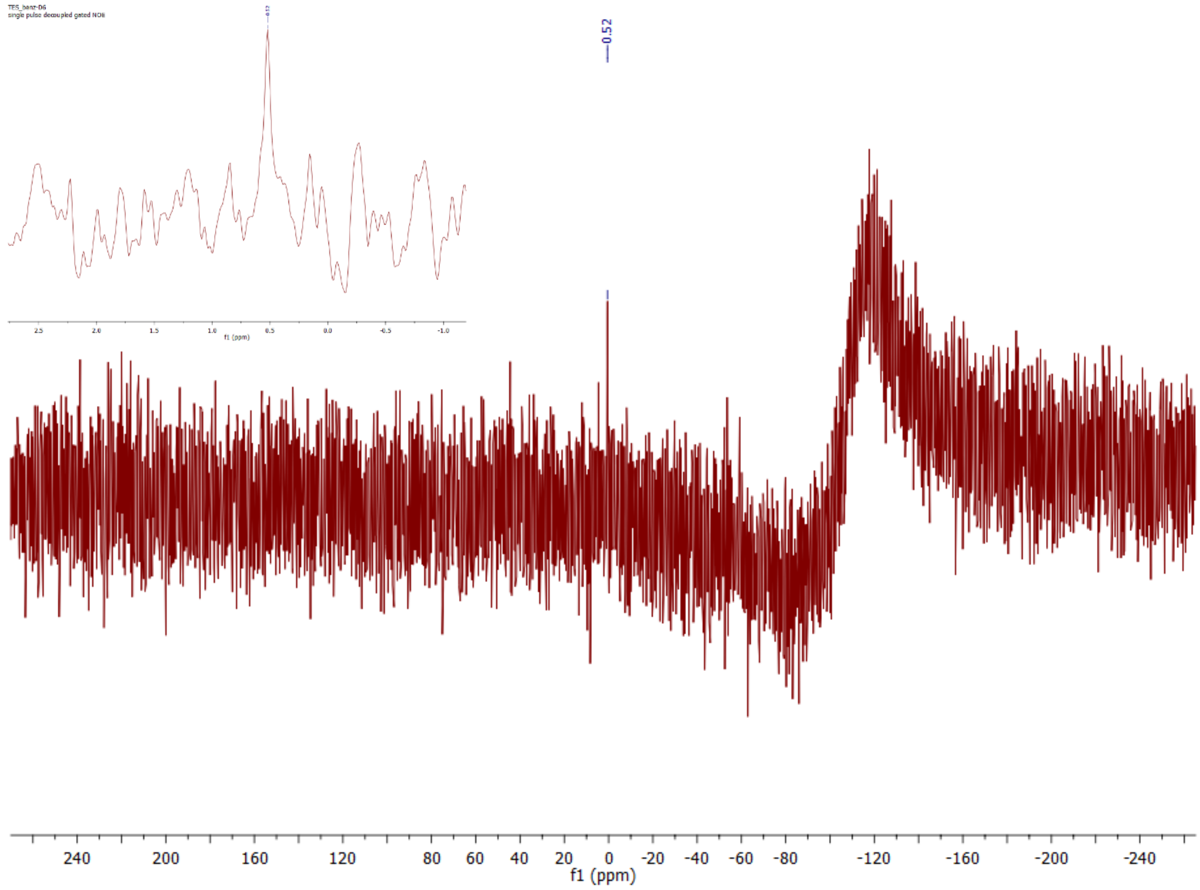

Figure S39. ^{29}Si spectrum of triethylsilane in benzene-D_6 solvent.

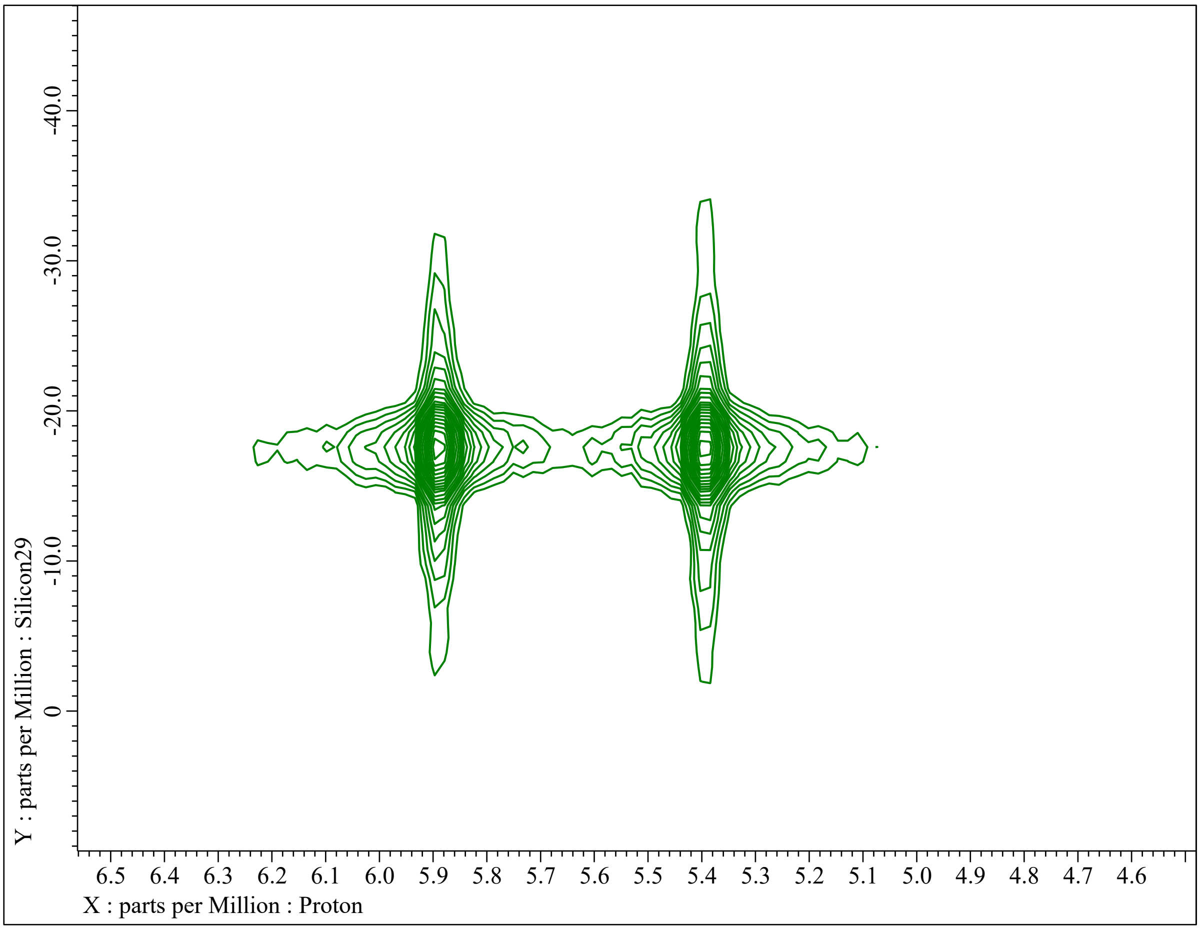

Figure S40. ^{1}H-^{29}Si HMQC spectrum of triphenylsilane in benzene-D_6 solvent.

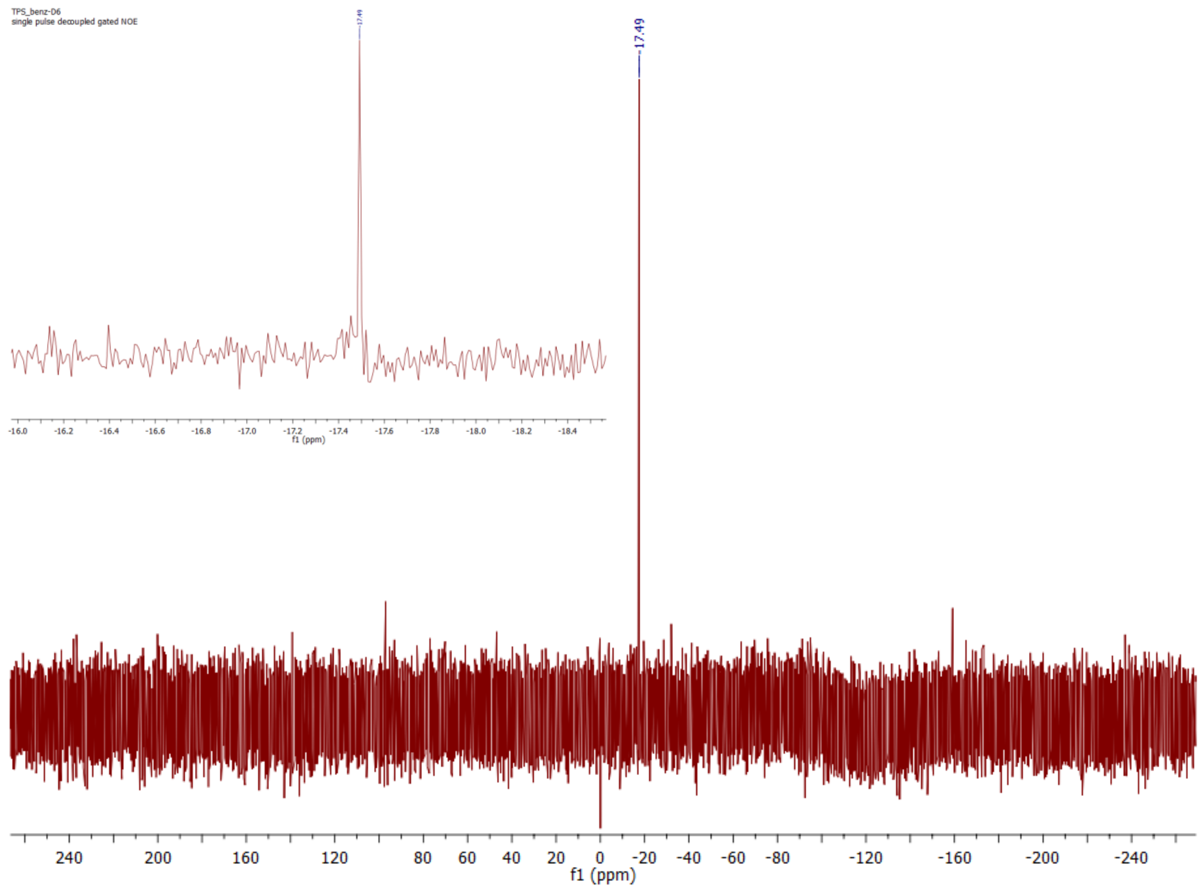

Figure S41. ^{29}Si spectrum of triphenylsilane in benzene-D_6 solvent.

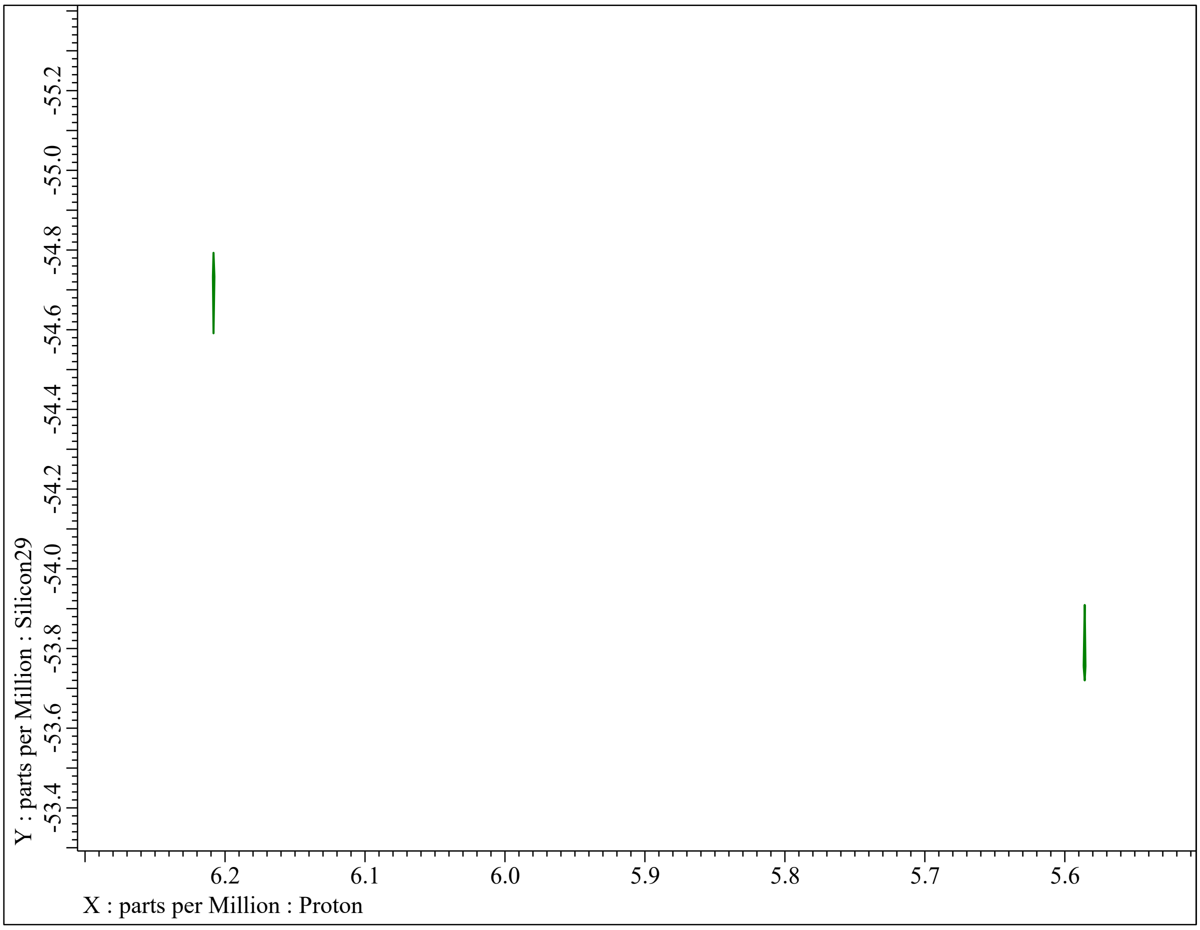

Figure S42. ^{1}H-^{29}Si HMQC spectrum of tris(pentafluorophenyl)silane in oDCB-D_4 solvent.

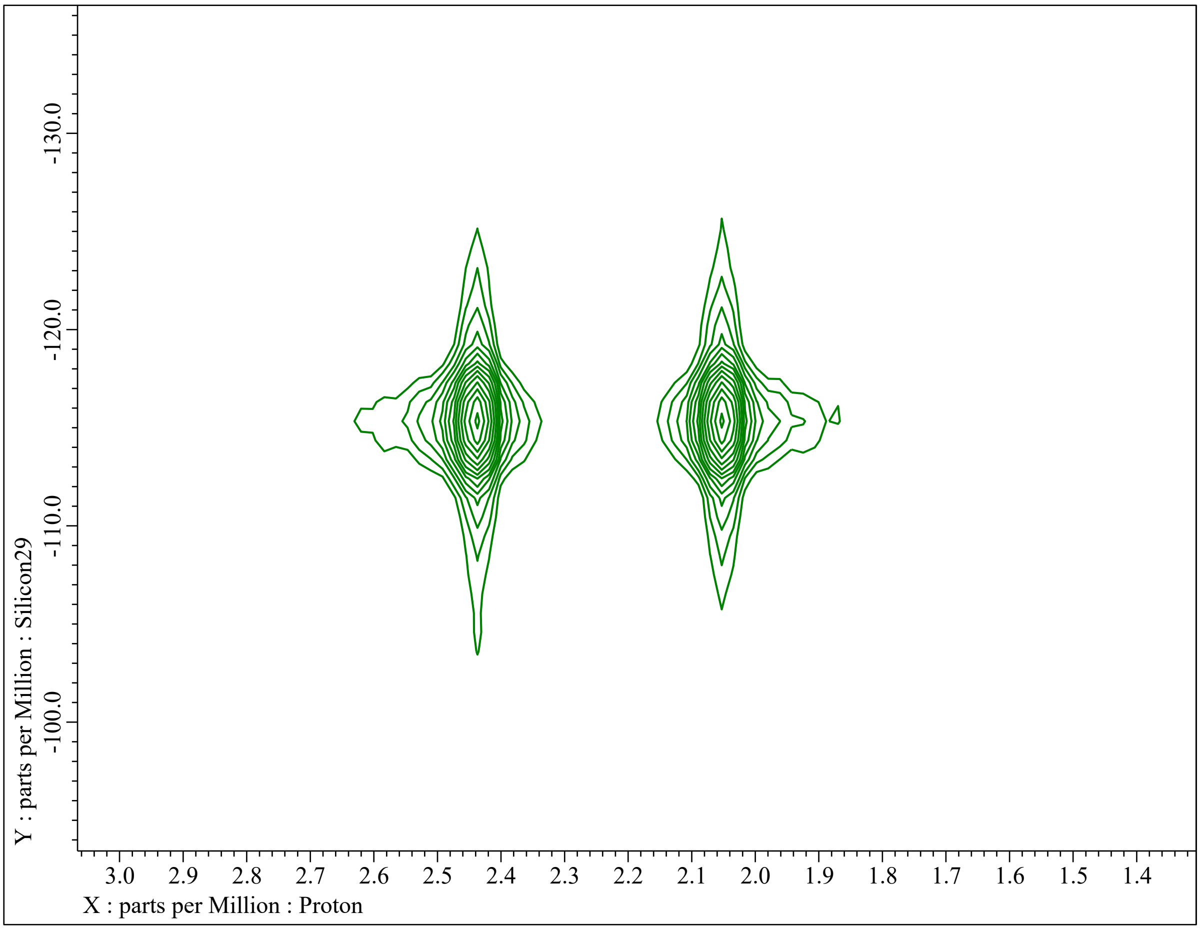

Figure S43. ^{1}H-^{29}Si HMQC spectrum of tris(trimethylsilyl)silane in oDCB-D_4 solvent.

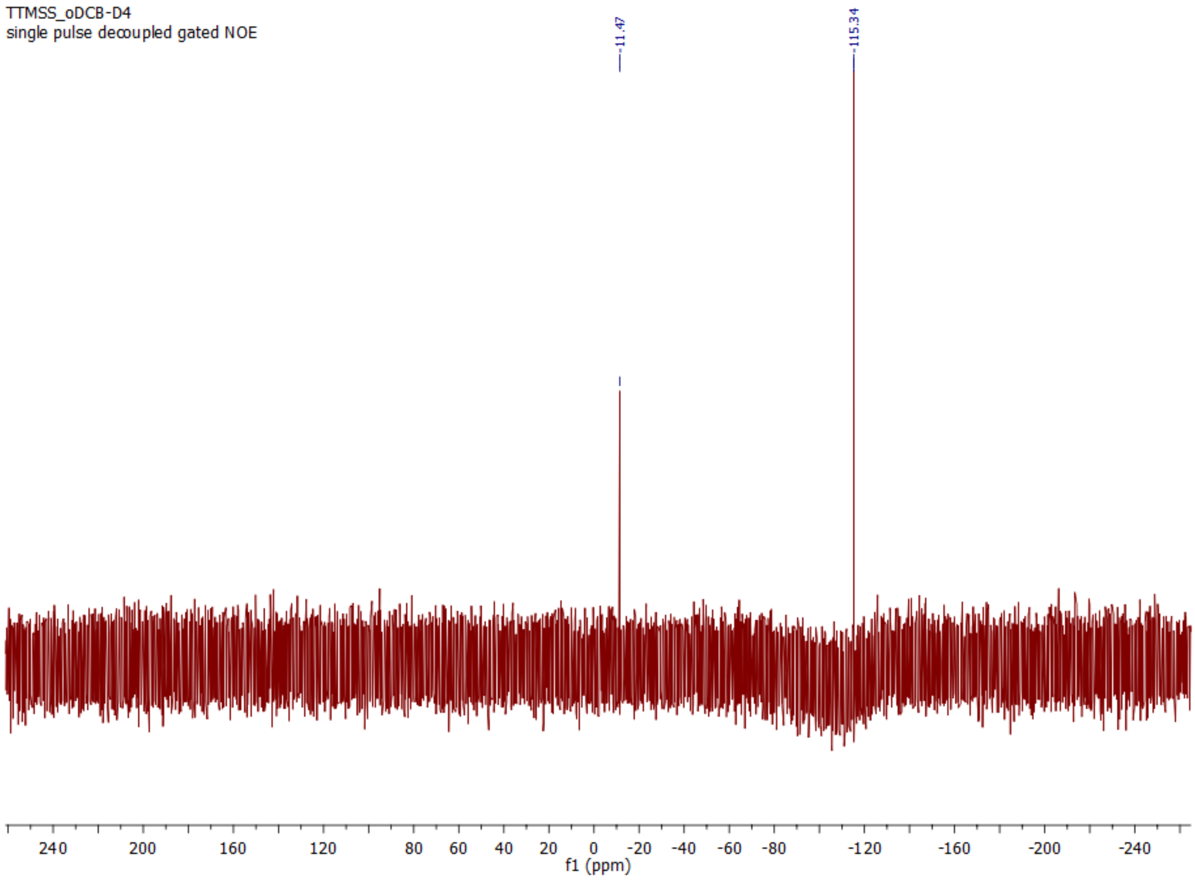

Figure S44. ^{29}Si spectrum of tris(trimethylsilyl)silane in oDCB-D_4 solvent.

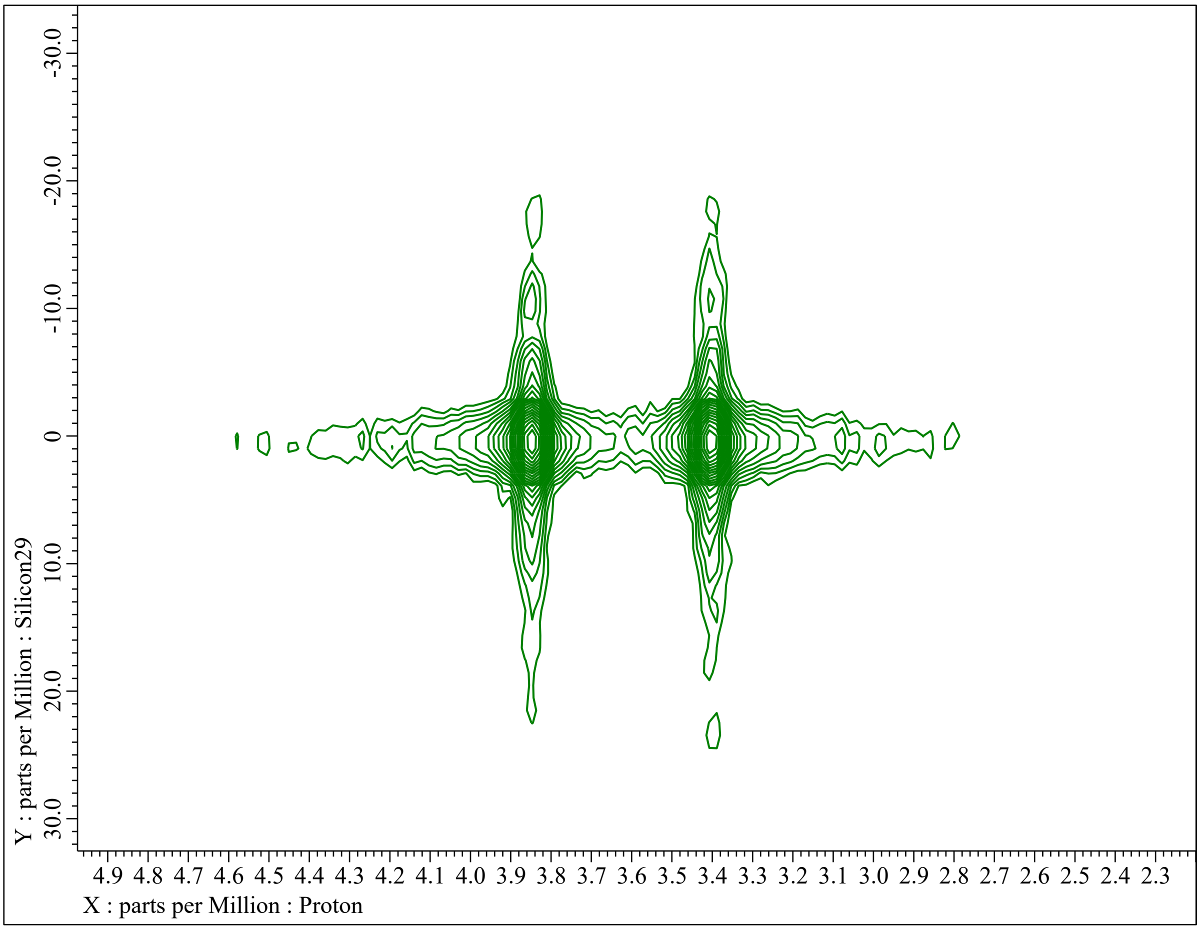

Figure S45. ^{1}H-^{29}Si HMQC spectrum of triethylsilane in oDCB-D_4 solvent.

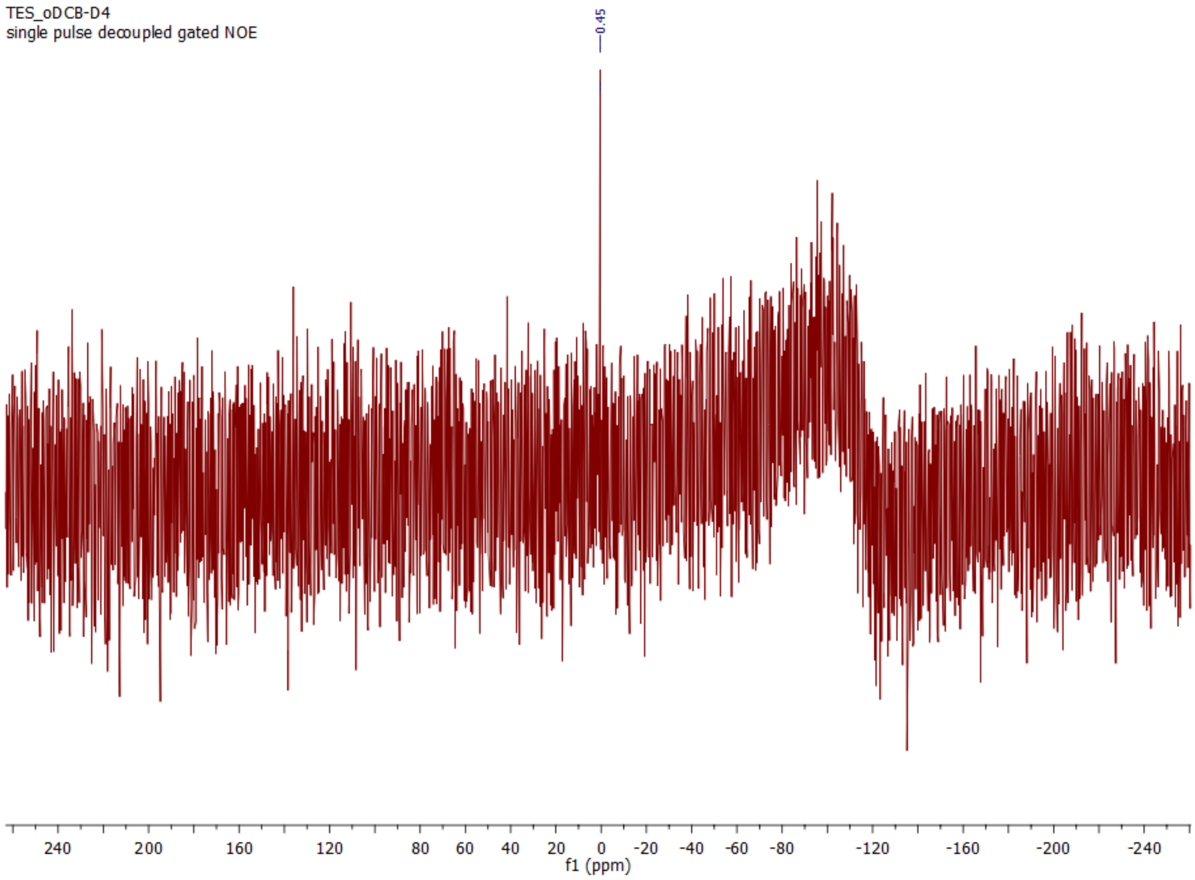

Figure S46. ^{29}Si spectrum of triethylsilane in oDCB-D_4 solvent.

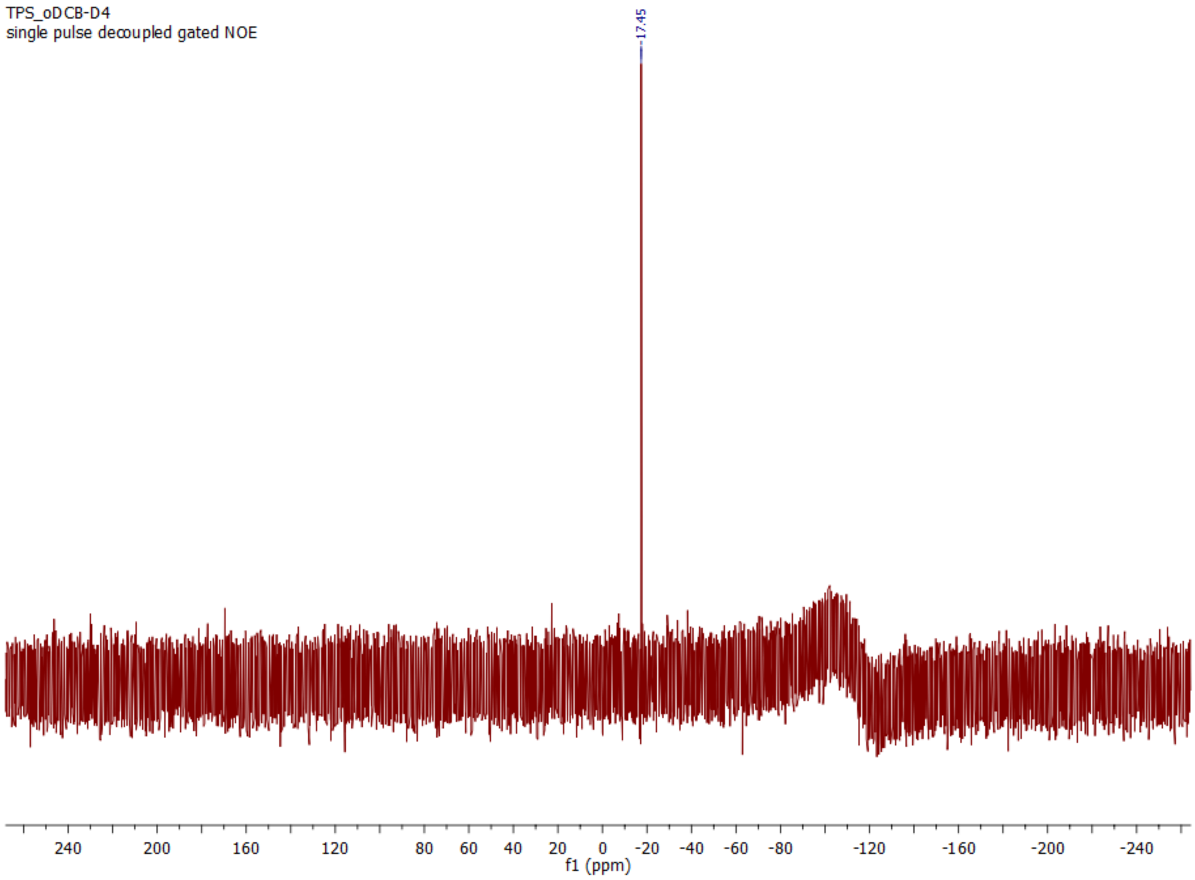

Figure S47. ^{29}Si spectrum of triphenylsilane in oDCB-D_4 solvent.

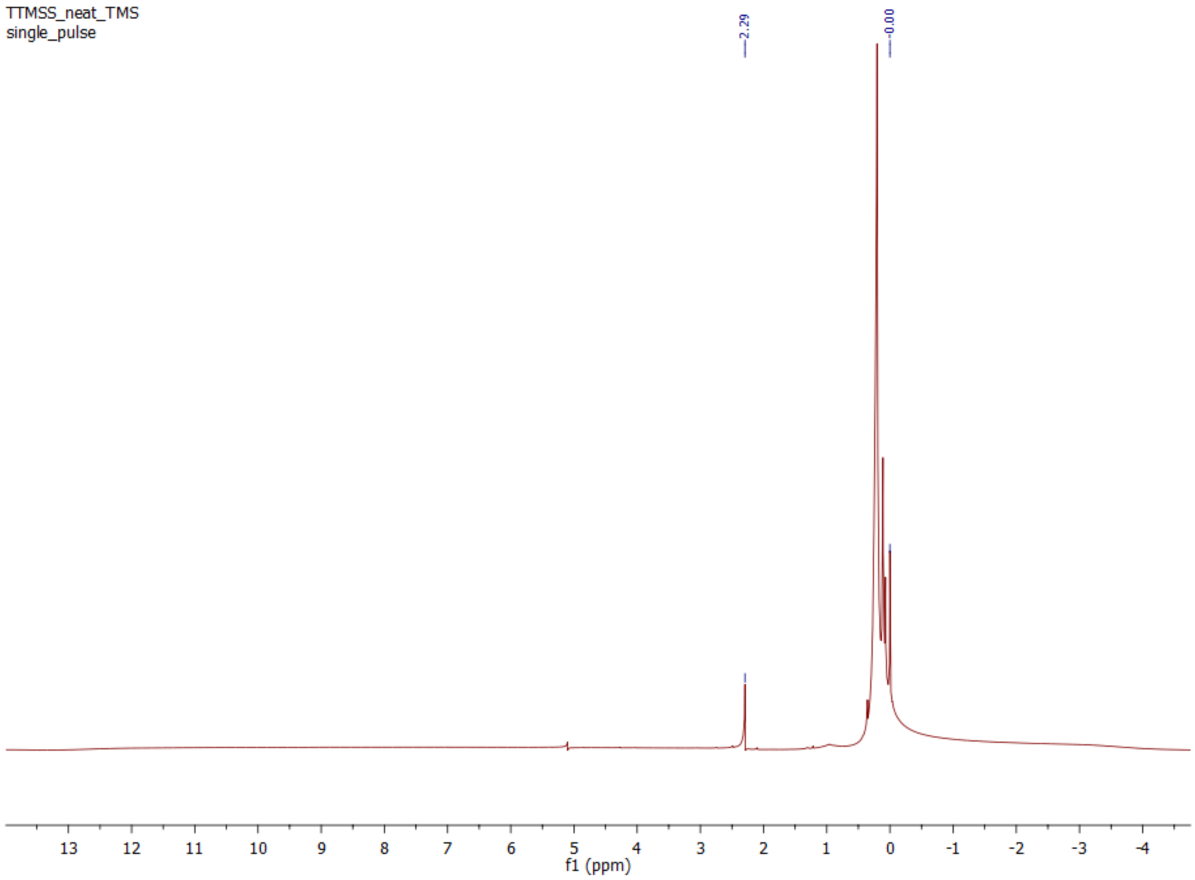

Figure S48. ^{1}H spectrum of neat tris(trimethylsilyl)silane with added TMS (0 ppm) for referencing.

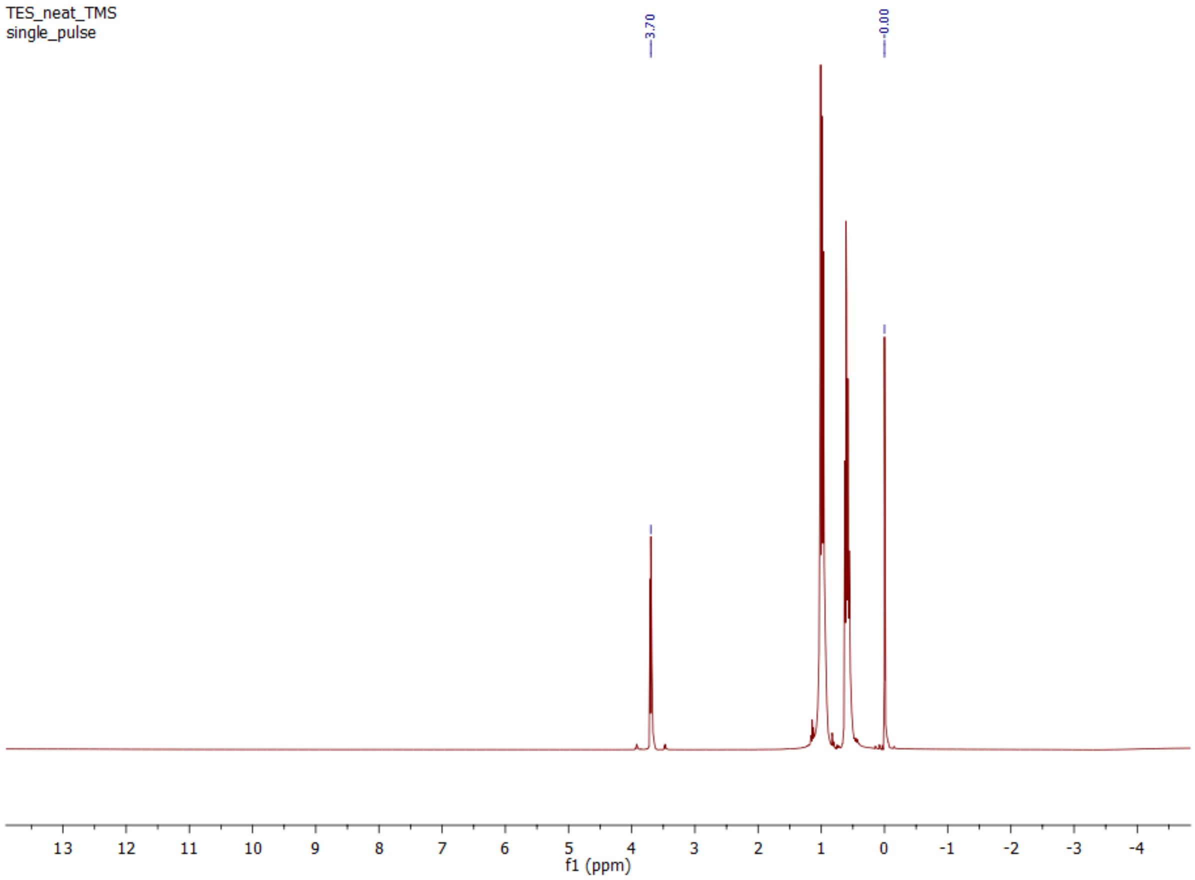

Figure S49. ^{1}H spectrum of neat triethylsilane with added TMS (0 ppm) for referencing.

Variable-temperature ^{1}H-NMR results

To further investigate the physical origin of the Si–H chemical shift trends and their relationship to the calculated electrostatic potentials (ESPs), we conducted variable-temperature (VT) ^{1}H NMR measurements for four representative silanes in two solvents (CD_2Cl_2 and benzene-D_6). These experiments provide additional insight into the distinct components of the temperature-dependent shielding and offer an experimental handle on the factors beyond local electron density that influence ^{1}H δ. Apart from ^{1}H δ(T) values, $^1J_{Si–H}$(T) were inspected as well. The results for the four inspected silanes are depicted in Figures S50-53.

<u>Model and physical interpretation</u>

The experimental δ(T) and $^1J_{Si–H}$(T) data were fitted to a mixed linear–inverse function of the general form:

$$y = a + b \cdot T + \frac{c}{T}$$

This model, a well-established empirical representation for VT NMR data, captures the three shielding contributions recognized in Ramsey's theory, where ***a*** is temperature-independent diamagnetic term (local electron density, ground-state geometry), ***bT*** is a term approximately linear in temperature, arising from bulk magnetic susceptibility (BMS) changes, vibrational averaging, and solvent–solute orientational/anisotropic interactions (particularly significant for aromatic media), and ***c/T*** is a Curie-type term, which reflects paramagnetic shielding contributions associated with virtual electronic excitations and thermally populated low-lying states. This term also captures temperature-dependent solvent–solute complexes whose population follows a ***1/T***-like dependence.

Thus, the VT analysis explicitly goes beyond an electron-density interpretation and incorporates additional mechanisms, including paramagnetic contributions influenced by virtual excitations.

<u>Solvent and substituent effects</u>

Most tested silanes exhibit opposite ***δ(T)*** curvature when comparing CD_2Cl_2 and benzene-D_6 solvents. This inversion cannot be explained by changes in local electron density or Si–H bond length; instead, it confirms that anisotropic solvent effects dominate the temperature dependence, particularly due to ring-current and quadrupolar anisotropy effects of benzene-D_6 solvent. The ***c*** parameter varies significantly between the two solvents, underscoring that the magnitude of the paramagnetic-like (***1/T***) term is solvent-mediated.

When ***b*** and ***c*** share the same sign, the competition between the ***bT*** and ***c/T*** terms yields an extremum:

$$T_{ext} = \sqrt{\frac{c}{b}}$$

The only meaningful extremum was found for tris(pentafluorophenyl)silane in benzene-D_6 ($T_{ext} \approx 307$ K). For the remaining systems, T_{ext} lies outside the accessible temperature range, or the ***δ(T)*** function is monotonic (see Table S15). The existence of an extremum directly demonstrates that paramagnetic and linear contributions are of comparable magnitude at lower temperatures, while linear mechanisms dominate at higher temperatures, consistent with the expected behavior of the ***c/T*** term.

Table S15. ^{1}H δ extremum temperatures for the studied silanes in CD_2Cl_2 and benzene-D_6 solvents.

silane	extremum temperature T_{ext} (K)	
	CD_2Cl_2	benzene-D_6
TES	1146.6	625.0
TTMSS	∄	435.9
TPS	426.6	758.6
TPFPS	∄	307.2

Despite the observed changes in ^{1}H chemical shifts with temperature, the $^{1}J_{Si–H}$ coupling constants vary only weakly with temperature across all samples. Given that $^{1}J_{Si–H}$ is sensitive to Si–H bond length, this finding demonstrates that changes in ^{1}H δ do not primarily arise from structural (bond-length) changes, and therefore, are more likely governed by electronic (paramagnetic + anisotropic) and solvent-field effects. This decoupling reinforces that the VT data probe a different set of physical factors than the ESP analysis.

Relation to DFT-calculated ESPs

In addition to the recorded chemical shift values recorded in varying solvents at 25 °C, the VT data, particularly the temperature-dependent parameters acquired through the fit, show very limited correlation with ESP. This suggests that while ESPs influence the static electronic environment, the temperature-dependent behavior is primarily determined by solvent anisotropy, vibrational averaging, and paramagnetic shielding mechanisms, which are largely independent of the ESP values. Thus, the VT results complement the ESP analysis by reflecting the various dynamic factors that perturb shielding around the equilibrium electronic environment governed by ESP.

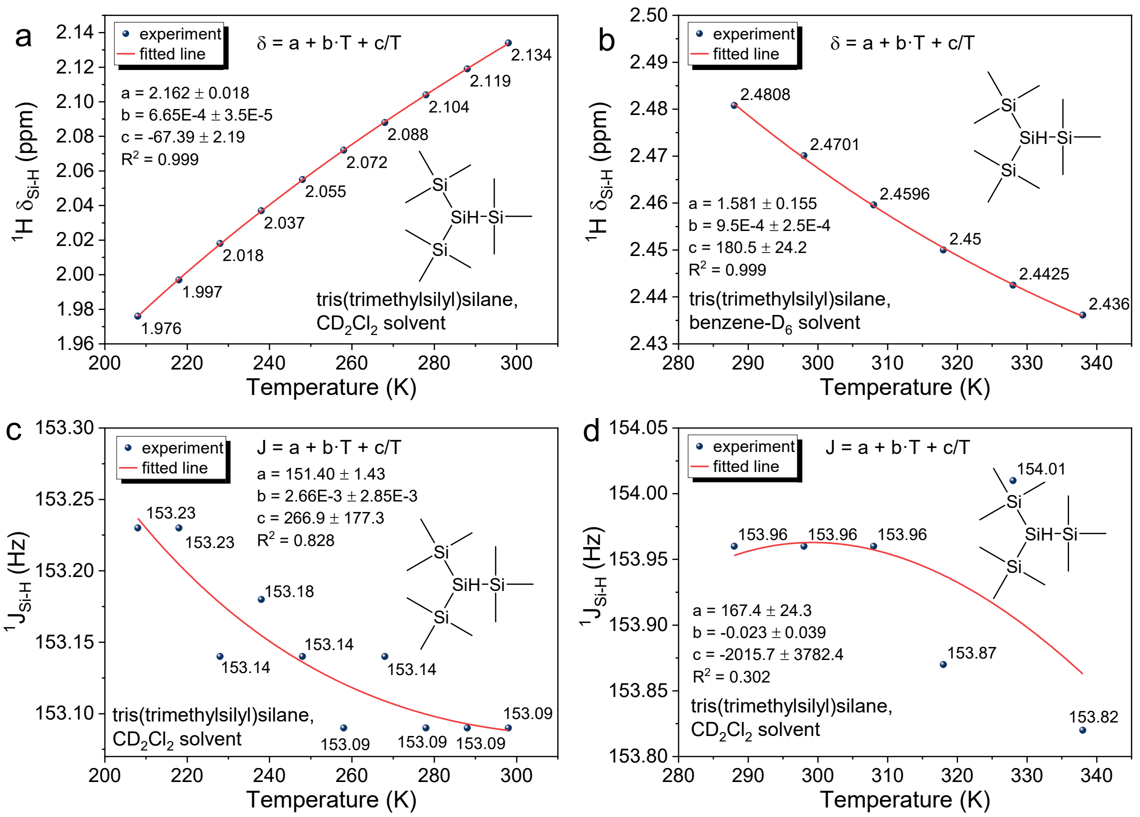

Figure S50. VT ^{1}H-NMR results for tris(trimethylsilyl)silane (TTMSS): temperature dependence of Si-H chemical shifts in CD_2Cl_2 (a) and benzene-D_6 (b) solvents, and temperature dependence of $^1J_{Si\text{-}H}$ constants in CD_2Cl_2 (c) and benzene-D_6 (d) solvents.

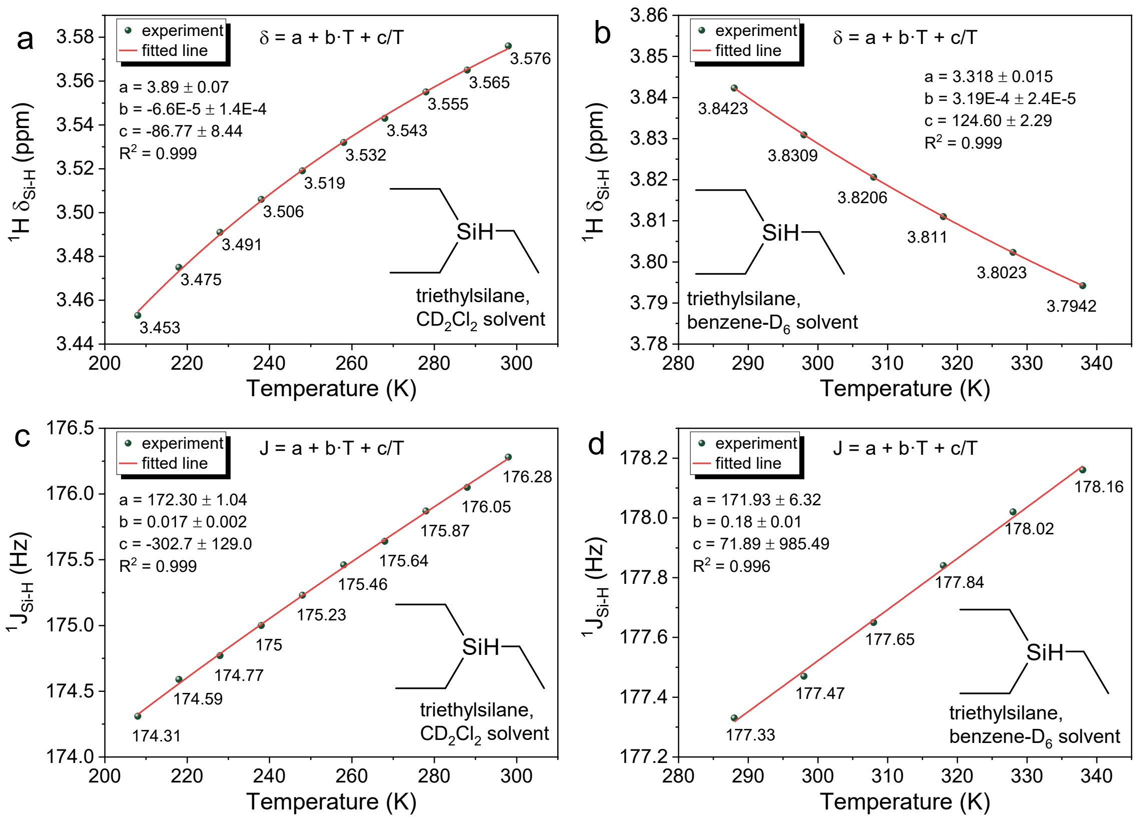

Figure S51. VT ^{1}H-NMR results for triethylsilane (TES): temperature dependence of Si-H chemical shifts in CD_2Cl_2 (a) and benzene-D_6 (b) solvents, and temperature dependence of $^{1}J_{Si-H}$ constants in CD_2Cl_2 (c) and benzene-D_6 (d) solvents.

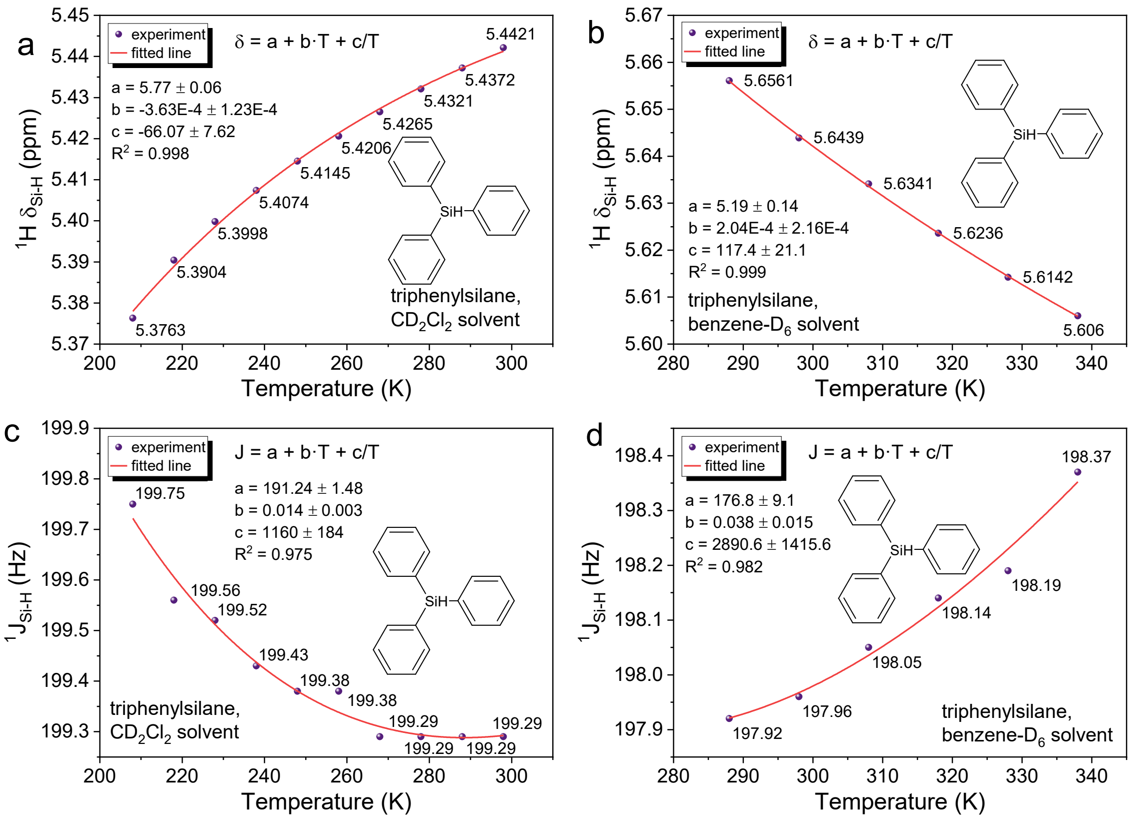

Figure S52. VT ^{1}H-NMR results for triphenylsilane (TPS): temperature dependence of Si-H chemical shifts in CD_2Cl_2 (a) and benzene-D_6 (b) solvents, and temperature dependence of $^1J_{Si-H}$ constants in CD_2Cl_2 (c) and benzene-D_6 (d) solvents.

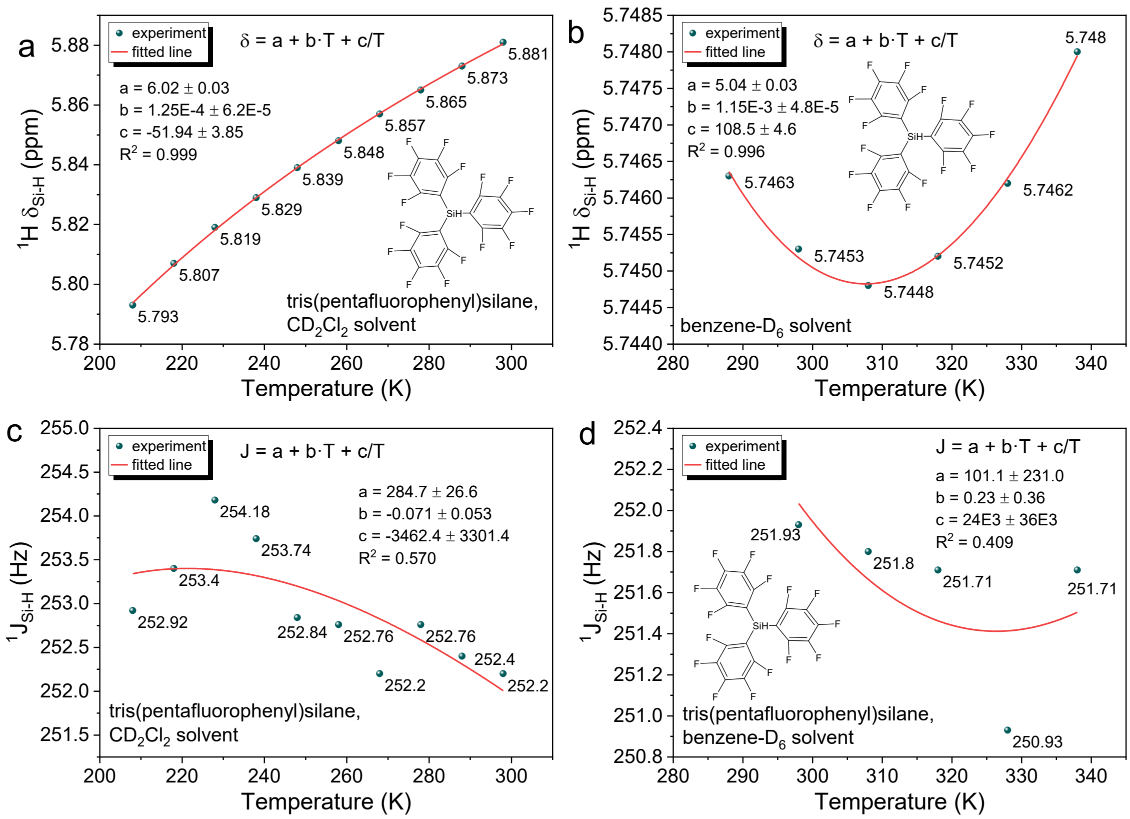

Figure S53. VT ^{1}H-NMR results for tris(pentafluorophenyl)silane (TPFPS): temperature dependence of Si-H chemical shifts in CD_2Cl_2 (a) and benzene-D_6 (b) solvents, and temperature dependence of $^1J_{Si\text{-}H}$ constants in CD_2Cl_2 (c) and benzene-D_6 (d) solvents.

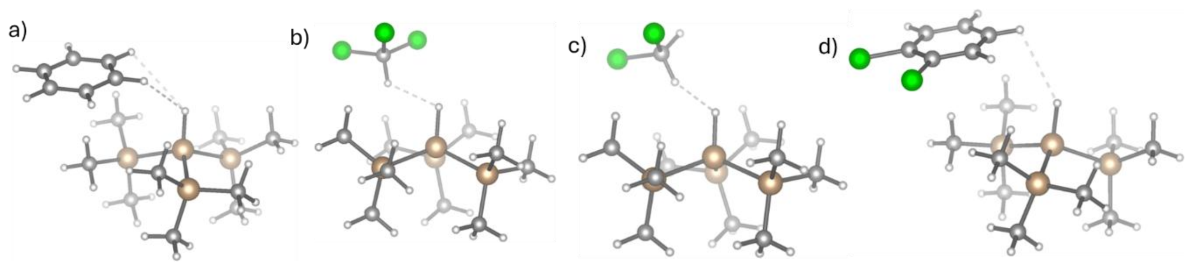

Figure S54. The optimized geometries of $(Me_3Si)_3Si$-H with a) ben, b) $CHCl_3$, c) CH_2Cl_2 and d) o-DCB. [C: grey, H: white, Si: golden, Cl: green]
